# Heterogenized Copper(II) Phenanthroline Catalysts for Electroreduction of CO_2_ to C_2_ Compounds: Substitution on the Ligand Causes Structural Changes to the Molecular Framework and Stability Enhancement

**DOI:** 10.1002/adma.202513702

**Published:** 2025-10-01

**Authors:** Na Liu, Trang Minh Pham, Yanan Han, Linfeng Yang, Olga S. Bokareva, Stephan Bartling, Armin Springer, Anke Spannenberg, Christoph Kubis, Jana Weiss, Dmitry E. Doronkin, Wen Ju, Robert Francke

**Affiliations:** ^1^ Leibniz Institute for Catalysis 18059 Rostock Germany; ^2^ Institute of Chemistry and Department of Life Light and Matter University of Rostock 18059 Rostock Germany; ^3^ Electron Microscopy Center University Medicine Rostock Strempelstr. 14 18057 Rostock Germany; ^4^ Institute for Chemical Technology and Polymer Chemistry and Institute of Catalysis Research and Technology Karlsruhe Institute of Technology 76131 Karlsruhe Germany

**Keywords:** copper, phenanthroline, electrocatalysis, molecular catalyst, CO_2_ reduction

## Abstract

Molecular Cu catalysts have shown promise for electrochemical CO_2_ reduction (eCO_2_RR) to multi‐carbon products. Unlike metallic Cu facets, they offer precise control over the active site's electronic and steric configuration. However, prior studies identified critical challenges related to irreversible potential‐induced formation of Cu particles, which participate in the eCO_2_RR and obscure the role of molecular motifs. Based on a previously reported binuclear Cu(II) phenanthroline catalyst, a structurally modified second‐generation system with enhanced stability is developed. By introducing methoxy groups to the phenanthroline ligand, the molecular framework changes from a binuclear complex to an oligonuclear step‐like structure consisting of Cu(II) ions linked by *µ*_2_‐ and *µ*_3_‐OH groups. When immobilized on a gas diffusion electrode, stable operation with a Faradaic efficiency of >70% for C_2_ products is achieved at elevated current densities. In situ XAS spectroscopy shows only negligible changes of the Cu coordination environment up to 50 mA cm^−2^. When approaching 250 mA cm^−2^, partial and reversible phase evolution occurs under Cu^2+^ valence state reduction, followed by phase recovery upon bias removal. This system combines structural robustness with adaptive redox behavior, demonstrating a route for implementing molecular electrocatalysts in eCO_2_RR processes at industrial current densities.

## Graphical abstract

Based on a previously reported binuclear Cu(II) phenanthroline catalyst, a structurally modified version with enhanced stability for electrochemical reduction of CO_2_ is developed. The second‐generation system allows for stable operation in an MEA electrolyzer with a Faradaic efficiency of >70% for C_2_ products at elevated current densities. In situ XAS spectroscopy provided mechanistic insights.

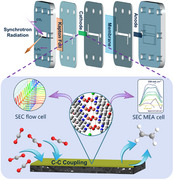

## 1 Introduction

The capture and conversion of CO_2_ emissions into value‐added chemicals holds immense potential for closing the carbon cycle.^[^ ^1^ ^]^ Among various available approaches, the electrochemical CO_2_ reduction reaction (eCO_2_RR) has attracted significant attention due to its ability to directly utilize surplus renewable electricity for the valorization of CO_2_. Depending on the employed catalyst and reaction conditions, this approach generates valuable products such as carbon monoxide (CO), formate (HCOO^−^), ethylene (C_2_H_4_), and various multi‐carbon oxygenates.^[^ ^2^ ^]^ Recent advances in electrolyzer design have enabled two‐electron reduction processes (i.e., CO and formate generation) to be operated under industrially relevant conditions, which could facilitate commercialization in the near future.^[^ ^2a,c^ ^]^ In contrast, the electrochemical synthesis of multi‐carbon products (e.g., C_2_H_4_ and C_2_H_5_OH) faces persistent fundamental and technical challenges. These hurdles arise from i) complicated reaction networks involving multiple electron transfers, protonation steps, C─O bond cleavage, and C─C coupling,^[^ ^3^ ^]^ ii) the complex interplay between microkinetics, mass transfer, and durability on the device level at industrially relevant current densities,^[^ ^4^ ^]^ and iii) structural and chemical instability of state of the art catalysts under operating conditions.^[^ ^5^ ^]^

Cu‐based catalysts remain the benchmark for eCO_2_RR to C_2+_ products owing to their unique capability to stabilize ^*^CO intermediates and facilitate C─C bond formation.^[^ ^6^ ^]^ However, the situation is complicated by various Cu sites (terraces, steps, defects) with different selectivity,^[^ ^7^ ^]^ while dynamic phase evolution under operating conditions often leads to a variation of the product distribution over time.^[^ ^8^ ^]^ Overall, these aspects lead to performance instability, hinder the mechanistic understanding, and complicate the targeted optimization strategies.

To address the challenges along the way to efficient C_2+_ production, the development of well‐defined catalyst structures is a prominent strategy. In principle, such structures should enable precise elucidation of structure‐performance relationships–particularly for the desired C─C coupling step–while providing opportunities for performance and durability optimization. In this regard, molecular electrocatalysts with well‐defined active sites provide interesting opportunities. While many reported molecular systems have achieved high Faradaic Efficiency (*FE*) to CO^[^ ^9^ ^]^ and HCOOH^[^ ^10^ ^]^ by using earth‐abundant metals such as Fe, Mn, and Co, relatively few works have demonstrated the possibility for generating C_2+_ products using molecular Cu catalysts.^[^ ^11^ ^]^

**Scheme 1:**
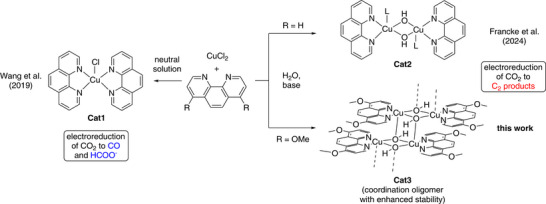
Mono, bi‐ and oligonuclear Cu‐phen compounds as molecular catalysts for eCO_2_RR.^[^ ^9^, ^16^ ^]^

The work to date highlights two critical aspects for the development of new molecular Cu catalysts for the formation of C_2+_ products. First, it appears advantageous when the molecular framework provides two or more vicinal Cu centers enabling C─C coupling between CO_2_ reduction intermediates. Consequently, bi‐ or multinuclear Cu complexes with suitable Cu···Cu distance have shown particularly high selectivity toward C_2+_ products.^[^ ^12^ ^]^ Second, a robust and cost‐effective ligand with suitable steric and electronic properties must be selected. The bidentate 1,10‐phenanthroline ligand (phen) was identified as an excellent choice for stabilizing bi‐ or multinuclear Cu ensembles due to strong and entropically favored binding to metal sites,^[^ ^13^ ^]^ an excellent π acceptor capability that stabilizes lower metal oxidation states,^[^ ^14^ ^]^ and a particularly high affinity to Cu ions.^[^ ^15^ ^]^ Both the influence of polynuclear structural motifs and the stabilizing effect of the phen ligand are well‐documented by previous studies on the Cu phen system (**Scheme** **1**). While Wang et al. have introduced **Cat1** as a robust catalyst for the generation of C_1_ products (CO and HCOO^−^ in up to 90% combined *FE*),^[^ ^9a^ ^]^ the binuclear **Cat2** recently reported by us shows a strong tendency to form C_2_ products.^[^ ^16^ ^]^ When immobilized on a carbon electrode, our easy‐to‐synthesize phen‐based **Cat2** shows 62% FE_C2+_ during electrolysis in an H‐type divided batch cell. Structural characterization (pre‐ and post‐ electrolysis) revealed a hydroxo‐bridged binuclear framework with a Cu···Cu distance of 2.93 Å. A remaining challenge in the use of **Cat2** for eCO_2_RR is slow leaching into the electrolyte solution, which is why stable long‐term operation in a membrane‐electrode assembly (MEA) electrolyzer under continuous flow conditions could not be achieved. Consequently, we aimed to develop a more stable system by modifying the phen ligand, which we achieved by introducing methoxy substituents in positions 4 and 7 (**Cat3**, see Scheme 1).

Herein, we present a study on the oligonuclear Cu phen compound **Cat3**, a distinct Cu^2+^ complex synthesized from 4,7‐dimethoxy‐1,10‐phenanthroline and CuCl_2_ using a straightforward and scalable protocol. **Cat3** features hydroxo‐bridged Cu centers that form a cascade‐like framework. Compared to **Cat2**, water insolubility is achieved, accompanied by enhanced robustness under electrocatalytic conditions. Taken together, this allows for integrating **Cat3** into an MEA electrolyzer for studies under technically relevant conditions. Remarkably, **Cat3** achieves above 70% *FE*_C2+_ at current densities up to 200 mA cm^−2^ in the MEA electrolyzer. Stable operation was realized for 10 h with no obvious degradation in performance. To fundamentally address uncertainties about the state of our proposed molecular active Cu sites, in situ XAFS and HERFD‐XANES analyses were carried out using both an H‐type and an MEA‐type spectroelectrochemical cell to explore different current density regions. As a result, merely negligible changes in the Cu coordination environment were observed up to 50 mA cm^−2^, while partial and reversible phase evolution under Cu^2+^ valence state reduction occurs when approaching 250 mA cm^−2^. Interestingly, the initial phase recovers upon bias removal, indicating reversible adaptive redox dynamics under reaction conditions. This work not only establishes a robust molecular catalyst for the selective production of C_2_ compounds, but also advances operando XAS methodologies for investigating molecular electrocatalysts under industrially relevant current densities.

## 2 Results and Discussion

### 2.1 Synthesis and Structural Characterization

**Figure 1:**
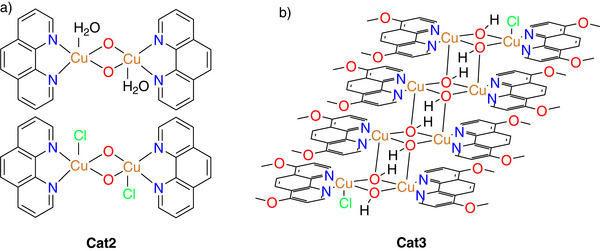
a) The structure of our previously reported **Cat2** and b) the core structure (coordination octamer) obtained after crystallization of **Cat3**.

Based on our previously reported protocol for the synthesis of **Cat2** (**Figure** **1**a),^[^ ^16^ ^]^ three Cu catalysts with differently substituted phen ligands were prepared from CuCl_2_ in alkaline solution (for details, see the Supporting Information) and screened with respect to their eCO_2_RR activity. Among the derivatives evaluated, 4,7‐dimethoxy‐substituted phen emerged as optimal, which is why all further efforts were focused on the resulting material (**Cat3**, see Scheme 1).

The molecular structure of **Cat3** was investigated using single‐crystal X‐ray diffraction (SC‐XRD). However, different approaches to growing single crystals unfortunately resulted in samples of limited quality, likely due to the formation of coordination oligomers of varying chain lengths. One of these oligomers could still be selectively crystallized and used for deducing the structure of the molecular core unit from the SC‐XRD results. In the analyzed solid state, the Cu compound adopts an octameric architecture (Figure 1b; Figure S1, Supporting Information), in which eight Cu^2+^ ions form a cascade‐like framework stabilized by μ‐oxo bridges, with peripheral methoxy‐substituted phenanthroline ligands and two terminal chloro ligands completing the coordination sphere. Nonetheless, due to the limited SC‐XRD data quality, distinguishing between oxo (Cu─O─Cu) and hydroxo (Cu─OH─Cu) bridges, as well as precisely determining the counterions and solvent molecules, was not possible. Therefore, complementary spectroscopic and theoretical approaches were used to resolve these structural ambiguities. In this context, X‐ray photoelectron spectroscopic (XPS) analysis shows the exclusive presence of oxygen within μ‐hydroxo bridges and methoxy groups (Figure S4, Supporting Information). Protonation of the bridging oxygen atoms was also probed using DFT calculations. For this purpose, the optimized structures of both the protonated and the non‐protonated forms of the model dimer, tetramer, and octamer were compared to each other. The results indicate that protonation occurs preferentially on the bridging oxygen atoms, leading to a chemically reasonable model with eight protons per octamer and suggesting that protonation drives overall electronic stabilization of the coordination compound (Figure S5, Table S1, Supporting Information). X‐ray absorption spectroscopy (XAS) of the as‐prepared material confirms the existence of Cu in the indicated coordination environment and excludes inorganic or metallic phases (Figures S2 and S3, Supporting Information).

For the preparation of electrodes for H‐cell and MEA measurements, inks consisting of **Cat3** and an ionomer in a water‐*i*PrOH mixture were applied to carbon by drop casting or spray coating. To confirm the identity of the catalyst in the different states, powder and catalyst films were subjected to XPS, XAS, Raman spectroscopy, and powder XRD (P‐XRD). A comparison to the results obtained from **Cat3** crystals suggests that the catalyst exists in the same structural states in all three forms (Figures S3 and S6–S10, Supporting Information).

To analyze the morphology of the crystals and the catalyst‐coated electrode as well as the elemental distribution across the surface on the µm level, scanning electron microscope (SEM) measurements coupled with energy‐dispersive X‐ray (EDX) spectroscopy were conducted. SEM images of the studied samples display needle‐like crystals, with C, N, O, and Cu species exhibiting a homogeneous distribution in the probed domains (Figures S11 and S12, Supporting Information). The nanostructure was confirmed by scanning transmission electron microscopy (STEM) at lower magnifications while a regular, yet amorphous structure was observed at higher magnifications (Figure S13, Supporting Information).

### 2.2 Studies in an H‐type Divided Cell

**Figure 2:**
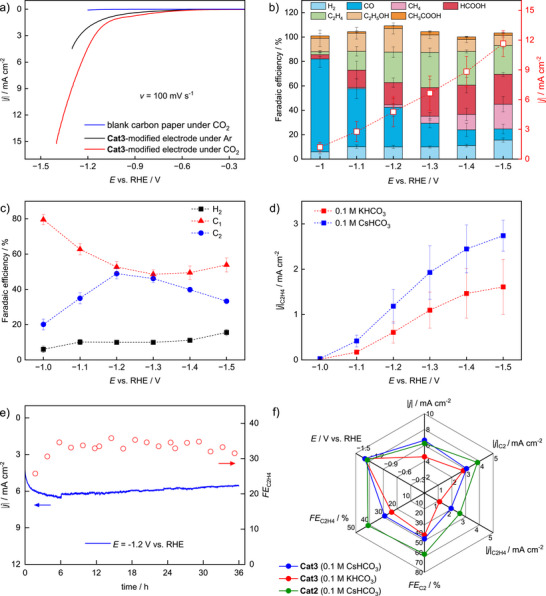
Electrochemical characterization of **Cat3**‐modified carbon paper electrodes (aq. 0.1 m CsHCO_3_ electrolyte, H‐type‐divided cell). a) LSVs of a **Cat3**‐modified electrode under Ar (black, pH 8.9) and CO_2_ (red, pH 6.8), respectively. b) Product distributions obtained upon electrolysis at different working electrode potentials using a **Cat3**‐modified electrode. c) Comparison of the *FE* values for the generation of C_1_ and C_2_ products as well as H_2_. d) Comparison of the partial current densities for C_2_H_4_ obtained from CPE in 0.1 m CsHCO_3_ and 0.1 m KHCO_3_ using a **Cat3**‐modified electrode. e) Long‐term stability test of **Cat3** at −1.2 V versus RHE. f) Comparison between eCO_2_RR key performance indicators showing the influence of catalyst structure (**Cat2** vs. **Cat3**) and supporting electrolyte (CsHCO_3_ vs KHCO_3_). All measurements were carried out in H‐cells at the indicated working electrode potential under otherwise identical conditions.

The eCO_2_RR activity of a **Cat3**‐modified carbon paper electrode was studied in a gas‐tight H‐type divided cell using an aqueous 0.1 M CsHCO_3_ electrolyte (**Figure** **2**; Figures S14–S19, Supporting Information). Linear sweep voltammetry (LSV) curves were recorded under both CO_2_ and Ar atmosphere, respectively (Figure 2a). Under CO_2_, **Cat3** exhibits a more positive onset potential and enhanced current density compared to the response under Ar, demonstrating promising catalytic activity.

To analyze the relationship between product distribution and working electrode potential, controlled potential electrolysis (CPE) was conducted at different potentials between −1.0 and −1.5 V versus RHE in CO_2_‐saturated solution (Figure 2b). While the gaseous products (mainly C_2_H_4_, CH_4_, CO, and H_2_) were detected online using a gas chromatograph (GC) after holding the potential for 14 min, the liquid‐phase products (formate, ethanol, and acetate) were analyzed using ^1^H NMR spectroscopy (for details, see the Supporting Information). At less negative potentials, CO generation is pronounced, but ceases in favor of formate and CH_4_ as the driving force increases. C_2_H_4_ generation begins at −1.0 V and gains significance with increasing potential. At −1.3 V and more negative potentials, C_2_H_4_ becomes the predominant product, with up to 2.7 mA cm^−2^ partial current density and 30% *FE*. Interestingly, ethanol formation is also relatively strong with *FE* values up to 20%. It should be noted that *FE*_H2_ remains ≈10% across the studied potential region, highlighting the preference toward eCO_2_RR over HER (which is consistent with our previously reported **Cat2**).^[^ ^16^ ^]^ Analysis of the *FE* for H_2_, C_1_ and C_2_ products at different potentials (Figure 2c) shows that the charge consumption for generating C_1_ and C_2_ products is almost equal in the range between −1.2 and −1.3 V (below and above this regime, formation of C_1_ products is dominant). Control experiments with a blank carbon paper electrode as well as with CuCl_2_ and Cu nanoparticle loadings show clearly different product distributions (see Figure S18, Supporting Information).

A comparison between the partial current density for C_2_H_4_ achieved in 0.1 m CsHCO_3_ and 0.1 m KHCO_3_ (2.7 and 1.6 mA cm^−2^, both at −1.5 V) highlights the advantageous effect of Cs^+^ ions (Figure 2d), similar to our previous study on **Cat2**.^[^ ^16^ ^]^ The promoting effect of Cs^+^ ions on eCO_2_RR has been reported for various electrocatalysts elsewhere. It has been ascribed to i), the weaker hydration shell compared to smaller cations, decreasing the interfacial pH and increasing local CO_2_ concentration, ii), improved surface charge‐dependent reaction energetics associated with the smaller hydrated ion radius, and iii), the stabilization of key eCO_2_RR intermediates.^[^ ^17^ ^]^ The effect of Cs^+^ on the behavior of **Cat3** was studied using impedance spectroscopy and is treated in detail in the SI (see Figure S20, Supporting Information; with corresponding discussion).

Noteworthy, **Cat3** demonstrates exceptional electrocatalytic stability, maintaining stable *FE*_C2H4_ and nearly constant current density at −1.2 V for over 36 h of continuous operation (Figure 2e; Figures S21–S23, Supporting Information). In Figure 2f, we compare the eCO_2_RR performance with the maximum *FE*_C2H4_ of **Cat3** with our previous **Cat2** in CsHCO_3_. While **Cat2** seems to outperform **Cat3** in the H‐cell configuration, the incorporation of the methoxy substituents significantly enhances the robustness under continuous flow conditions, enabling stable operation in an MEA electrolyzer at high current densities (vide infra). It should also be noted that in the H‐cell, **Cat3** exhibits significantly reduced leaching. While ICP‐OES analysis of the post‐electrolysis electrolyte solution revealed a Cu ion concentration of 2.5 mg mL^−1^ in the case of **Cat2**,^[^ ^16^ ^]^ only 0.03 mg mL^−1^ was found under the same conditions in the case of **Cat3**.

### 2.3 Post‐Electrolysis Characterization of the Electrocatalyst

**Figure 3:**
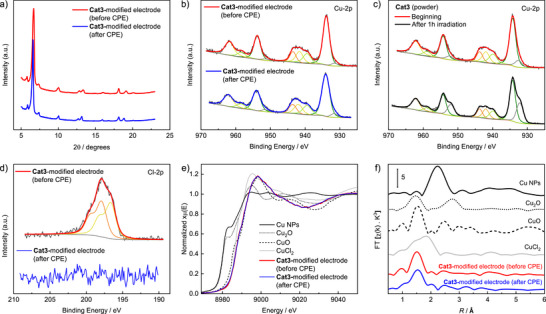
a) P‐XRD patterns of **Cat3** after electrolysis. b–d) XPS analysis. Scans in the Cu 2p region using **Cat3**‐modified carbon paper electrode before and after electrolysis, monitoring of the progression of the Cu 2p spectrum of as‐prepared **Cat3** powder during X‐ray irradiation, and scans in the Cl 2p region. e,f) XAS analysis of **Cat3**‐modified carbon paper electrode (before and after electrolysis) and of reference materials. Normalized Cu K‐edge XANES spectra and Fourier transform EXAFS spectra.

After CPE at −1.2 V in CO_2_‐saturated 0.1 m CsHCO_3_ solution, the working electrode was analyzed using P‐XRD, SEM‐EDX, XPS, and XAS (**Figure** **3**; Figures S24–S31, Supporting Information). Interestingly, P‐XRD analysis of the electrode after electrolysis revealed the same reflection peaks as those of the as‐prepared **Cat3** electrode, indicating the preservation of crystalline domains during electrolysis. This finding is consistent with SEM results (Figures S25–S28, Supporting Information), which show microcrystalline objects on the surface (in contrast, the binuclear complex **Cat2** previously investigated by us transitioned completely to the amorphous state during electrolysis).^[^ ^16^ ^]^ EDX mapping indicates even distributions of Cu, N, O, C, and Cl across the electrode surface. Notably, the Cl content decreased significantly after electrolysis compared with freshly prepared electrodes, indicating reductive dehalogenation of **Cat3** at negative potentials (as observed for **Cat2** in our previous study).

As shown in Figure 3b, XPS analysis of the pre‐ and the post‐electrolysis materials shows two main peaks at 934.2 and 954.2 eV, accompanied by pronounced satellite features in the Cu 2p region, which can be assigned to Cu 2p_3/2_ and Cu 2p_1/2_ in the Cu^2+^ state.^[^ ^18^ ^]^ The peaks at lower binding energies (932.2 and 952.1 eV) are associated with Cu^+^/Cu^0^ and result from reduction through X‐ray radiation under high‐vacuum conditions during the measurement.^[^ ^16^ ^]^ This interpretation is confirmed by repeating the XPS measurement of the Cu 2p region after 1 h irradiation, whereby the Cu^+^/Cu^0^ peaks are significantly enhanced (Figure 3c). The XPS element quantification results are shown in Table S4 (Supporting Information), including the N/Cu ratio, which remains constant for the fresh and used **Cat3**‐modified electrode, thus underlining the enhanced stability of the catalyst. In agreement with the EDX mapping results, the signal of the chloride ligand in the post‐electrolysis Cl 2p scan is missing (Figure 3d), indicating reductive cleavage of Cu─Cl bonds under eCO_2_RR conditions.

The oxidation state and coordination environment of the Cu species within **Cat3** were examined using ex situ X‐ray absorption spectroscopy (XAS, for experimental details, see the Supporting Information). Figure 3e shows the normalized Cu K‐edge X‐ray absorption near‐edge structure (XANES) spectra of **Cat3**, Cu_2_O, CuO, CuCl_2_, and Cu nanoparticles. A comparison shows that the reference materials display clear distinctions compared to our synthesized **Cat3**, showing an absorption edge ≈8975 eV. Toward the higher energy region, the K‐edge spectrum of **Cat3**, both before and after electrolysis, partially aligns with those of CuO and CuCl_2_ but lacks the characteristic feature at 8986 eV. The smooth rising edge without a shoulder at 8986 eV is typical for Cu^2+^ coordinated by 5 N/O atoms confirming the SC‐XRD results.^[^ ^19^ ^]^ This suggests that Cu in **Cat3** exists predominantly in the +2 oxidation state and is coordinated by phen ligands, consistent with our previous studies.^[^ ^16^ ^]^ EXAFS analysis (Figure 3f) further elucidates the local structural environment around Cu atoms in **Cat3**. Fitting of the Fourier‐transformed (FT‐)EXAFS data (Figure S42, Table S5, Supporting Information) for the as‐prepared powder reveals scattering contributions at distances of 1.94 ± 0.01 Å and 2.89 ± 0.02 Å with coordination numbers of 4.2 ± 0.5 for N/O and 2.2 ± 0.8 for Cu─O─Cu, respectively, in agreement with our single‐crystal diffraction data and DFT calculations (Figure S1 and Table S2, Supporting Information).

### 2.4 X‐Ray Absorption Spectroelectrochemistry

**Figure 4:**
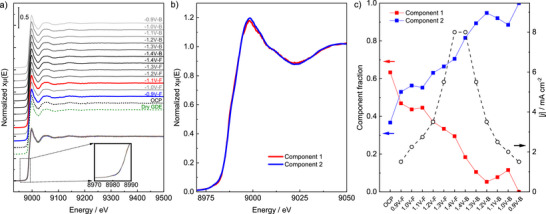
In situ XAS analysis of a **Cat3**‐modified electrode performed in a divided SEC batch cell. a) Cu K edge XANES spectra of the electrode in dry form and in aq. CO_2_‐saturated CsHCO_3_ electrolyte at OCP and various potentials between −0.9 and −1.4 V versus RHE. b) The two most distinct XANES spectra within the dataset from Figure 4a obtained via multivariate curve resolution‐alternating least squares (MCR‐ALS). c) Component fractions corresponding to the most distinct spectra (Figure 4b) obtained via MCR‐ALS, along with the corresponding current densities measured during in situ XAS analysis.

Despite the high *FE*_C2_, the stable performance over 36 h in the H‐cell configuration, and the good match between pre‐ and post‐electrolysis XANES/EXAFS spectra, the progression of the molecular framework of the organometallic Cu species during electrolysis yet remains uncertain. As known from the literature, reductive demetallation of Cu complexes with a negative bias can lead to the formation of CuO_x_ particles.^[^ ^20^ ^]^ In some cases, particle formation can even be reversible, i.e., the CuO_x_ particles transform back into the Cu coordination compounds when the bias is removed or when the potential returns to a more positive value.^[^ ^21^ ^]^ This in situ phase evolution warrants special attention, particularly in determining active sites. Consequently, XAS spectroelectrochemistry (SEC) was performed under eCO_2_RR conditions in a custom‐made divided cell in the three‐electrode configuration (**Figure** **4**; Figure S32–S43, Supporting Information).

XAS analysis was conducted in the dry state (catalyst‐sprayed carbon paper mounted in our in situ cell), in CO_2_‐saturated 0.1 m CsHCO_3_ solution at open‐circuit potential (OCP), and at potentials ranging from −0.9 to −1.4 V versus RHE. In situ XANES spectra showing the full energy range (8850 to 9500 eV) and the enlarged pre‐edge region (8970 to 8990 eV) are presented in Figure 4a. The results indicate that across the screened potential range, the Cu oxidation state and the d orbital structure remain nearly constant. Stability of the Cu coordination environment is corroborated by the EXAFS spectra (9030 to 9600 eV) and corresponding Fourier Transformed (FT‐EXAFS) profiles (Figure S33a, Supporting Information). A similar trend could be observed in control experiments (0.1 m KHCO_3_ under CO_2_ and 0.1 m CsHCO_3_ under Ar, displayed in Figures S35 and S36, Supporting Information). The dominant scattering path observed at near 1.9 Å corresponds to Cu interactions with light atoms, such as N/O, in the first coordination shell, whereas minimal Cu─Cu scattering paths appear at ≈2–4 Å, consistent with the oscillations observed in the k‐space profiles (Figure S33, Supporting Information). For a more detailed comparison, wavelet‐transformed EXAFS (WT‐EXAFS) analyses, based on both R‐space and k‐space profiles, were conducted for selected samples (Figure S34, Supporting Information), confirming the absence of inorganic Cu species and preservation of the local Cu coordination environment in as‐prepared **Cat3**.

For the **Cat3**‐modified electrode, the primary intensity maximum appears at ≈1.9 Å in R‐space and spans k‐values from 3 to 10 Å^−1^, corresponding to the first coordination shell while excluding the presence of reference materials such as CuO, CuCl_2_, and Cu_2_O. The intensity maximum in the WT‐EXAFS maps was maintained under negative bias, indicating the preservation of the local coordination environment around the Cu centers throughout the applied electrochemical potentials. Based on these findings, we conclude that the molecular scaffold exhibits structural stability up to −1.4 V versus RHE, which is maintained during the reverse potential scan to −0.9 V versus RHE.

To further elucidate the chemical behavior of Cu in **Cat3** under eCO_2_RR conditions, multivariate curve resolution–alternating least squares (MCR‐ALS) analysis was performed (Figure 4b,c). This analysis enables the deconvolution of overlapping spectral features to track subtle changes in the closer Cu coordination environment. For this purpose, the two spectra with the strongest variation, components 1 and 2, serve as internal references (Figure 4b). Notably, both profiles nearly overlap with each other, highlighting a negligible reaction‐induced shift in the Cu oxidation state and coordination environment under e‐CO_2_RR conditions. A transition to a smoother rising edge with a broader featureless white line is attributed to a very slight increase of the coordination number (Table S5, Supporting Information).^[^ ^19^, ^22^ ^]^ This effect is possibly be due to reductive cleavage of the Cu─Cl bonds at the terminal Cu centers (vide supra) as well as to coordination of water and/or CO_2_RR intermediates. The potential‐dependent fractions of the individual components in the spectra are shown in Figure 4c along with the corresponding current densities. Starting from the OCP, the relative fraction of component 2 steadily increases at the cost of component 1, however, since the spectral components are nearly identical, the absolute change in the Cu state is minimal. Despite this gradual change, the Cu centers in **Cat3** consistently remain in the Cu^2+^ state and exhibit nearly unchanged coordination environment at low current densities. This behavior highlights the stability of the molecular framework of **Cat3**, in contrast to the dynamic restructuring observed with Cu nanoparticles under identical conditions (Figure S37, Supporting Information).

### 2.5 MEA Electrolyzer Studies

**Figure 5:**
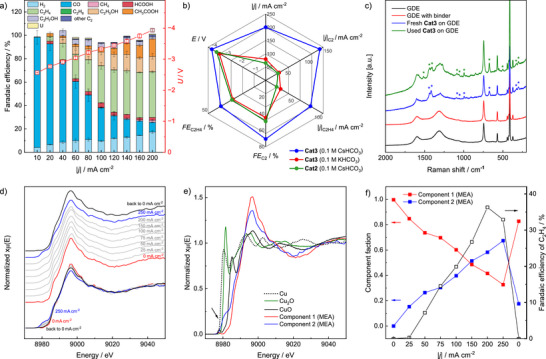
Electrochemical studies in an MEA electrolyzer using a **Cat3**‐modified GDE (0.1 m CsHCO_3_ as the anolyte). a) Relationship between current density, product distribution, and cell voltage. b) Comparison between the performance characteristics of **Cat3** and **Cat2**. c) Pre‐ and post‐electrolysis Raman spectra of **Cat3**‐modified electrode (excitation wavelength: 785 nm). Signals assigned to **Cat3** are marked with stars. d) Results of in situ Cu K‐edge high‐energy‐resolution fluorescence‐detected (HERFD) XANES analysis of a **Cat3**‐modified GDE under eCO_2_RR conditions at various current densities (aligning with the measurements in Figure 5a). e) Most distinct Cu K edge HERFD‐XANES spectra along with spectra of reference compounds. f) Component fractions corresponding to the most distinct Cu K edge HERFD‐XANES spectra (Figure 5e) obtained via MCR‐ALS analysis. *FE* values for C_2_H_4_ are inserted for comparison.

For testing the performance at high current density, **Cat3** was spray‐coated onto carbon paper gas diffusion electrodes (GDEs) and employed in a membrane electrode assembly (MEA) electrolyzer (Figures S44–S55, Supporting Information). While the cathode was fed with a stream of water‐saturated CO_2_, aq. 0.1 m CsHCO_3_ was pumped through the anodic half‐cell, with the applied current density ranging from 10 to 200 mA cm^−2^ (**Figure** **5**a). The cell voltage (without *IR* drop correction) increases from initially 2.5 to 4 V, which is somewhat higher than reported for metallic Cu catalysts within similar cell configurations.^[^ ^5^, ^23^ ^]^ At each step, the current was held constant for 15 min., followed by collection of the anolyte for liquid phase analysis by ^1^H NMR spectroscopy (for details of product analysis and calculations, see the SI). The major gas‐phase products are CO and C_2_H_4_, while the *FE* for H_2_ remains well below 20% across the entire current density range (Figures S46–S48, Supporting Information). Interestingly, the fraction of ethylene increases strongly with rising current density at the cost of CO formation, while the amount of CH_4_ formed remains negligible. C_2_H_5_OH and CH_3_COOH were the dominant liquid products, while the fractions of HCOOH and *n*‐PrOH each remain below 10%. As a result, the *FE* for all C_2_ products shows a steady increase with rising current density, with a maximum of 71% at 200 mA cm^−2^ (Figure S48, Supporting Information). This behavior differs from the results obtained in the H cell, where the fraction of C_2_ compounds ceases in favor of CH_4_ at elevated current densities (cf. Figure 2c). Furthermore, a control experiment using a GDE loaded with Cu nanoparticles shows a clearly different product distribution (see Figure S52, Supporting Information), once again highlighting the unique behavior of **Cat3**.

Analogous to the previous H‐cell studies, the choice of the cation significantly impacted the C─C coupling capability of **Cat3** under MEA conditions (Figure 5b). While a maximum *FE*_C2_ of 45% is already achieved at 80 mA cm^−2^ when using 0.1 m KHCO_3_ as the anolyte, a steady increase to 71% is observed at current densities up to 200 mA cm^−2^ when using 0.1 m CsHCO_3_ (for details, see Figures S46 and S47, Supporting Information). At 80 mA cm^−2^, *FE*_C2H4_ remained close to 35% for over 10 h, demonstrating robust and stable performance at continuous operation (Figures S53 and S54, Supporting Information).

Noteworthy, the binuclear Cu complex **Cat2** is significantly less stable in the MEA electrolyzer under the same conditions. Due to slow catalyst leaching, this system cannot be used effectively for a longer period, which is why insoluble **Cat3** offers a clear advantage in the MEA environment. In view of C_2_H_4_ production rate, **Cat3** exhibits a steadily increasing turnover frequency (*TOF*) with rising current density, reaching 178 h^−1^ at 200 mA cm^−2^. In contrast, *TOF(*C_2_H_4_) decreases for **Cat2** when the current density rises above 50 mA cm^−2^ (for details, see Figure S55, Supporting Information).

After disassembling the post‐electrolysis MEA, the catalyst layer was examined and subjected to SEM, Raman spectroscopy, and TEM (Figures S56–S63, Supporting Information). Identical to the as‐sprayed GDE, the coating retained its greenish color (Figure S56, Supporting Information). SEM‐EDX revealed a layer morphology similar to that of freshly prepared electrodes, whereby Cu remains evenly distributed across the surface (Figures S57–S61, Supporting Information). The Raman spectra of the fresh and the used **Cat3**‐modified electrode exhibit the same characteristic bands at 302, 327, 674, 910, 995, 1096, 1304, 1418, 1438, and 1602 cm^−1^ (Figure 5c). Additional bands at 374, 410, 441, 570, and 743 cm^−1^ stem from the used sapphire probe and those at 1314 and 1599 cm^−1^ from the carbon paper. The broad bands around ≈530 and 620 cm^−1^, typical for Cu/CuO_x_ nanoparticles, (Figure S64, Supporting Information) are neither contained in the spectrum of the fresh nor the used electrode.

To study the structure of **Cat3** under operational conditions, in situ HERFD‐XANES was carried out using a custom‐made MEA‐type spectroelectrochemical cell (Figure S65, Supporting Information). The HERFD‐XANES technique with its exceptionally high energy resolution^[^ ^24^ ^]^ allows to highlight even small spectral changes related to the **Cat3** framework. The applied current density was stepwise increased from 0 mA cm^−2^ (OCV) to 250 mA cm^−2^. Despite nearly constant spectral features from 8990 to 9500 eV across all current densities (Figure S66, Supporting Information), a slight but gradual increase in the pre‐edge intensity at 8980 eV was observed (Figure 5d; Figure S67, Supporting Information). This growing pre‐edge feature, associated with the formally forbidden 1s to 3d transition, could be tentatively attributed to structural distortion (via 3d–4p orbital mixing), weakening of metal–ligand bonds,^[^ ^25^ ^]^ or formation of inorganic species.^[^ ^26^ ^]^

To further disentangle the hybrid/mixed phases present during the reaction, MCR‐ALS analysis was applied to the MEA‐derived XAS dataset and extracted two distinct spectral components, as shown in Figure 5e. Component 1 closely resembles the initial state of the catalyst, whereas component 2 represents the mixture with the highest concentration of the second phase emerging under electrochemical conditions. In component 2, Cu remains coordinated to two N/O ligands, and a Cu─Cu distance of ≈2.5 Å with a coordination number of 2 is observed. Approximately 35% of the Cu is estimated to be in a reduced state (Figure S68, Table S6, Supporting Information). However, the absence of a significant K‐edge shift to lower energies, along with a low average Cu─Cu coordination number, and in combination with higher white line intensity comparable to standard references of Cu, Cu_2_O, and CuO (Figure 5e), suggests minimal formation of inorganic Cu‐based particles.

At a current density of 200 mA cm^−2^, the sample exhibits nearly 60% component 2 (Figure 5f), comprising 35% reduced Cu moieties. This outcome corresponds to about a 20% overall reduction, and 80% of the Cu remains in the Cu^2+^ state. These observations are consistent with our linear combination analysis (LCA), where Cu^0^ is used to represent the reduced state (Figure S69, Table S7, Supporting Information). Moving to 250 mA cm^−2^, the contribution of component 2 progressively increased, reaching ≈70% (24.5% reduced Cu), however, causing a degradation of C_2_H_4_ production (Figure 5f). Interestingly, upon removal of the applied bias, a partial recovery of the Cu electronic state can be observed, both in the pre‐edge region and the extended fine structure, indicating a degree of reversibility in the initial local environment of the Cu centers. Notably, K‐edge profiles recorded at 200 mA cm^−2^ (Figure S70, Supporting Information), averaged over 4‐min intervals (corresponding to the maximum *FE*_C2H4_ yield, see Figure 5b), suggest that this structural change is potential/current‐dependent, but time‐independent.

## 3 Conclusion

Our study provides new insights into the catalysis of electrochemical CO_2_ reduction with molecular Cu phenanthroline compounds. Screening of a series of differently substituted phenanthrolines revealed 4,7‐dimethoxy‐1,10‐phenathroline to be the most effective ligand. The resulting water‐insoluble **Cat3** is a cascade‐like coordination oligomer that features *µ*_2_‐ and *µ*_3_‐hydroxo‐bridged Cu centers with relatively short Cu···Cu distances. The material is readily synthesized using inexpensive building blocks and a straightforward protocol. Compared to the previously reported binuclear Cu phenanthroline compound **Cat2**, the stability of **Cat3** under electrocatalytic conditions is significantly enhanced, allowing for operation in an MEA electrolyzer at high current densities. When spray‐coated onto a carbon gas diffusion electrode, **Cat3** achieves more than 70% *FE*_C2+_ at 200 mA cm^−2^, while stable performance is observed for 10 h with no obvious performance degradation. Pre‐ and post‐electrolysis analysis by SEM and Raman spectroscopy reveal that the electrode is coated with a stable composite matrix containing homogeneously dispersed copper and no obvious formation of inorganic Cu species.

In many previous works, the decomposition of molecular Cu catalysts during eCO_2_RR is reported, resulting in the formation of Cu/CuO_x_ particles that serve as the true active species. Conclusions regarding the type of active site should therefore be drawn with great caution and must be supported by in situ spectroscopic data. To monitor the structural evolution of **Cat3** under reaction conditions, in situ XAS was carried out under potential control in the kinetic regime using a liquid flow cell, and under galvanostatic conditions at high current densities in an MEA‐type cell. While in the kinetic regime, no significant changes of the spectral features were observed, the results obtained with the MEA‐SEC cell suggest gradual structural changes when increasing the current density from 50 to 250 mA cm^−2^, i.e., reduction of the Cu oxidation state, decrease of the Cu···Cu distance, and a lowering of the Cu─N/O coordination number. However, in view of the still low Cu─Cu coordination number, absence of K‐edge shift to lower energies, and high white line intensities compared to inorganic Cu reference species, no or only minimal formation of Cu/CuO_x_ particles is assumed. Strikingly, the original phase is largely restored upon bias removal, highlighting both the structural robustness and adaptive redox behavior of **Cat3** under harsh electrolysis conditions. While future studies will address the potential‐dependent structural modifications as well as the eCO_2_RR mechanism in detail, the present work highlights a possible pathway toward implementing molecular electrocatalysts in CO_2_ electrolyzers for the synthesis of C_2+_ products.

## 4 Experimental Section

### Chemicals

Copper chloride (CuCl_2_, 99%), cesium bicarbonate (CsHCO_3_, 99.99%), methanol (99.8%), ethanol (99.8%), and isopropanol (99.8%) were purchased from Fisher Scientific. 4,7‐Dimethoxy‐1,10‐phenanthroline (97%) was purchased from BLD Pharmatech Ltd. (China). Triethylamine (99.5%), potassium bicarbonate (99.99%), dimethyl sulfoxide (DMSO, 99.9%), copper nanoparticles (Cu NPs, 40–60 nm, 99.5%), and Nafion 1100W solution were purchased from Sigma‐Aldrich (Germany). Anode electrode (commercial IrO_x_‐coated gas diffusion electrode (GDE)) and Sustainion XA‐9 alkaline ionomer powder, both for use in the MEA electrolyzer, were purchased from Dioxide Materials (US). Toray Carbon Paper 060 (wet‐proofed), carbon gas diffusion layers (GDL, Freudenberg H23C8), and PiperION anion exchange membranes (20 microns) were obtained from The Fuel Cell Store (US). Deuterium oxide (D_2_O, 99.9%) for NMR measurements was purchased from Deutero GmbH (Germany). CO_2_ (99.9999%) and Ar (99.9999%) were obtained from Linde AG (Germany).

### Synthesis of **Cat3**

The catalyst was synthesized according to the reported method in the previous work.^[^ ^16^ ^]^ A solution of 134.45 mg (1 mmol) CuCl_2_ in 1 mL ultrapure water was combined with a solution of 240.60 mg (1 mmol) 4,7‐dimethoxy‐1,10‐phenanthroline in 4 mL of ethanol under stirring at room temperature, resulting in the formation of a light green precipitate. After stirring for 10 min, 10 mL triethylamine was added to the suspension, leading to an immediate change of the precipitate color to dark green. The mixture was stirred for an additional 10 min. The precipitate was then filtered off, washed with ultrapure water, and dried overnight under vacuum at 60 °C, yielding a crystalline dark green product (yield: 300 mg). Elemental analysis: Cu 18.10, C 46.02, H 3.55, N 7.39, Cl 7.84.

Attempts to prepare high‐quality single crystals of **Cat3** for SC‐XRD analysis using the previously described crystallization procedure^[^ ^16^ ^]^ remained unsuccessful. Instead, an alternative procedure was applied, wherein **Cat3** powder (60 mg) was partially dissolved in MeOH, followed by heating to 85 °C for 12h in an autoclave. Afterward, the mixture was allowed to cool to room temperature. Crystallization proceeded within four days. The obtained crystals were subjected to SC‐XRD (see Figure S1, Supporting Information).

To confirm the identity of **Cat3** crystals, HERFD XAS (Figure S3, Supporting Information), P‐XRD (Figure S8, Supporting Information), and Raman spectroscopy (Figure S9, Supporting Information) were performed. In each case, a good agreement was observed between the results of **Cat3** powder and crystalline **Cat3**.

### Materials Characterization

X‐ray diffraction (XRD) data were obtained using a Panalytical X'Pert Pro diffractometer with Cu‐Kα radiation, while single‐crystal XRD (SC‐XRD) was carried out on a Kappa APEX II Duo diffractometer from Bruker AXS. The copper content of **Cat3** was analyzed using inductively coupled plasma optical emission spectrometry (ICP‐OES) with an Agilent 715‐ES spectrometer. Carbon, hydrogen, and nitrogen contents were determined via combustion analysis using a TruSpec Micro elemental analyzer (LECO Instrumente GmbH, Germany). The morphology of the electrode surface, both before and after electrolysis, was examined using scanning electron microscopy (SEM) with a MERLIN VP Compact (Zeiss, Germany) equipped with EDX. ^1^H nuclear magnetic resonance (NMR) spectroscopy was performed with a Bruker AVANCE 500 NEO spectrometer.

For scanning electron microscopy (SEM, field emission), the samples were mounted on a heavy metal‐free Al‐SEM‐carrier (co. PLANO, Wetzlar, Germany) with adhesive conductive carbon tape (Spectro Tabs, TED PELLA INC, Redding, USA) and coated with carbon (5.0 nm thickness) under vacuum (CCU 010 HV‐Coating Unit, Co. Safematic GmbH, Zizers, Switzerland). Analysis was carried out using a field emission scanning electron microscope (SEM, MERLIN VP Compact, Co. Zeiss, Oberkochen, Germany) equipped with an energy‐dispersive X‐ray (EDX) detector (XFlash 6/30, Co. Bruker, Berlin, Germany). Representative areas of the samples were analyzed and mapped for elemental distribution based on the EDX‐spectra data by QUANTAX ESPRIT Microanalysis software (version 2.0). SEM‐images were taken from the selected regions.

Scanning Transmission Electron Microscopy (STEM) was performed on a probe aberration‐corrected JEM‐ARM200F (JEOL, corrector: CEOS) at an acceleration voltage of 200 kV. A High‐Angle Annular Dark Field (HAADF) and an Annular Bright Field (ABF) detector were used for imaging. The solid samples were prepared as described in the Supporting Information and deposited without any pre‐treatment onto a carbon‐supported Ni grid (mesh 300) and finally transferred to the microscope.

XPS (X‐ray Photoelectron Spectroscopy) measurements were performed on an ESCALAB 220iXL (Thermo Fisher Scientific) with monochromated Al Kα radiation (E = 1486.6 eV). Samples are prepared on a stainless‐steel holder with conductive double‐sided adhesive carbon tape. The measurements are performed with charge compensation using a flood electron system that combines low‐energy electrons and Ar^+^ ions (p_Ar_  = 1 × 10^−7^ mbar). The electron binding energies are referenced to the C 1s core level of carbon at 284.8 eV (C─C and C─H bonds). For quantitative analysis, the peaks were deconvoluted with Gaussian–Lorentzian curves using the software Unifit 2023. The peak areas were normalized by the transmission function of the spectrometer and the element‐specific sensitivity factor of Scofield.

Raman spectra of **Cat3**‐modified electrodes (before/after electrolysis) were measured using a Renishaw inVia Raman microscope equipped with a 633 nm air‐cooled He‐Ne laser. For the measurement, the electrode or a spatula tip of the powdered sample was placed on a microscope slide, and the laser beam was focused on the sample using a ×20 objective. The Laser power was maintained at a relatively low level, ranging from 0.085 to 0.17 mW, to avoid damaging effects that may otherwise occur. The exposure time of a spectrum was 30 s with 3 accumulations. To obtain a broader cross‐section of the sample, a selection of samples was analyzed using a Horiba iHR 550 Raman spectrometer equipped with a 785 nm laser source (Oxxius, maximum laser power: 864 mW) connected to a fiber optical ball probe with a sapphire lens (MarqMetrix, 3/8″ Process BallProbe). The samples were measured in the direct contact mode. The measurement parameters varied depending on the sample: exposure time 10–30 s, 3 accumulations.

### Electrode Preparation

For measurements in an H‐cell, the electrode was prepared according to the previous work.^[^ ^16^ ^]^ The catalyst (6 mg) was suspended in a mixture containing 450 µL of ultrapure water, 90 µL of isopropanol, and 60 µL of Nafion solution, then ultrasonicated for 15 min to produce a homogeneous ink. Next, 50 µL of the ink was applied to a strip of carbon paper (1 cm wide) by drop‐casting, covering an area of 1 cm^2^ on both sides (resulting in 2 cm^2^ active area in total). The electrode was then allowed to dry in air. The catalyst loading (**Cat3**) for each working electrode was 0.5 mg cm^−2^.

For measurements in a membrane electrode assembly (MEA) electrolyzer, 40 mg of catalyst was suspended in a mixture of 3 mL ultrapure water, 600 µL isopropanol, and 400 µL Sustainion XA‐9 alkaline ionomer solution (5 wt.% ionomer in EtOH, Dioxide Materials). The mixture was ultrasonicated to generate a homogeneous ink. Then, the ink was distributed homogeneously on the micro‐porous layer of the carbon paper (Freudenberg C8H23 GDL, 25 cm^2^ geometrical surface area) by spray coating at 60 °C, followed by air‐drying for 10 min. The GDL was weighed before and after the spray‐coating, and the mass difference was kept near 35 mg (on 25 cm^2^ electrode), resulting in a catalyst loading of 1 mg cm^−2^ and ≈30 wt.% ionomer content in the dry as‐prepared catalyst layer. From the coated GDL, pieces of 5 cm^2^ were cut out and used as cathode for MEA measurements.

### Electrochemical Experiments

Linear sweep voltammetry (LSV), controlled potential electrolysis (CPE), and controlled current electrolysis (CCE) were performed using a VIONIC potentiostat from Metrohm (Germany). CPE was carried out in an H‐cell (separator: glass frit with pore size G4). A platinum plate and a Ag/AgCl electrode (3 m KCl solution) served as a counter and a reference electrode, respectively. A 0.1 m CsHCO_3_ solution (pH 6.8 under CO_2_, pH 8.9 under Ar) served as the electrolyte. Prior to each CPE experiment, CO_2_ was bubbled at a flow rate of 40 mL min^−1^ for 30 min. Current densities were calculated based on the total immersed electrode surface area of 2 cm^2^. Potentials measured vs. Ag/AgCl were converted to the reversible hydrogen electrode (RHE) using the following relationship.^[^ ^27^ ^]^

(1) $$E\;\left(\mathrm{vs}.\;\mathrm{RHE}\right)=E\left(\mathrm{vs}.\;\mathrm{Ag}/\mathrm{AgCl}\right)+0.197\;V+0.0591\cdot pH$$

The MEA measurements were performed at room temperature. The spray‐coated GDE was used as working electrode for eCO_2_RR, and a commercial IrO_x_‐based electrode (from Dioxide Materials) served as the counter electrode. The cathode and anode chambers were separated using a PiperION anion exchange membrane (thickness: 20 µm). Humidified CO_2_ was purged through the cathode chamber at a flow rate of 50 sccm (room temperature), while a N_2_ bleeding line (flow rate: 20 sccm) was injected after the electrolyzer, serving as the flow rate internal standard. 0.1 m CsHCO_3_ solution was circulated through the anode chamber as anolyte with a flow rate of 30 sccm, using a Simdos 10 diaphragm pump.

### Product Quantification

Gaseous products from CPE (H‐cell) and CCE (MEA set‐up) were analyzed using gas chromatography (GC, Agilent 6890) with argon as the carrier gas, calibrated using certified gas mixtures from Air Liquide as standards. A thermal conductivity detector (TCD) was used to analyze H_2_ and CO, while a flame ionization detector (FID) was employed to quantify CH_4_, C_2_H_4_, and C_2_H_6_. For CPE and CCE, cells were connected to the GC for online analysis by using a steady flow of CO_2_ gas of 20 mL min^−1^ (H‐cell) and 50 mL min^−1^ (MEA set‐up), respectively. In the meantime, N_2_ bleeding with a flow rate of 22 mL min^−1^ served both as an internal standard and to compensate for the CO_2_ that was consumed during the reaction (for details, see Figure S44, Supporting Information). The Faradaic efficiencies (*FE*) for specific gaseous products *x* (e.g., *x*  =  C_2_H_4_) were calculated using the following equation,^[^ ^28^ ^]^

(2) $$FE_{x}\left[\%\right]=\frac{p\overset{\cdot }{V}\phi_{x}zF}{RTi}\cdot 100$$

where $\dot{V}$ refers to the flow rate of the gas delivered to the gas chromatograph at a specified sampling time with N_2_ compensation, ϕ_*x*_ to the volume fraction of the gaseous product (e.g., C_2_H_4_) in the gas flow that was delivered to the detector, *p* to the pressure (1.01 × 10^5^ Pa), *R* to the gas constant, *T* to the temperature (296 K), *z* to the number of electrons transferred for CO_2_‐to‐*x* (i.e., *z*  =  12 for C_2_H_4_), *F* to the Faraday constant, and *i* to the current recorded by the potentiostat.

The liquid products were quantified by ^1^H NMR spectroscopy using a water pre‐saturation method and DMSO as an internal standard. For sample preparation, a 500 µL aliquot of the post‐electrolysis electrolyte solution was mixed with 100 µL D_2_O and 35 µL DMSO. *FE*_x_ was calculated using the following relationship,

(3) $$FE_{x}\left[\%\right]=\frac{c_{x}VzF}{Q}\cdot 100$$

wherein *c_x_* denotes the concentration of the product (e.g. formate) in the liquid phase, *V* represents the catholyte volume (17 mL), and *q* is the total amount of passed electric charge at the specified sampling time.

### Ex Situ and In Situ X‐Ray Absorption Spectroscopy (XAS) Measurements

All ex situ measurements for powder and electrode were performed in fluorescence mode. All in situ XAS measurements were carried out using either a divided spectroelectrochemical (SEC) liquid flow cell (three‐electrode arrangement) or a SEC MEA cell (galvanostatic conditions) in the fluorescence mode.

For measurements in the SEC flow cell, electrode preparation follows the same procedure as in the H‐cell studies (catalyst loading: 1 mg cm^−2^ with 15 wt.% Nafion ionomer, deposited on Freudenberg C2 carbon paper). The anodic and cathodic half‐cells were separated by a Nafion 117 membrane. An Ag/AgCl reference electrode was placed in the cathode chamber for control of the working electrode potential. All in situ XAS measurements were carried out at the near (XANES) and extended (EXAFS) Cu K‐edge at the XPP KMC‐3 beamline (BESSY II). The employed flow cell is shown in Figure S32 (Supporting Information). The X‐ray beam was focused to a spot size of 500 × 500 µm at a fixed sample position. The beam energy was scanned from 8900 to 9600 eV (scan rate: 1.6 eV s^−1^). An aqueous 0.1 m CsHCO_3_ solution was used as both catholyte and anolyte and pumped through the half‐cells at a flow rate of 20 mL min^−1^. The catholyte was purged with CO_2_ for 30 min prior to each measurement and feeding to the catholyte was continued to ensure CO_2_ saturation. For every measurement at a defined potential, two in situ XAS spectra were collected. Data reduction and processing of the XANES spectra, including background subtraction, energy correction, normalization, and linear combination fitting were performed using the standard Athena software from the Demeter 0.9.26 program package.

Measurements in the SEC MEA cell (Figure S65, Supporting Information) were performed in the High‐Energy‐Resolution Fluorescence‐Detected XAS (HERFD‐XAS) mode at the ID26 beamline of the European Synchrotron Radiation Facility (ESRF, Grenoble, France). The higher harmonics were suppressed by Si‐coated mirrors which operate in total reflection mode. The desired energy was selected by a Si(111) double crystal monochromator. The fluorescence X‐rays were collected by an X‐ray spectrometer using the (800) reflection of two spherically bent Ge crystals, and the photons were counted by a PILATUS3 100K‐M detector. The monochromator energy calibration was performed by measuring Cu foil. For the HERFD‐XANES measurements the monochromator energy was scanned while the spectrometer was kept fixed at the maximum of the Kβ1,3 emission line (8903.6 eV). The assembly of the MEA SEC was carried out analogously to MEA performance characterization described above (catalyst loading: 1 mg cm^−2^ with 15 wt.% Sustanion XA‐9 ionomer, sprayed on Freudenberg C2 carbon paper). The beam energy was scanned from 8900 to 9600 eV (scan rate: 11.6 eV s^−1^). Characterization of the SEC MEA cell including analysis of the product mixture was carried out separately using the GC quantification protocol detailed above, whereby good agreement with the standard MEA electrolyzer was found. Data reduction and modelling of the EXAFS spectra were conducted using the standard Athena and Artemis software from the Demeter 0.9.26 program package. EXAFS data were analyzed in the R‐space using fixed k and R ranges (2.0–11.0 Å^−1^, R: 1.0–3.0 Å). Wavelet transformations of EXAFS (WT‐EXAFS) spectra were calculated using the Hama Fortran program.^[^ ^29^ ^]^ The Morlet wavelet was used for the analysis. The raw k3 weighted data were added to the Hama_Fortran program for analysis. For the Multivariate Curve Resolution–Alternating Least Squares (MCR‐ALS) the spectral datasets corresponding to each measurement were imported and normalized using the FASTOSH v. 1.0.8 software.^[^ ^30^ ^]^ To derive the pure spectra and the respective relative concentrations, the MCR‐ALS Matlab toolbox^[^ ^31^ ^]^ integrated into FASTOSH was used.

### Statistical Analysis

All statistical analyses in this work were performed using the Origin 2023 software. Before data analysis, irrelevant data detection and elimination were performed on the data sets. Experimental errors for FE and average │*j│* values were calculated as the standard error of the mean using a sample size of *n* = 3. Experimental data in bar diagrams were presented as mean values ± standard deviation.

## Conflict of Interest

The authors declare no conflict of interest.

## Supporting information



Supporting Information

## Data Availability

The data that support the findings of this study are available in the supplementary material of this article.
