# Supplementary material for: Heterogenized Copper(II) Phenanthroline Catalysts for Electroreduction of CO2 to C2 Compounds: Substitution on the Ligand Causes Structural Changes to the Molecular Framework and Stability Enhancement
Source: Adv Mater. 2025 Oct 1;38(3):e13702. doi: 10.1002/adma.202513702 (PMC12801368; doi:10.1002/adma.202513702)
Supplement: Supplementary file 1 — Supporting Information [file ADMA-38-e13702-s001.pdf]

ADVANCED MATERIALS

Supporting Information

for *Adv. Mater.*, DOI 10.1002/adma.202513702

Heterogenized Copper(II) Phenanthroline Catalysts for Electroreduction of CO_2 to C_2 Compounds: Substitution on the Ligand Causes Structural Changes to the Molecular Framework and Stability Enhancement

Na Liu, Trang Minh Pham, Yanan Han, Linfeng Yang, Olga S. Bokareva, Stephan Bartling, Armin Springer, Anke Spannenberg, Christoph Kubis, Jana Weiss, Dmitry E. Doronkin, Wen Ju and Robert Francke**

Heterogenized Copper(II) Phenanthroline Catalysts for Electroreduction of CO_2 to C_2 compounds: Substitution on the Ligand Causes Structural Changes to the Molecular Framework and Stability Enhancement

Na Liu,[a] Trang Minh Pham,[a] Yanan Han,[a] Linfeng Yang,[a] Olga S. Bokareva,[a,b] Stephan Bartling,[a] Armin Springer,[c] Anke Spannenberg,[a] Christoph Kubis,[a] Jana Weiss,[a] Dmitry E. Doronkin,[d] Wen Ju,*,[a] Robert Francke*,[a]

[a] Leibniz Institute for Catalysis, Rostock 18059, Germany.

[b] Institute of Chemistry and Department of Life, Light and Matter, University of Rostock, 18059 Rostock, Germany.

[c] Electron Microscopy Center, University Medicine Rostock, Strempelstr. 14, 18057 Rostock, Germany.

[d] Institute for Chemical Technology and Polymer Chemistry and Institute of Catalysis Research and Technology, Karlsruhe Institute of Technology, Karlsruhe, Germany.

Content

1. General information 2
2. Structural analysis of as-prepared **Cat3** 3
3. Behavior of **Cat3** in liquid CO_2-saturated electrolyte 14
   3.1. Electrochemical studies in H-cells 14
   3.2. Post-electrolysis characterization of Cat3-modified carbon electrodes 22
   3.3. *In situ* XAS analysis under potential control using a divided flow cell 27
4. Characterization of **Cat3** in the MEA environment 32
   4.1. Electrochemical studies in a MEA electrolyzer 32
   4.2. Pre- and post-electrolysis characterization of **Cat3**-modified GDEs 38
   4.3. *In situ* XAS analysis using a SEC MEA cell 44

1. General information

All information on experimental procedures and employed materials are provided in the experimental section of the manuscript. All DFT calculations were performed in gas phase using ORCA 5.0.3.[1] The BP86[2] functional in combination with the def2-SVP[3] basis set was employed, together with the RI approximation, D3 dispersion correction,[4] and VeryTightSCF convergence criteria for the optimization of dimeric and tetrameric models. For the more computationally demanding octamer structure, optimization was performed using TightOpt and LooseSCF criteria, with additional damping enabled via the SlowConv keyword to ensure convergence. To speed up DFT calculations, the initial conformer search for the octamer structure was done with the xTB semi-empirical method.[5] The optimized structures of both dimer and tetramer models were confirmed to exhibit no imaginary frequencies, ensuring that true local minima were obtained. Given the presence of transition metal (copper) atoms, relative stability analysis using VeryTightSCF keyword was conducted to determine the ground-state multiplicity (see Table S1). For the dimer and tetramer, the most stable multiplicities before and after protonation are 3 and 5, respectively. For the octamer, the most stable multiplicity before protonation is 5 and after protonation is 3. In the optimized structures of the tetramer and octamer, the side ligands are not co-planar. However, this distortion may result from the simplified gas-phase model, which lacks the realistic crystal environment.

[1] F. Neese, *WIREs Comput. Mol. Sci.* **2022**, *12*, e1606.
[2] A. D. Becke, *Phys. Rev. A* **1988**, *38*, 3098-3100.
[3] F. Weigend, R. Ahlrichs, *Phys. Chem. Chem. Phys.* **2005**, *7*, 3297-3305.
[4] a) S. Grimme, J. Antony, S. Ehrlich, H. Krieg, *J. Chem. Phys.* **2010**, *132*, 154104. b) S. Grimme, S. Ehrlich, L. Goerigk, *J. Comput. Chem.* **2011**, *32*, 1456-1465.
[5] C. Bannwarth, S. Ehlert, S. Grimme, *J. Chem. Theory Comput.* **2019**, *15*, 1652-1671.

2. Structural analysis of as-prepared Cat3

SC-XRD analysis of the crystal prepared by crystallization in the autoclaves (see Experimental Section) confirms a cascade-like core structure composed of four μ-O-bridged dimeric Cu···Cu subunits coordinated with phenanthroline ligands. Two of the terminal Cu atoms are coordinated with chloro ligands, as shown in Figure S1. No information about the presence of hydrogen at the bridging oxygen atoms and the content in the remaining voids of the crystal packing (counter ions and solvent molecules) is available due to the insufficient quality of the crystals. To gain a more precise understanding of the catalyst structure, we conducted XAS (Figure S2 and Figure S3), XPS (O1s, Figure S4) and DFT calculations (Figure S5) to strengthen the hypothesized structure and composition of the as-prepared catalyst.

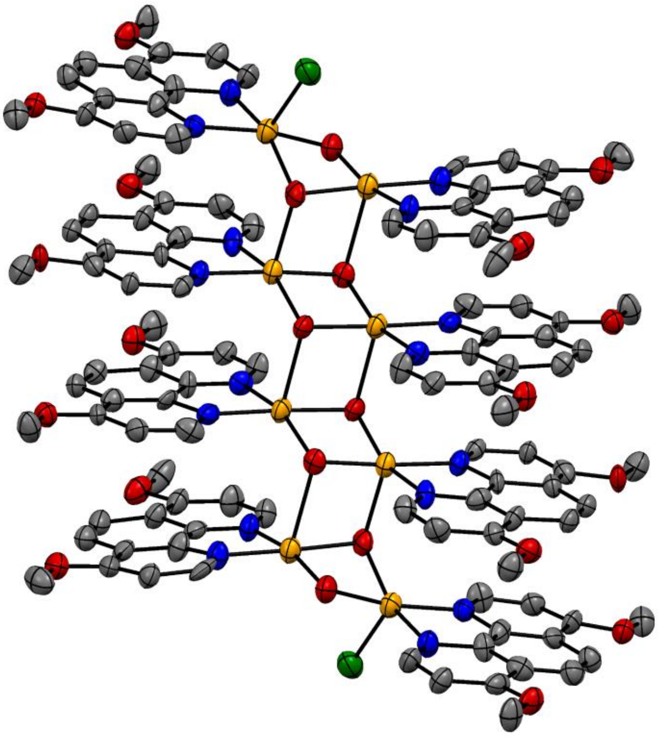

Figure S1. The core cascade-like unit of **Cat3** determined by SC-XRD (displacement ellipsoids correspond to 30% probability, crystal system: triclinic, space group $P\overline{1}$, a = 14.0849(13), b = 15.4891(13), c = 16.7412(14) Å, α = 76.091(6), β = 71.867(7), γ = 75.482(7)°).

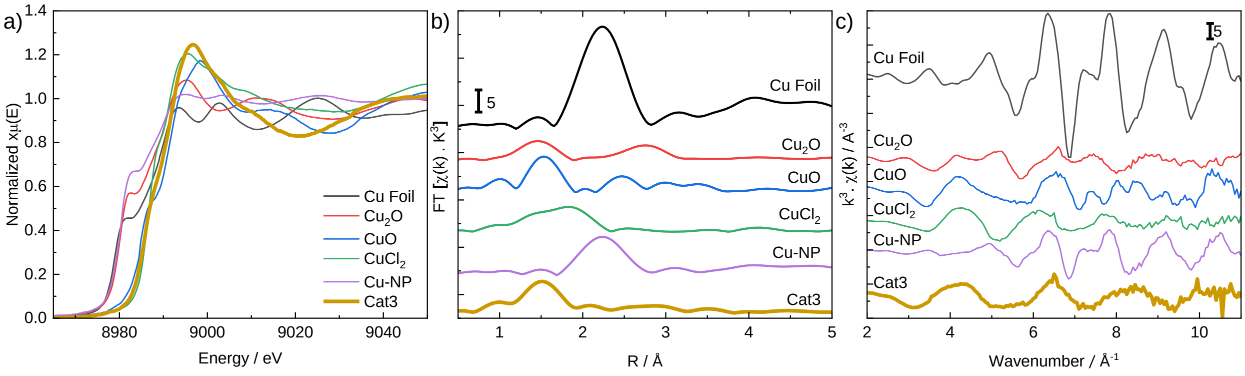

Figure S2. a) Normalized Cu K-edge XANES spectra, b) Fourier transform EXAFS spectra and c) k-space spectra of Cu-based samples including as-prepared **Cat3** powder.

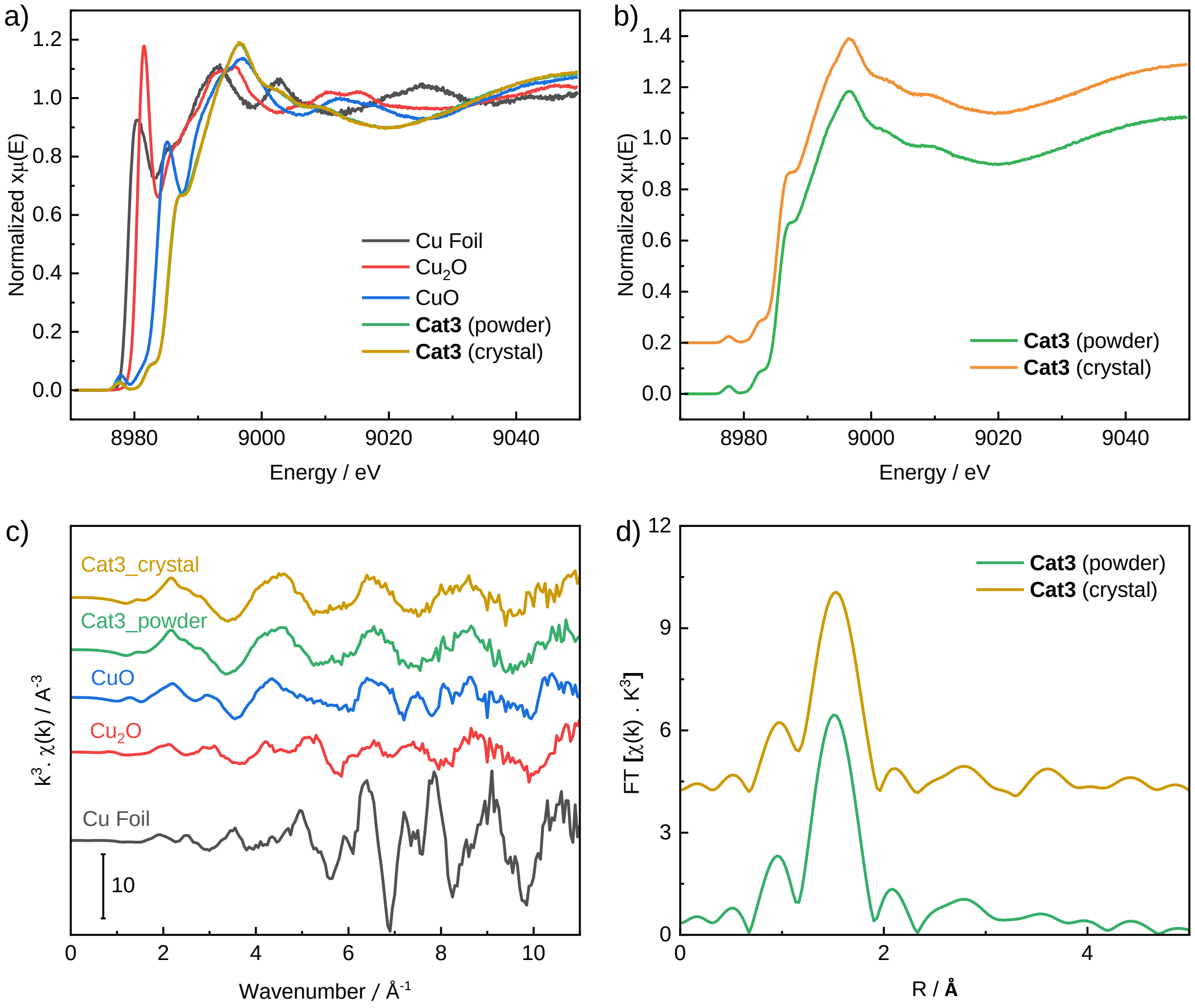

Figure S3. a,b) Normalized Cu K-edge HERFD-XANES spectra, c) k-space spectra, and d) Fourier transform EXAFS spectra of as-prepared **Cat3** powder and **Cat3** crystals obtained from recrystallization using the autoclave procedure described in the Experimental Section.

In Figure S4, the XPS of the O 1s region for **Cat3** is shown and compared to the spectrum of **Cat2**, the latter being coordinated by the unsubstituted 1,10-phenanthroline ligand (see Figure 1a). While **Cat2** exhibits a single main peak at 530.6 eV that can be assigned to the bridging μ-OH unit,[6] **Cat3** exhibits two signals, one at the same binding energy (530.6 eV) and one at 533.2 eV, suggesting the presence of μ-OH bridges and O-CH_3 groups (from the 4,7-dimethoxy-1,10-phenanthroline ligand), respectively. This result clearly indicates that the bridging oxygen atoms are protonated.

[6] N. Liu, S. Bartling, A. Springer, C. Kubis, O. S. Bokareva, E. Salaya, J. Sun, Z. Zhang, S. Wohlrab, A. M. Abdel-Mageed, H. Q. Liang, R. Francke, *Adv. Mater.* **2024**, *36*, 2309526.

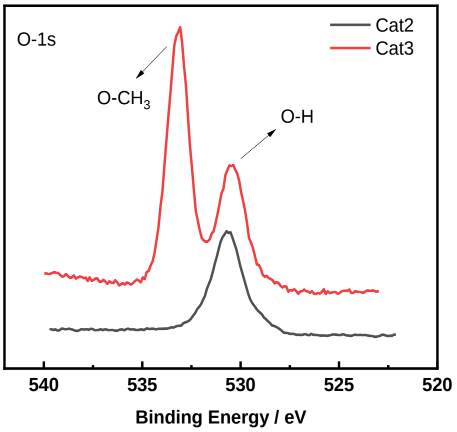

Figure S4. XPS analysis of the O 1s region for **Cat2** and **Cat3** (both catalysts were analyzed in powder form).

The octameric framework obtained from SC-XRD data (Figure S1) was selected as the initial guess for DFT calculations to determine the most probable protonation sites (Figure S5e and f). Due to the computational cost of simulating the entire octamer catalyst, additional dimeric (Figure S5a and b) and tetrameric (Figure S5c and d) models were constructed for reference and comparison.

To investigate the impact of protonation of the oxo bridge on the geometry and electronic structure of **Cat3**, spin density was compared for both protonated and non-protonated forms of all three oligomeric species. Protonation induces significant non-planarization of the framework and alters spin density localization (see Figure S5). It preferentially occurs at bridging oxygen sites, leading to a chemically reasonable model with eight protons for the octamer. This finding supports the hypothesis that local protonation events drive overall electronic stabilization.

In both tetrameric and octameric species, *O*-protonation results in H-bonding along the cascade-like core structure, likely leading to further stabilization of the oligomers. The average O-H bond distance is 0.997 Å (1.002 Å for tetramer and 0.995 Å for octamer), while the average O···H bond distance is 1.612 Å (1.504 Å for tetramer and 1.648 Å for octamer). Across all species, spin density on bridging oxygen atoms decreases, with the octamer **Cat3** (Figure S5f) exhibiting coexisting α- and β-spin densities on the same oxygen atoms, suggesting a more stabilized electronic configuration upon protonation. The redistribution of unpaired electrons may influence the magnetic and redox properties of the system. Given the consistency of these trends across all species, we conclude that model oligomers provide reliable approximations for understanding local electronic changes in the larger octamer framework.

Due to the complexity of the octamer and high computational cost, only gas-phase simulations were conducted in this study. More sophisticated solvent models, including other molecules and counterions present in the system, will be explored in future work.

a) b) c) d) e) f)

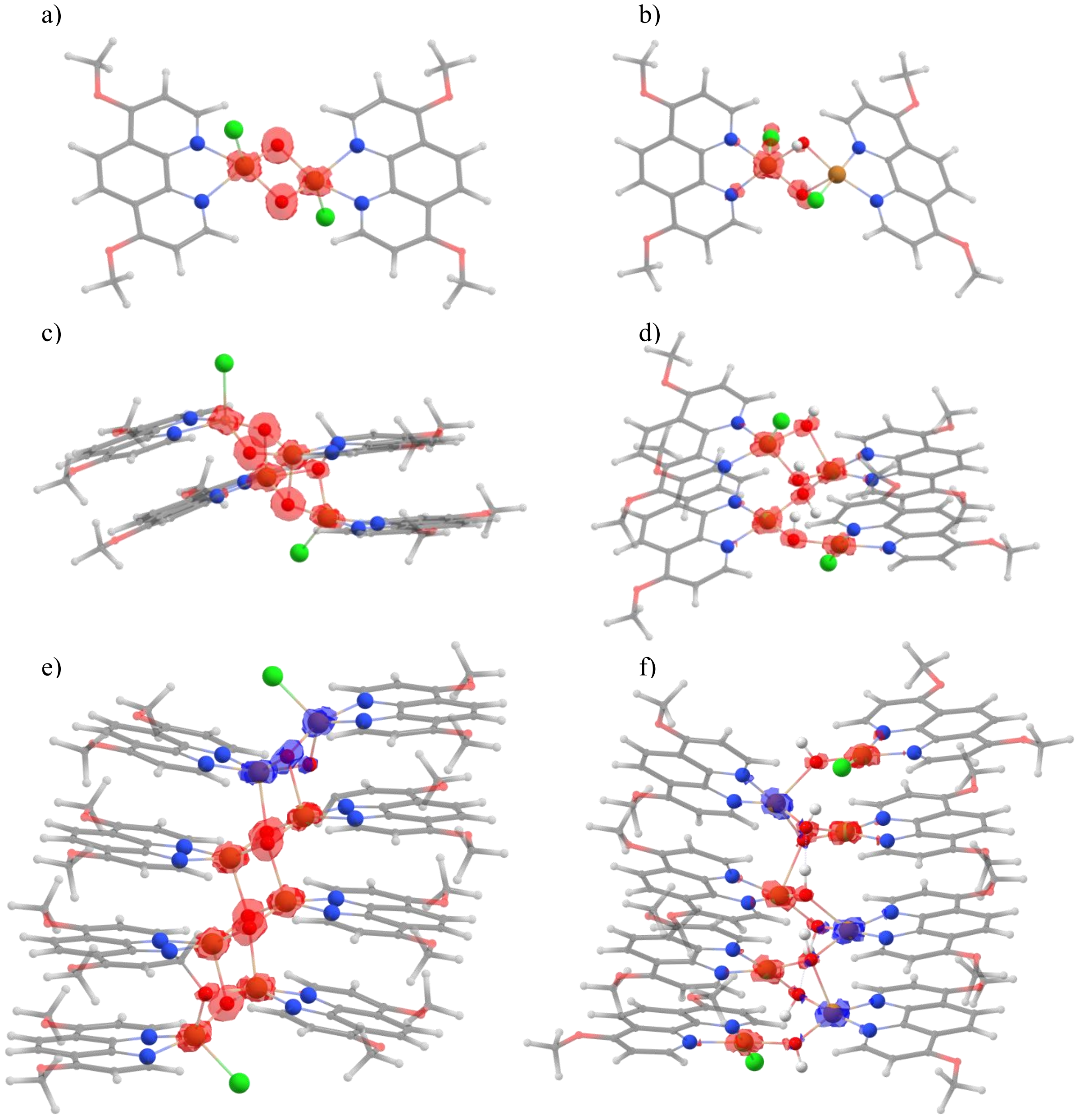

Figure S5. Spin density distributions of non-protonated (left) and protonated (right) form for dimer (a–b), tetramer (c–d), and octamer (e–f). Spin density isosurfaces are shown in red (α-spin) and blue (β-spin) with an isovalue of 0.027.

Table S1. Analysis of different catalyst models with their corresponding charges,[7] multiplicities (m),[8] and relative energies (ε). The most stable multiplicity is highlighted in bold numbers.

Dimer					
non-protonated			protonated		
charge	m	ε [kcal mol^{-1}]	charge	m	ε [kcal mol^{-1}]
-2	1	6.36	0	1	0.77
	**3**	**0.00**		**3**	**0.00**
Tetramer					
non-protonated			protonated		
Non-protonated	m	ε [kcal mol^{-1}]	Protonated	m	ε [kcal mol^{-1}]
charge -2	1	9.23	charge 2	1	1.3
	3	5.53		3	1.56
	**5**	**0.00**		**5**	**0.00**
Octamer (crystalline **Cat3** sample, cf. Figure S1)					
non-protonated			protonated		
Non-protonated	m	ε [kcal mol^{-1}]	Protonated	m	ε [kcal mol^{-1}]
charge -2	1	5.65	charge 6	1	4.01
	3	4.32		**3**	**0.00**
	**5**	**0.00**		5	1.61
	7	0.36		7	0.07
	9	0.83		9	1.50

[7] The formal charges of key atoms in the model systems were assigned as follows: Cu^{2+} (d^9, +2), O^{2-} (–2), and Cl^- (–1).

[8] Spin multiplicity was determined based on the number of unpaired electrons, following the standard formula $m = 2S + 1$, where S is the total spin quantum number, equal to half the number of unpaired electrons in the system. Since each Cu^{2+} center contains one unpaired electron, the maximum spin state corresponds to full ferromagnetic coupling, with all spins aligned. In this idealized limit, the expected m values are: 3 for the dimer (2 unpaired electrons, $S = 1$), 5 for the tetramer (4 unpaired electrons, $S = 2$), and 9 for the octamer (8 unpaired electrons, $S = 4$). However, such fully ferromagnetic coupling is rare in real systems, where exchange interactions between neighboring Cu^{2+} centers often lead to partial or complete antiferromagnetic alignment, effectively reducing the total spin of the system. Therefore, for each model, DFT calculations were performed across a range of reasonable multiplicities, from low-spin to high-spin configurations. The spin configuration with the lowest total energy was designated as the ground-state multiplicity.

Table S2. Computed average bond distances within the model dimer, tetramer and octamer of **Cat3**. Units: Å.

Structure		Cu-Cu	Cu-N	Cu-O	Cu-N/O
**dimer**	non-protonated	2.815	2.132	1.197	2.024
	protonated	2.956	1.987	2.025	2.006
**tetramer**	non-protonated	2.772	2.088	1.984	2.036
	protonated	3.075	2.038	2.149	2.093
**octamer**	non-protonated	2.772	2.073	1.994	2.033
	protonated	3.542	2.023	2.046	2.034

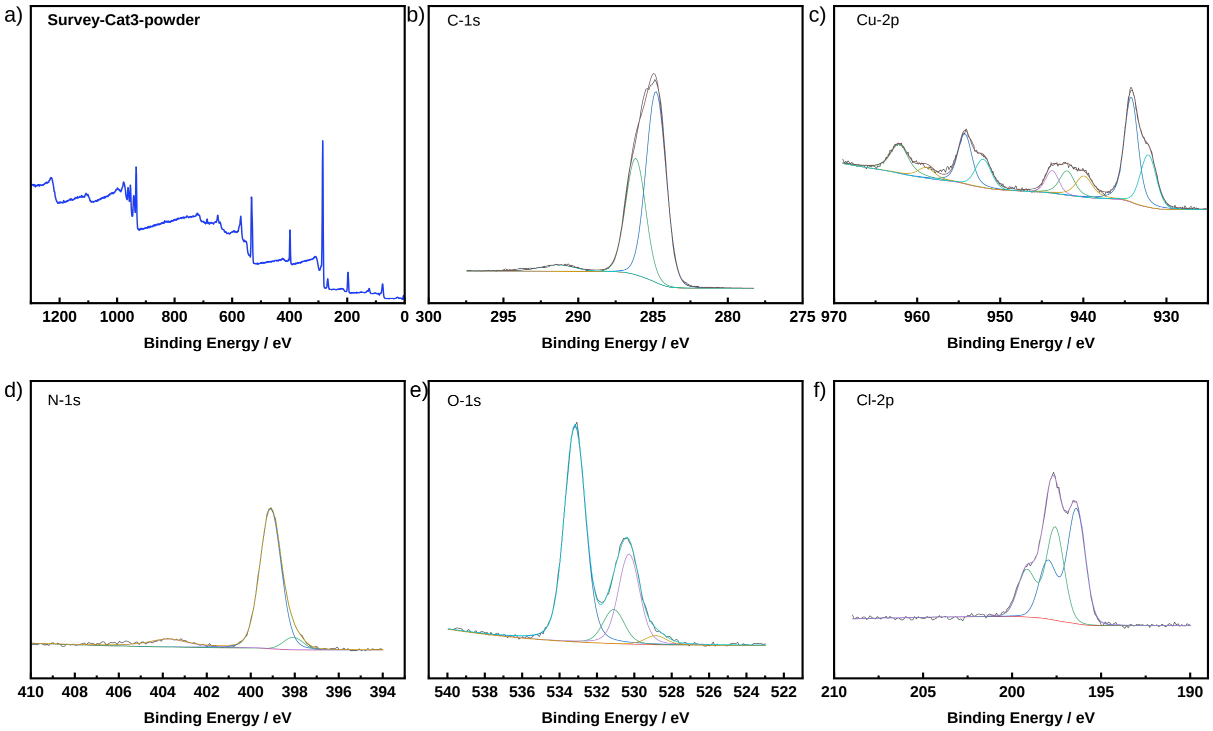

Figure S6. XPS scans of a) survey, b) the C 1s, c) Cu 2p, d) N 1s, e) O 1s and f) Cl 2p of as-prepared **Cat3** powder.

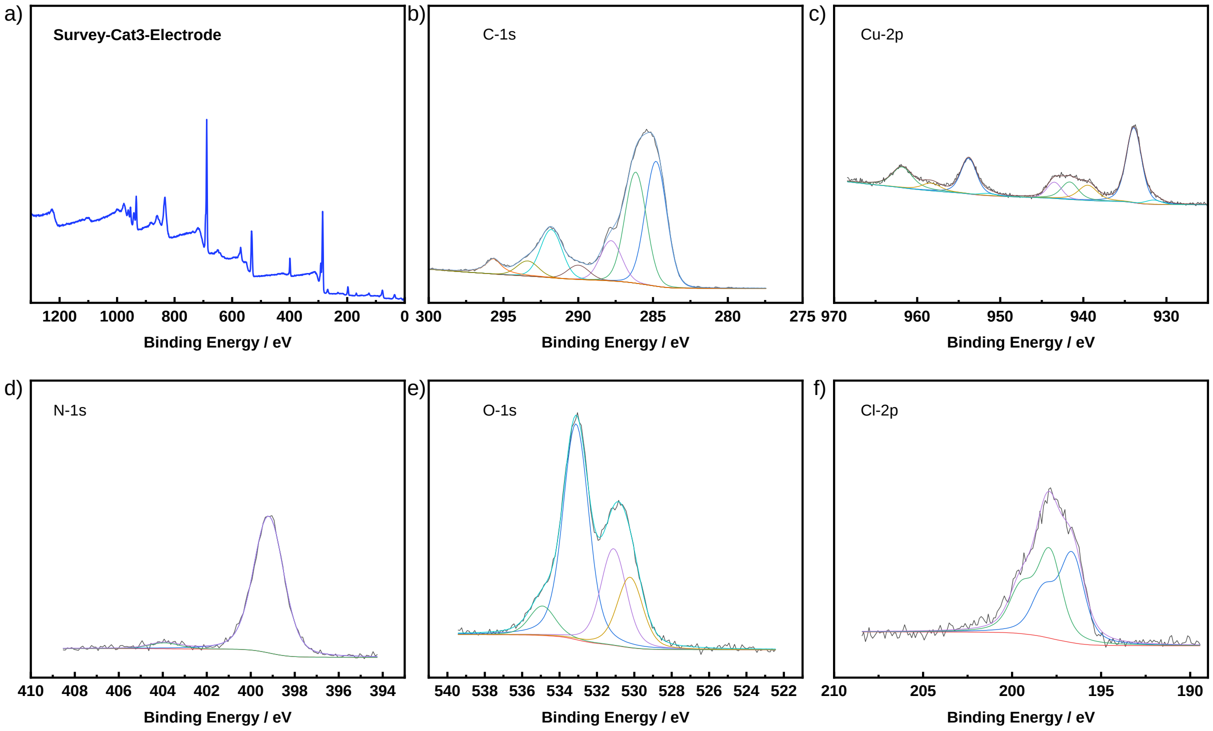

Figure S7. XPS scans of a) survey, b) the C 1s, c) Cu 2p, d) N 1s, e) O 1s and f) Cl 2p of a **Cat3**-modified carbon electrode. The intense F 1s peak in the survey spectra originated in the used Nafion-binder.

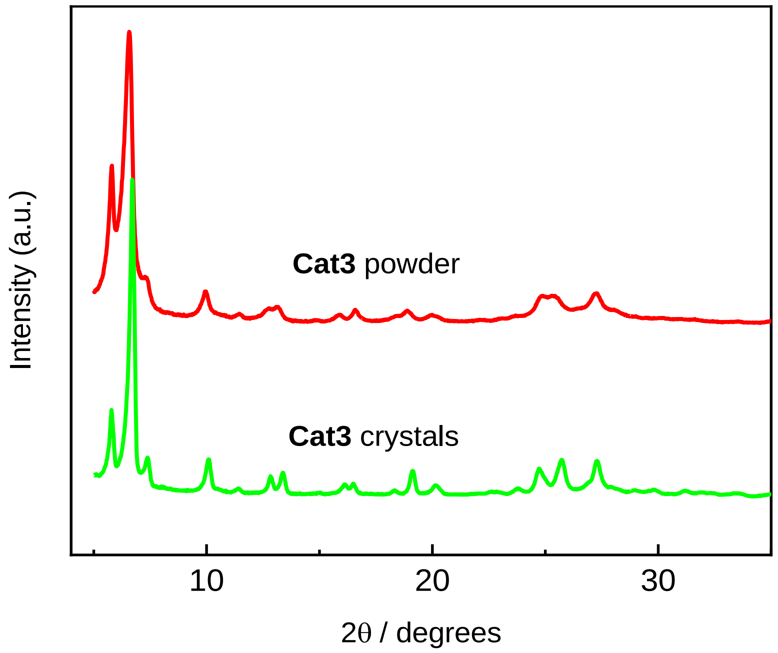

Figure S8. P-XRD patterns of as-prepared **Cat3** powder (red) and **Cat3** crystals (green) obtained from recrystallization using the autoclave procedure described in the Experimental Section.

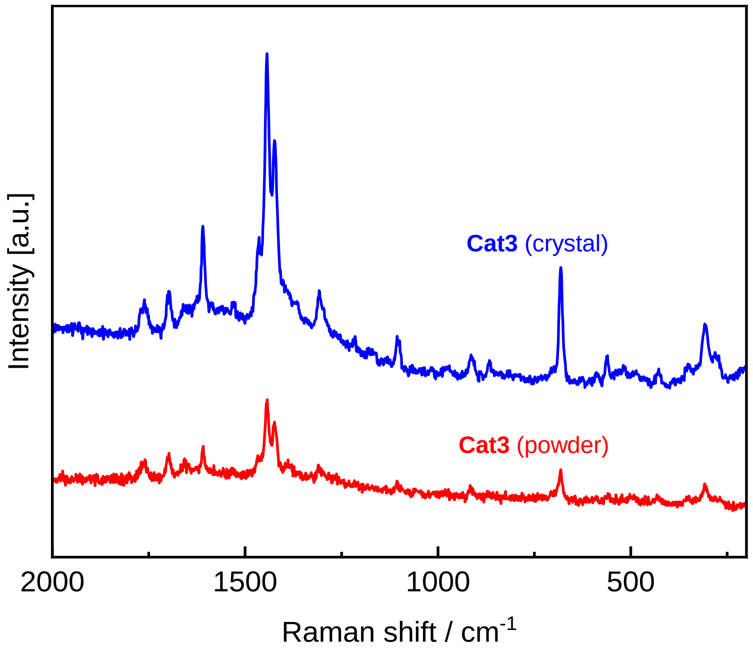

Figure S9. Comparison between the experimental Raman spectra (633 nm excitation) of **Cat3** powder and **Cat3** crystals.

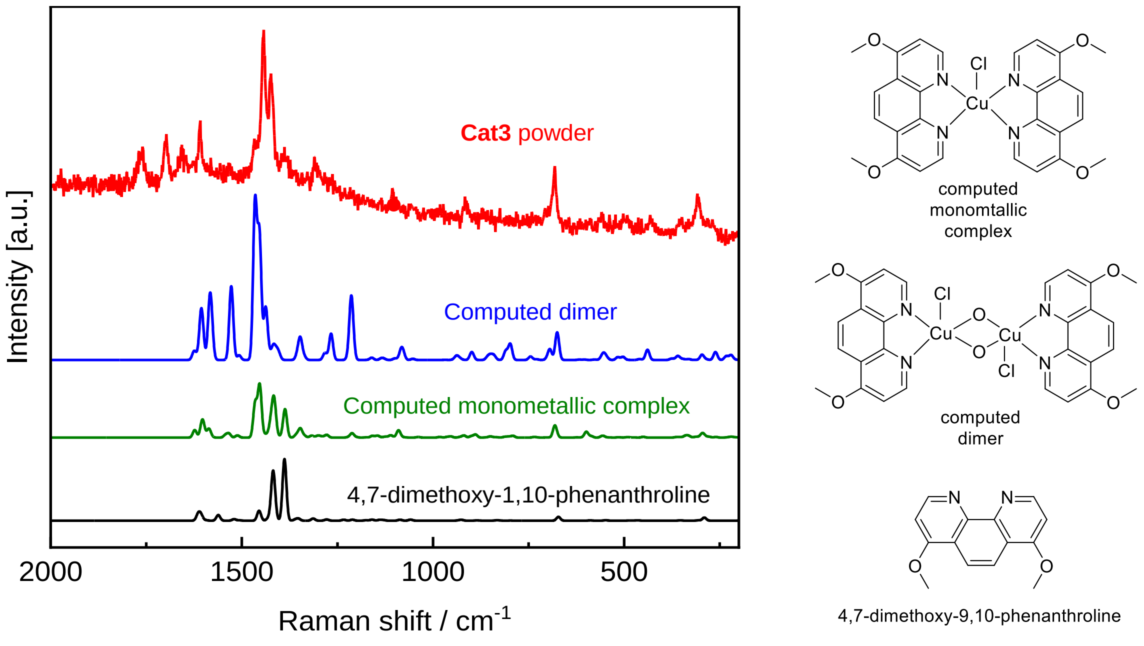

Figure S10. Comparison between the experimental Raman spectrum of **Cat3** powder (633 nm excitation) and computed spectra of a hypothetical dimer, a monometallic complex and 9,10-phenanthroline (with nitrogen atoms fixed during calculation). The Y-axis represents Raman activity (a.u.) for all simulated spectra; experimental data is plotted as relative intensity.

An enlarged representation of the Raman spectra for the pristine **Cat3** powder, along with four computationally modeled structures (**Cat3-tetramer**, **Cat3-dimer**, monomolecular complex, and 4,7-dimethoxy-1,10-phenanthroline), is shown in Figure S10. As structural distortion was observed during geometry optimization under vacuum conditions, four nitrogen atoms were fixed during Raman calculations of tetramer, dimer, and monomolecular complex to retain the planar configuration of the catalyst and to address the influence of geometry changes on the Raman spectra. Although this constraint provides a more realistic representation of the structure under experimental conditions, the calculated Raman spectra of different catalyst on partially fixed and fully relaxed geometries share the same spectral features. To facilitate the comparison, all spectra were normalized, and theoretical spectra were additionally scaled down by the factor of 0.9956 and vertically offset for clarity.[9]

The **Cat3** powder (experimental) spectrum shows strong bands centered around 429, 738, 1052, 1314, 1428, 1455 and 1609 cm^{-1}, which are attributed to metal-oxo stretching, ligand deformation, and skeletal vibrational modes of the binuclear framework. The **Cat3-dimer** model reproduces several of these features, showing prominent calculated Raman activities at 675, 694, 797, 810, 1078, 1214, 1266, 1346, 1438, 1454, 1465, 1528, 1582, and 1606 cm^{-1}, many of which overlap with key experimental peaks. In particular, the strong band at 1438 cm^{-1} (activity = 455 a.u.) and the peak at 1454 cm^{-1} (activity = 637 a.u.) closely match the major experimental bands at ~1429 and 1455 cm^{-1}, indicating that these vibrational modes are well preserved in the simplified dimer model and are best attributed to aromatic C–C and C–N stretching vibrations within the phenanthroline-type ligand framework. Their strong Raman intensities in the dimer suggest that metal-ligand electronic coupling within the dinuclear Cu core plays an important role in enhancing these ligand-centered vibrations. Consistent with this trend, the **Cat3-tetramer** model also exhibits strong Raman activity in the 1400–1600 cm^{-1} region, with prominent calculated peaks at 1436, 1454, 1465, 1528, 1582, and 1606 cm. In addition, bands at 738, 1076, and 1211 cm^{-1} also align with experimental observations, corresponding to skeletal ligand vibrations and metal–oxo stretching modes. The strong band at 1454 cm^{-1} (activity = 506 a.u.) and the peak at 1436 cm^{-1} (activity = 273 a.u.) are particularly noteworthy, supporting the vibrational enhancement from Cu–ligand interactions. These overlaps further validate the tetrameric structural motif and confirm that key Raman-active features are retained as nuclearity increases.

The monomolecular model shows only a limited number of weak vibrational features, consistent with the reduced complexity of its coordination environment. It exhibits minor activity near 1076, 1211, and 1436 cm^{-1}, but lacks any intense response around 1430-1460 cm^{-1}, underscoring the importance of the dinuclear Cu core in generating the dominant Raman signature of **Cat3**. The 4,7-dimethoxy-1,10-phenanthroline model, although lacking the metal core, still displays moderate Raman activities at 1388, 1415, 1419, 1454, 1561, and 1606 cm^{-1}, matching some experimental bands in position but generally lower in signal, consistent with the absence of Cu–oxo core contributions. The strongest mode at 1388 cm^{-1} (activity = 576 a.u.) is likely to correspond to an intraligand deformation mode within the phenanthroline ring.

Taken together, both the **Cat3-dimer** and **Cat3-tetramer** models capture key vibrational features observed in the experimental spectrum, particularly in the 1400-1600 cm^{-1} range, supporting the proposed structural motifs and validating the DFT-optimized geometries. The monomolecular complex model reinforces this conclusion by showing notably weaker and more sparse vibrational signals. In contrast, the 4,7-dimethoxy-1,10-phenanthroline model lacks the Cu core and excibits less intense but still observable modes at 1388, 1415, and 1454 cm^{-1}, primarily arising from intra-ligand skeletal deformations. A comparison of the most intense calculated modes and their tentative assignments is provided in a separate excel file.

[9] M. K Kesharwani, B. Brauer, J. M. Martin, *J. Phys. Chem. A* **2015**, *119*, 1701-1714.

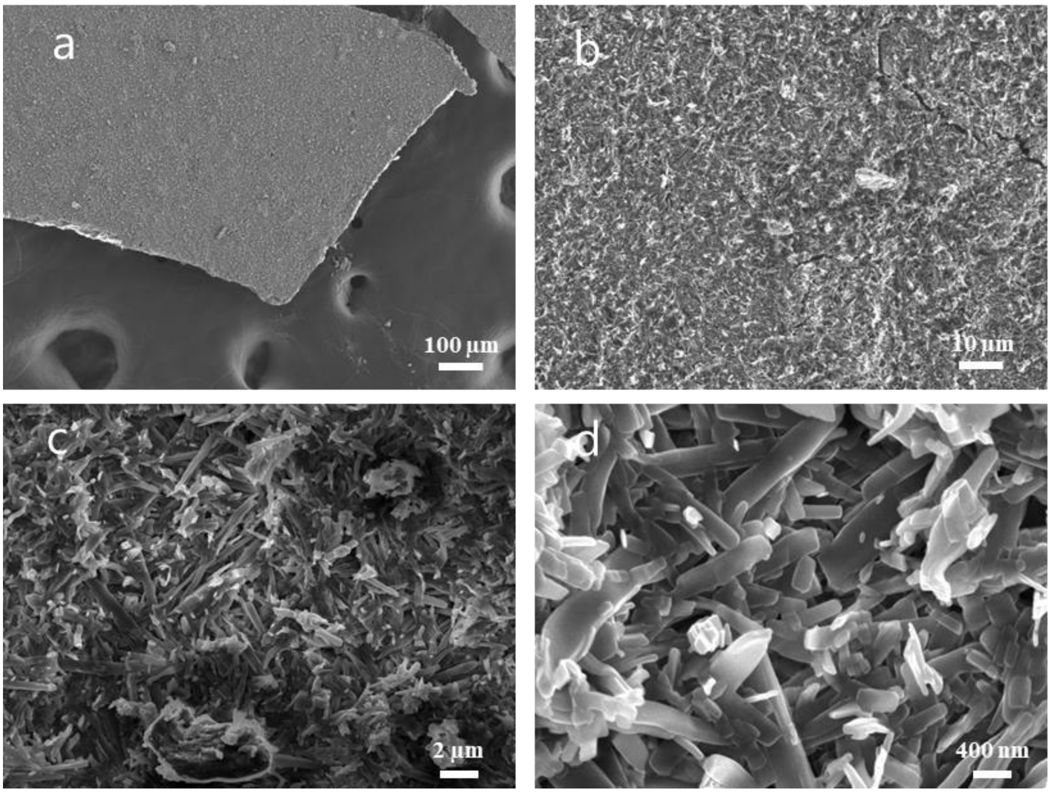

Figure S11. a-d) SEM images of **Cat3** powder (for details of sample preparation, see the Experimental Section).

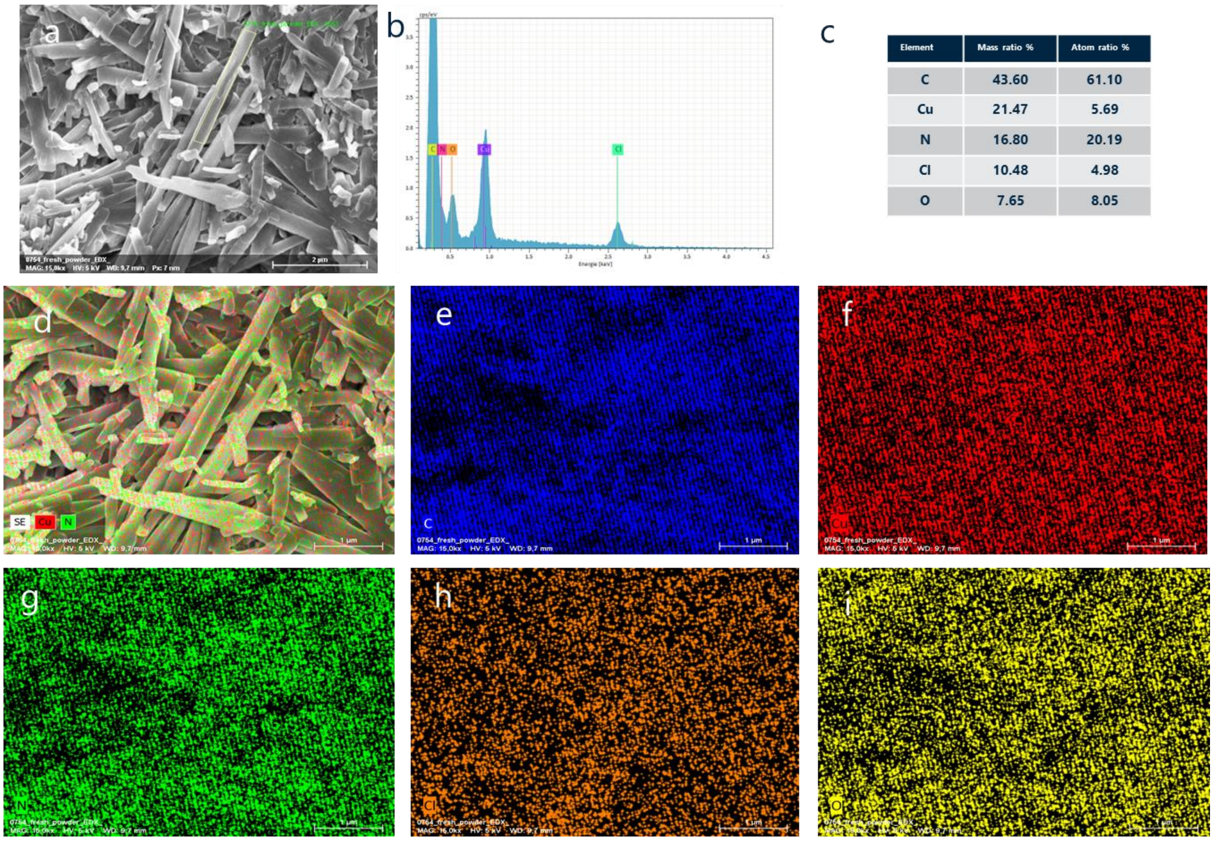

Element	Mass ratio %	Atom ratio %
C	43.60	61.10
Cu	21.47	5.69
N	16.80	20.19
Cl	10.48	4.98
O	7.65	8.05

Figure S12. a) SEM image of **Cat3** powder. b,c) Results of elemental analysis carried out in the domain indicated in a). d-i) EDX mapping for the relevant elements.

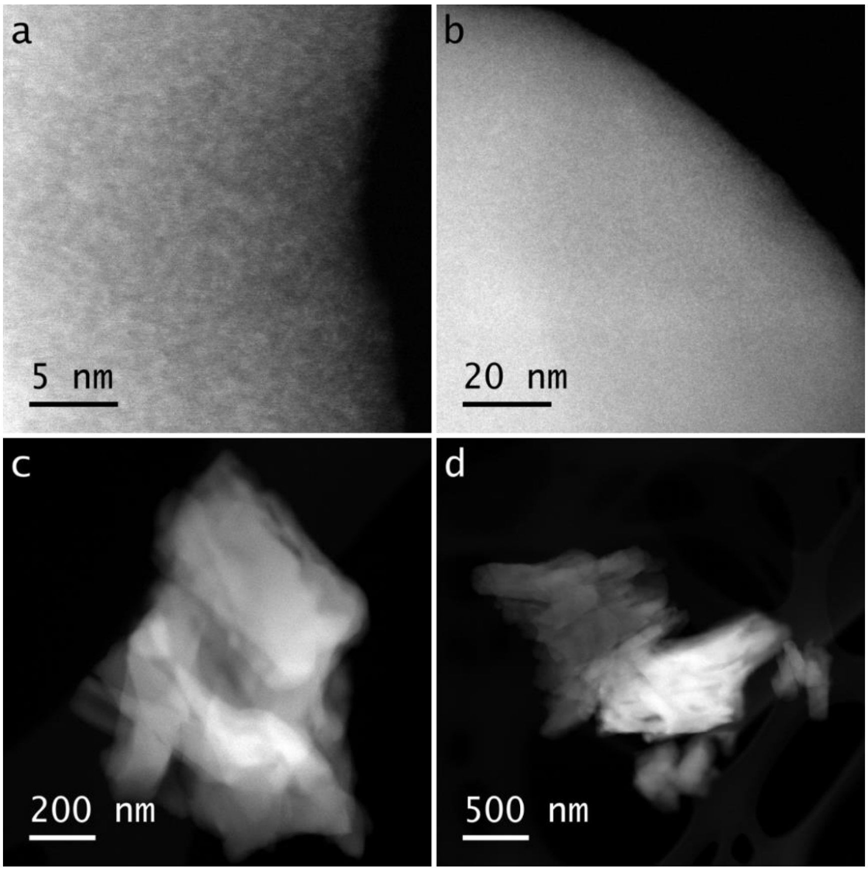

Figure S13. Selected HAADF-STEM images of **Cat3**. The catalyst powder was deposited in dry form on a Ni grid.

3. Behavior of Cat3 in liquid CO_2-saturated electrolyte

3.1. Electrochemical studies in H-cells

Table S3. Initial ligand screening using commercially available phen derivatives.

Ligand	Electrolyte	Potential (V vs. RHE)	Current density (mA cm^{-2})	FE of H_2 (%)	FE of C_2H_4 (%)
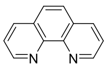	0.1 M $KHCO_3$	-1.2	2.86	6.07	35.62
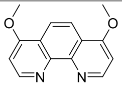	0.1 M $KHCO_3$	-1.2	2.84	9.71	21.50
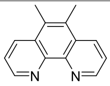	0.1 M $KHCO_3$	-1.2	0.50	21.46	13.59
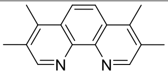	0.1 M $KHCO_3$	-1.2	0.45	7.83	14.74

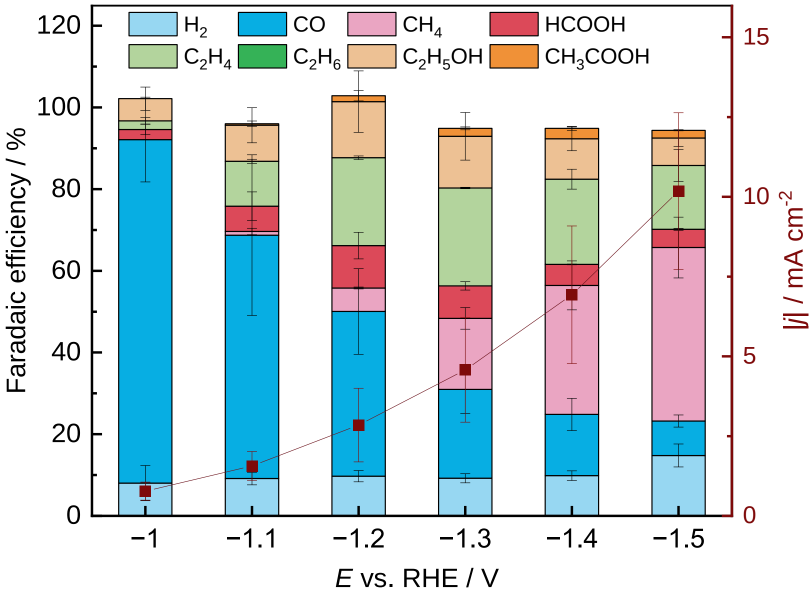

Figure S14. Product distributions and average current densities for electrochemical reduction of CO_2 in aqueous 0.1 M $KHCO_3$ solution using a **Cat3**-modified carbon paper electrode at different potentials (H-type cell).

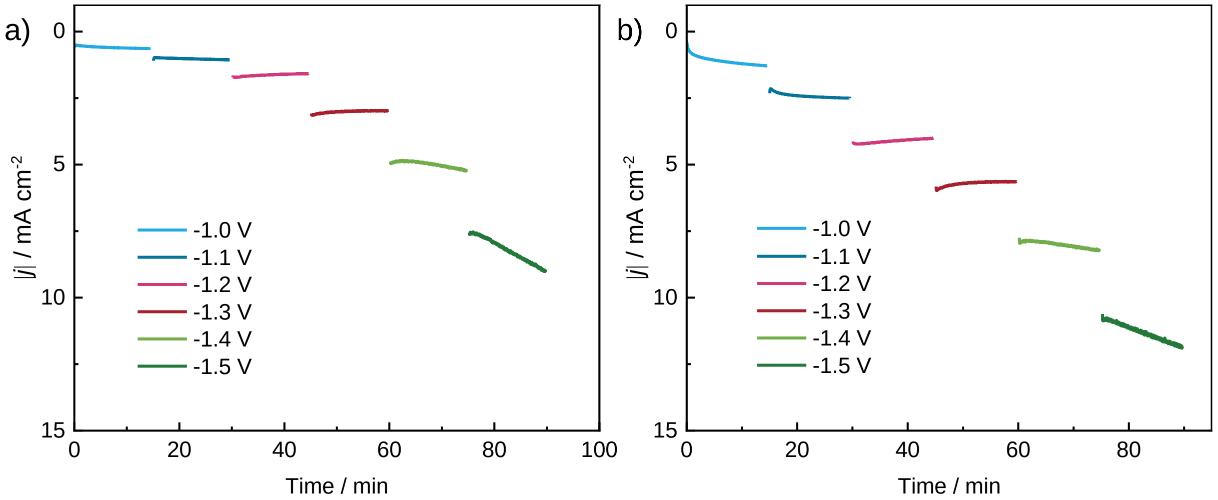

Figure S15. Current density-time profiles for electrochemical reduction of CO_2 in aqueous a) 0.1 M $KHCO_3$ and b) 0.1 M $CsHCO_3$ solution, both obtained using a **Cat3**-modified carbon paper electrode at different potentials (H-type cell).

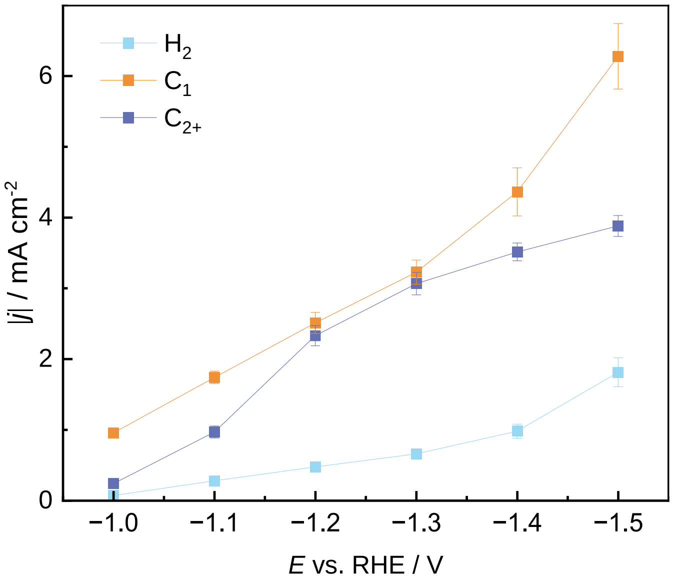

Figure S16. Partial current densities for H_2, C_1 and C_{2+} products for electrochemical reduction of CO_2 in aqueous 0.1 M $CsHCO_3$ solution using a **Cat3**-modified carbon paper electrode at different potentials (H-type cell).

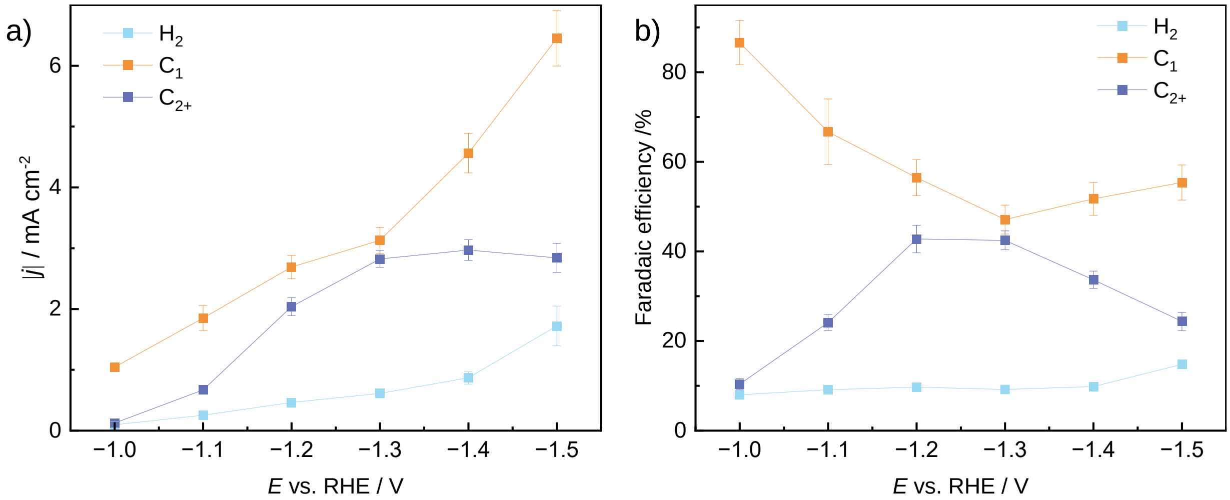

Figure S17. Electrochemical reduction of CO_2 in aqueous 0.1 M $KHCO_3$ solution using a **Cat3**-modified carbon paper electrode at different potentials (H-type cell). a) Partial current densities for H_2, C_1 and C_{2+} products. b) Comparison between the *FE*s of C_1 and C_{2+} products as well as H_2.

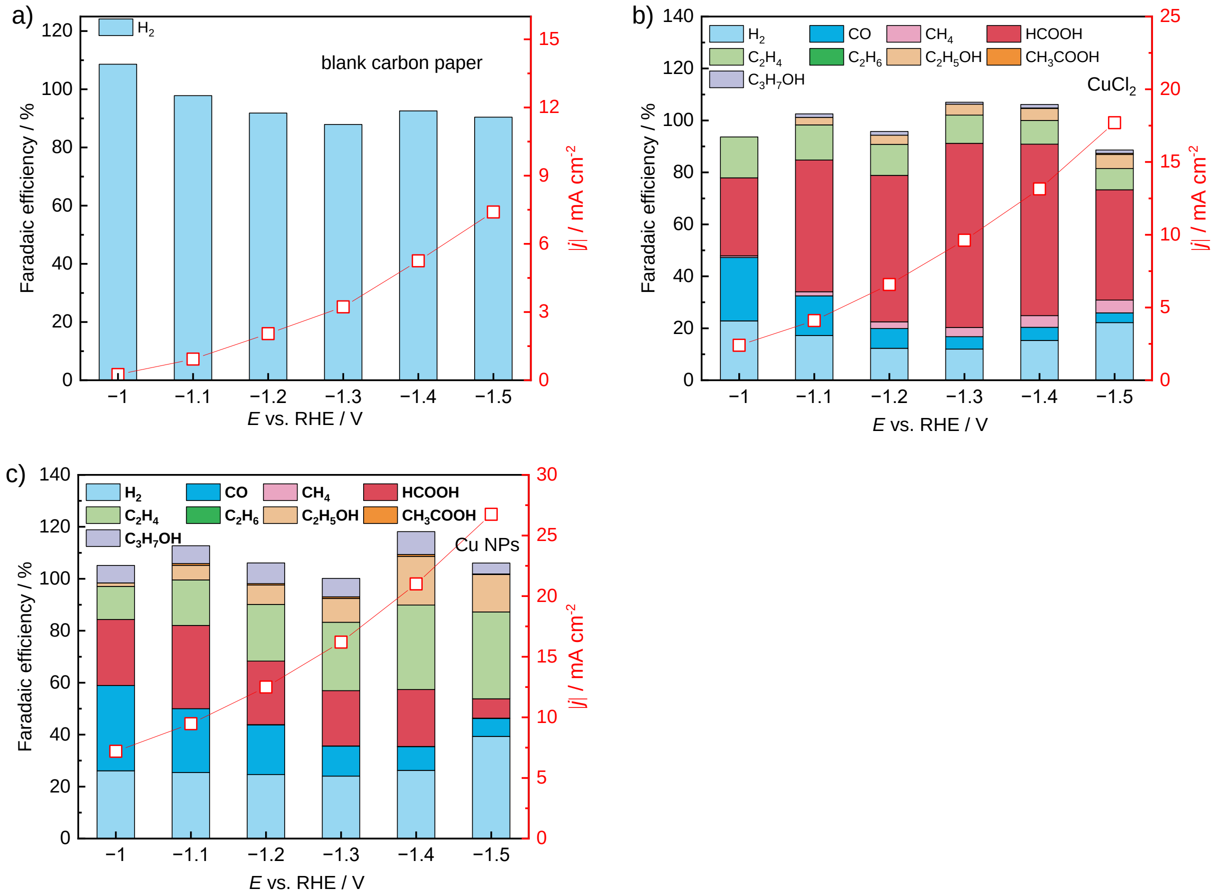

Figure S18. Control experiments (H-cell) using a) bare carbon paper, b) $CuCl_2$-modified carbon paper, and c) carbon paper modified with commercial Cu nanoparticles (25 nm) as working electrodes. $CuCl_2$ and Cu nanoparticles were drop-casted onto carbon paper following the same protocol described for preparation of **Cat3**-modified electrodes (0.5 mg cm^{-2}, identical with the mass loading of **Cat3**). Measurements were performed in an H-cell with 0.1 M $CsHCO_3$ as the electrolyte.

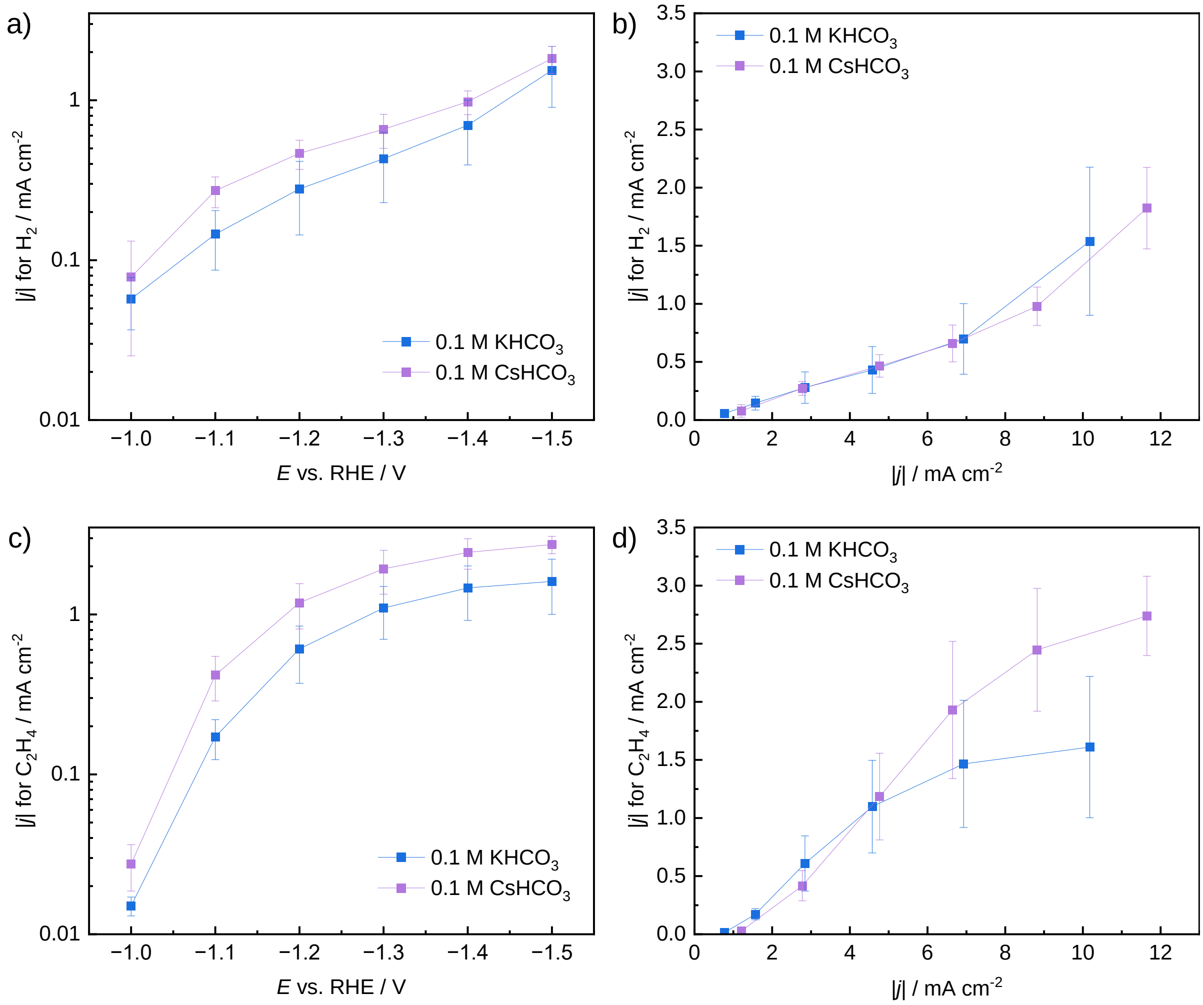

Figure S19. Comparison between results obtained from electrolysis in 0.1 M $CsHCO_3$ and 0.1 M $KHCO_3$ using a **Cat3**-modified electrode (H-type cell). Absolute values of partial current densities for a,b) H_2 and c,d) C_2H_4.

In previous studies on cation effects on eCO_2RR, Cs^+ has been highlighted as a more effective promoter for eCO_2RR compared to K^+ across various catalyst systems. The benign effect of Cs^+ on the catalytic rate has been ascribed to

(1) its weaker hydration shell compared to smaller cations, which supports maintenance of a relatively low interfacial pH and thereby increases local CO_2 concentration;[10]
(2) the enhancing effect of the smaller hydrated ion radius on the surface charge density and the associated electric field (improving surface-charge dependent reaction energetics);[11] and
(3) the stabilization of key intermediates for eCO_2RR.[12]

These effects can individually influence reaction kinetics or act synergistically within the overall electrocatalytic reaction network.

To understand the benign effect of Cs^+ cations for eCO_2RR reactivity on our **Cat3** (see Figure 2b vs. Figures S13, S14, and S17), electrochemical impedance spectroscopy (EIS) was conducted using the H-cell configuration (identical to the H-cell performance screening) across a frequency range from 10^5 Hz to 1 Hz at –1.2 V vs. RHE. This potential enables significant formation of C_2 products, while minimizing strong gas bubble generation that would occur at higher potentials/current densities.

In the Nyquist plots (Figure S20a), the first intersection with the real part (x-axis) is, in principle, attributed to the Ohmic resistance between the working and reference electrode, while the second intersection is tentatively assigned to polarization resistance, primarily governed by reaction kinetics. Overall, while both $CsHCO_3$ and $KHCO_3$ electrolytes display comparable Ohmic resistance, the Cs^+ tends to lower the polarization resistance, suggesting enhanced reaction kinetics. However, the Nyquist plot alone provides is insufficient to resolve the overlapping contributions of distinct charge, ion, and mass transfer processes at the electrolyte–catalyst interface. In an attempt to further disentangle these convoluted interfacial dynamics, we performed the Distribution of Relaxation Times (DRT) analysis of the EIS, following earlier studies.[13] This approach, in theory, enables the deconvolution of individual transport and kinetic contributions based on their characteristic relaxation frequencies.

In our approach, DRT analysis was performed using the RelaxIS Web-Tool,[14] with a regularization parameter of 10^{-3} as standard setting. The extracted DRT profiles are shown in Figure S20b. At –1.2 V, a sharp and prominent peak appears near 10^2 Hz and 10^3 Hz, which was previously assigned to eCO_2RR-related electron transfer (P2) and the coupled chemical bicarbonate/CO_2 equilibrium (P3).[13a] Interestingly, in 0.1 M $CsHCO_3$ electrolyte, both peaks render a smaller contribution than in 0.1 M $KHCO_3$, which may suggest that the enhanced eCO_2RR reactivity is attributable to improved electron transfer kinetics and a more favorable bicarbonate/CO_2 equilibrium (resulting in better local CO_2 availability).

---

[10] M. R. Singh, Y. Kwon, Y. Lum, J. W. Ager, A. T. Bell, *J. Am. Chem. Soc.* **2016**, *138*, 13006–13012.

[11] a) J. Resasco, L. D. Chen, E. Clark, C. Tsai, C. Hahn, T. F. Jaramillo, K. Chan, A. T. Bell, *J. Am. Chem. Soc.* **2017**, *139*, 11277–11287. b) S. Ringe, E. L. Clark, J. Resasco, A. Walton, B. Seger, A. T. Bell, K. Chan, *Energy Environ. Sci.*, **2019**, *12*, 3001-3014.

[12] a) L. D. Chen, M. Urushihara, K. Chan, J. K. Nørskov, *ACS Catal.* **2016**, *6*, 7133–7139. b) M.C.O. Monteiro, F. Dattila, B. Hagedoorn, R. García-Muelas, N. López, M.T.M. Koper, *Nature Catal.* **2021**, *4*, 654–662.

[13] a) Q. Chen, A. Kube, D Kopljar, K. A. Friedrich, *ACS Energy Lett.* **2024**, *9*, 6096-6103. b) S. Dierickx, A. Weber, E. Ivers-Tiffée, *Electrochim. Acta* **2020**, *355*, 136764. c) S. W. Lee, T. H. Nam, S. H. Lee, T. Ishihara, J. T. S. Irvine, T. H. Shin, *Energy Environ. Sci.* **2025**, 18, 1205.

[14] For further information, see: https://relaxis-drt.rhd-instruments.de/.

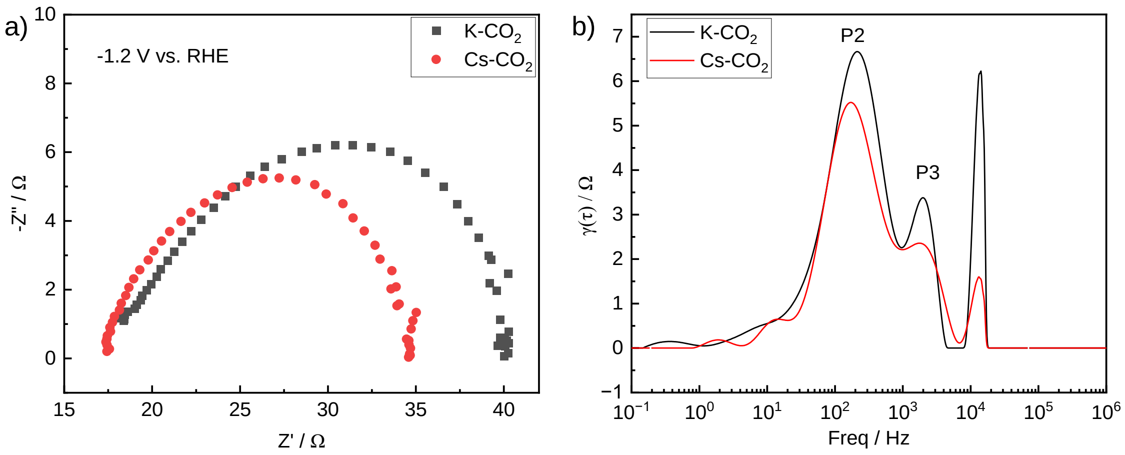

Figure S20. Results of EIS analysis of a **Cat3**-modified electrode in the H-type cell at -1.2 V vs. RHE. a) Nyquist plot. b) Distribution of relaxation time (DRT).

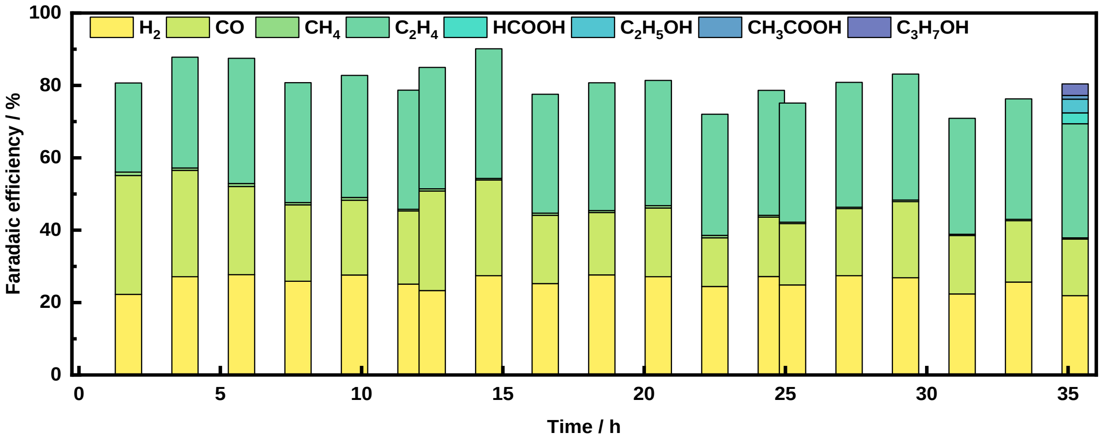

Figure S21. Long-term electrolysis under CO_2 in 0.1 M $CsHCO_3$ using a **Cat3**-modified carbon paper electrode (compare Figure 2e). Progression of the *FE*s for individual gas phase products (liquid phase analysis was carried out by ^{1}H NMR spectroscopy after completed electrolysis).

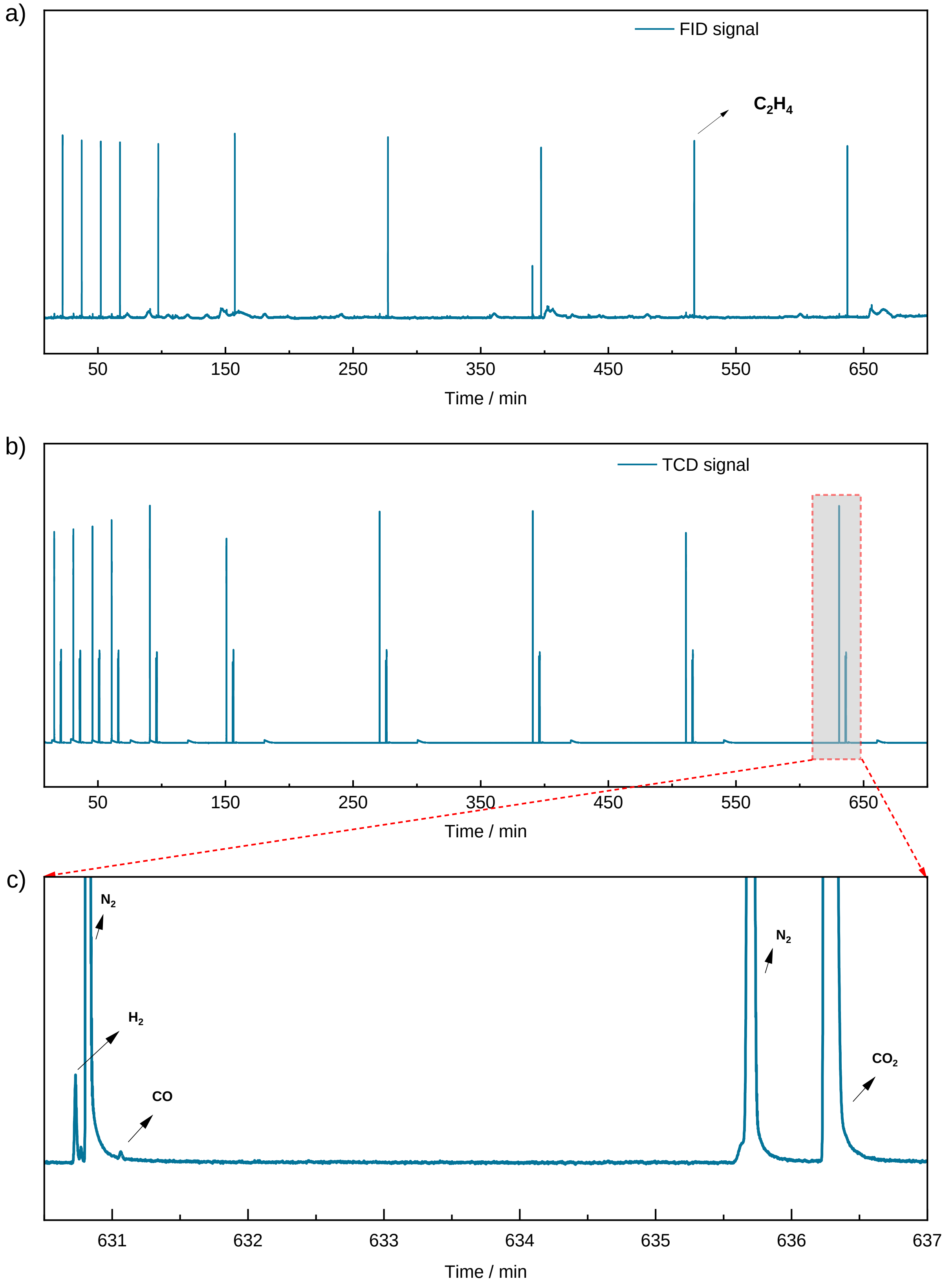

Figure S22. Example headspace a) GC-FID and b,c) GC-TCD traces during long-term electrolysis under CO_2 in 0.1 M $CsHCO_3$.

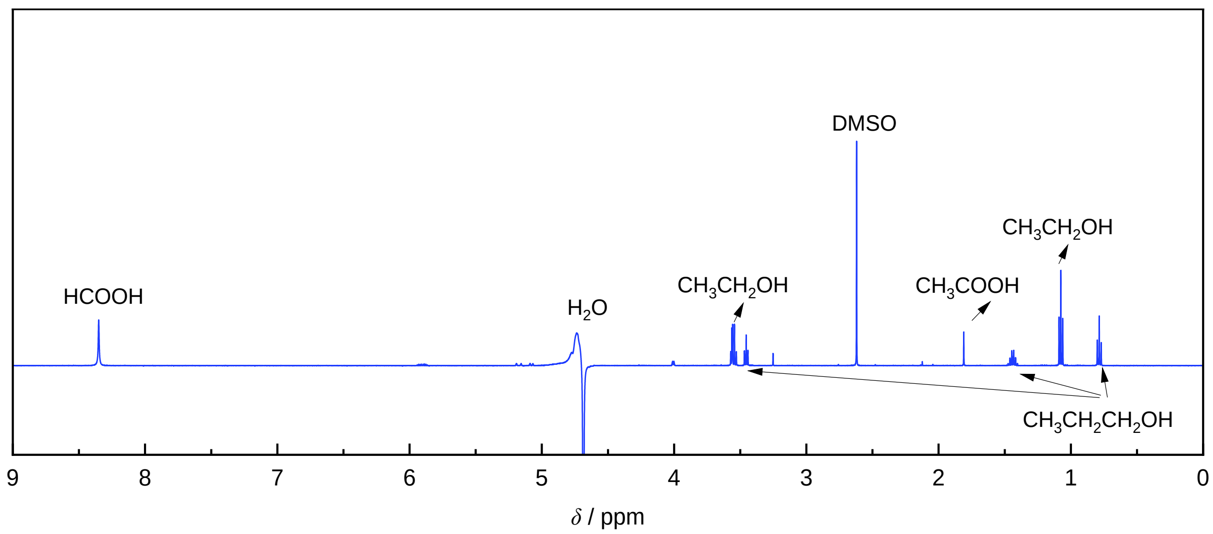

Figure S23. ^{1}H NMR spectrum of an electrolyte aliquot taken after long-term electrolysis under CO_2 in 0.1 M $CsHCO_3$ using a **Cat3**-modified carbon paper electrode (H-cell).

3.2. Post-electrolysis characterization of Cat3-modified carbon electrodes

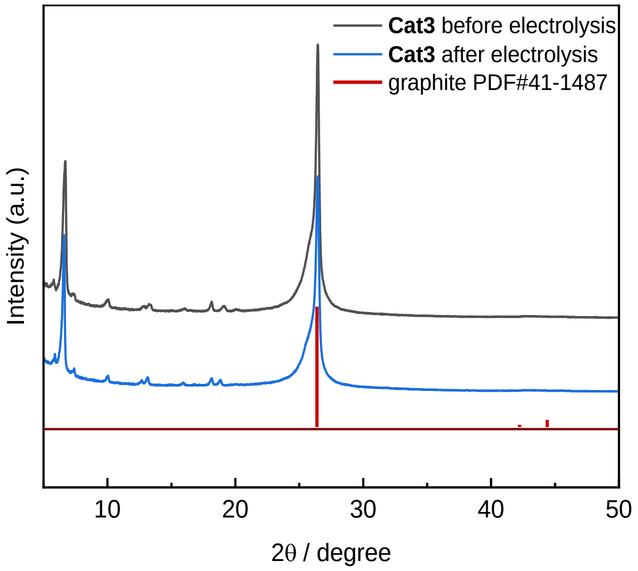

Figure S24. P-XRD patterns recorded using a **Cat3**-modified carbon paper electrode before and after electrolysis (reference data for graphite taken from PDF Database, International Center for Diffraction Data).

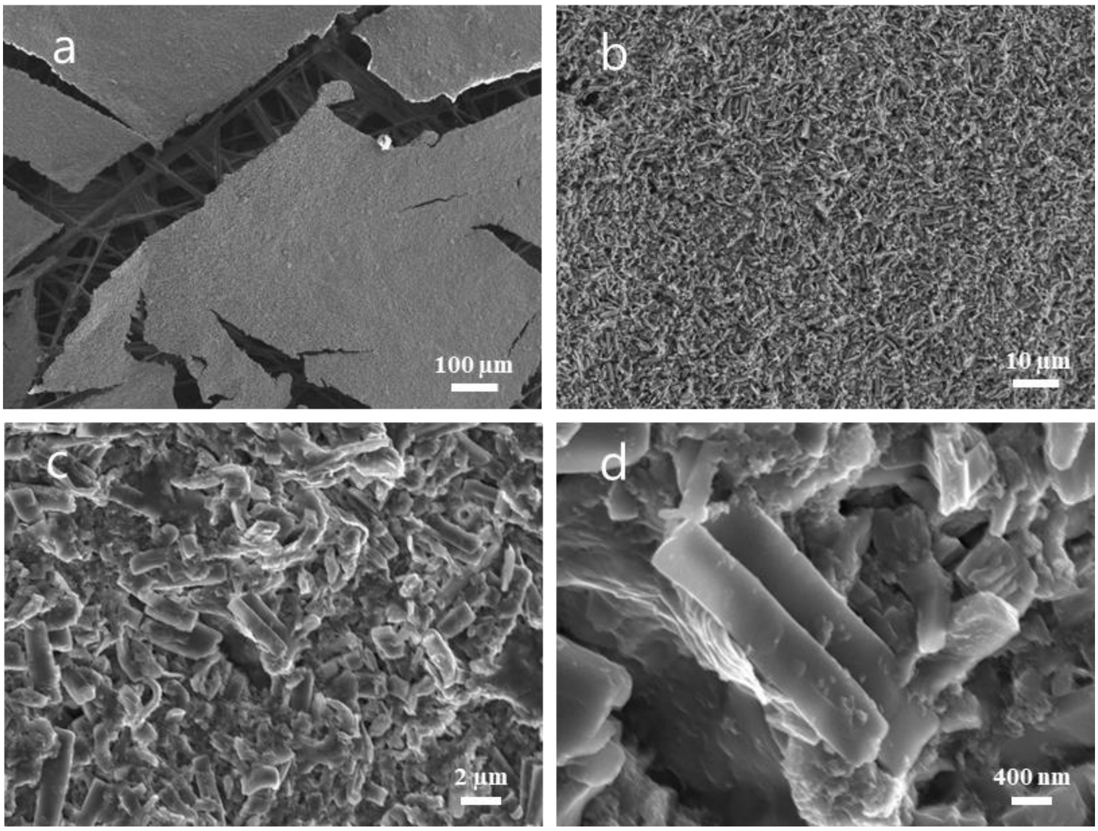

Figure S25. a-d) SEM images of a **Cat3**-modified carbon paper electrode before electrolysis (for details of electrode preparation, see the Experimental Section).

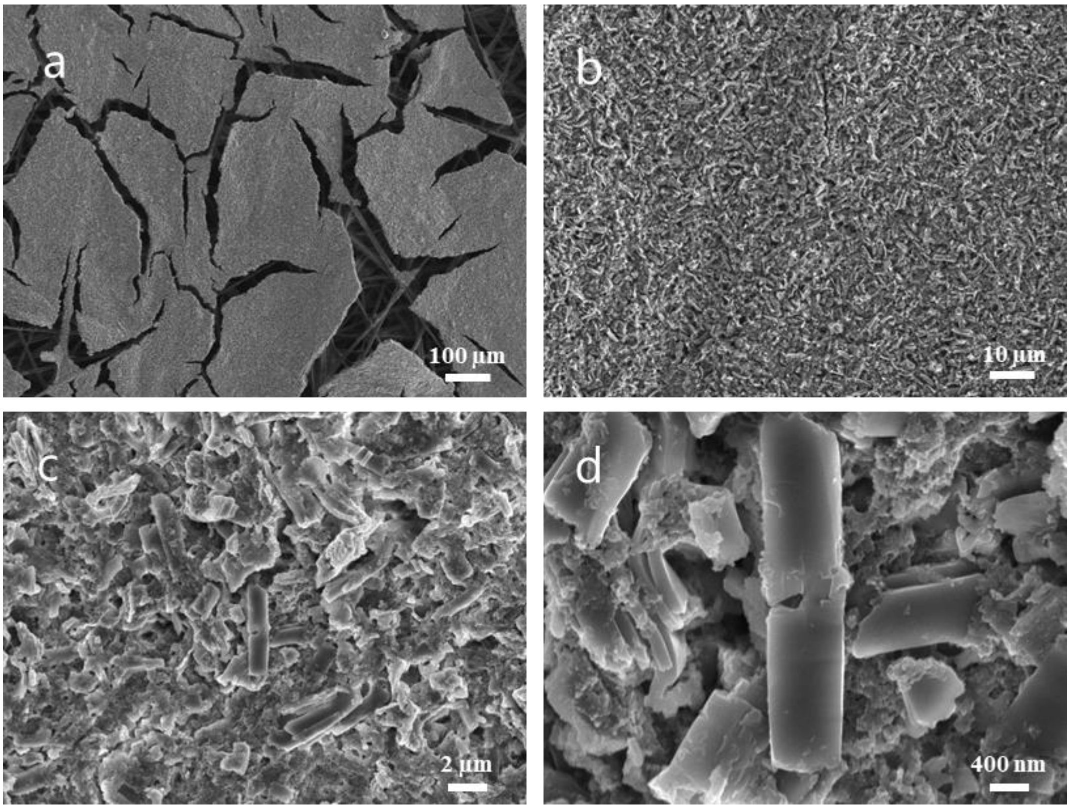

Figure S26. Post-electrolysis (H-cell, CPE at –1.2 V vs. RHE, 0.1 M $KHCO_3$) characterization of a **Cat3**-modified electrode with SEM (for details of electrode preparation and electrolysis, see the Experimental Section).

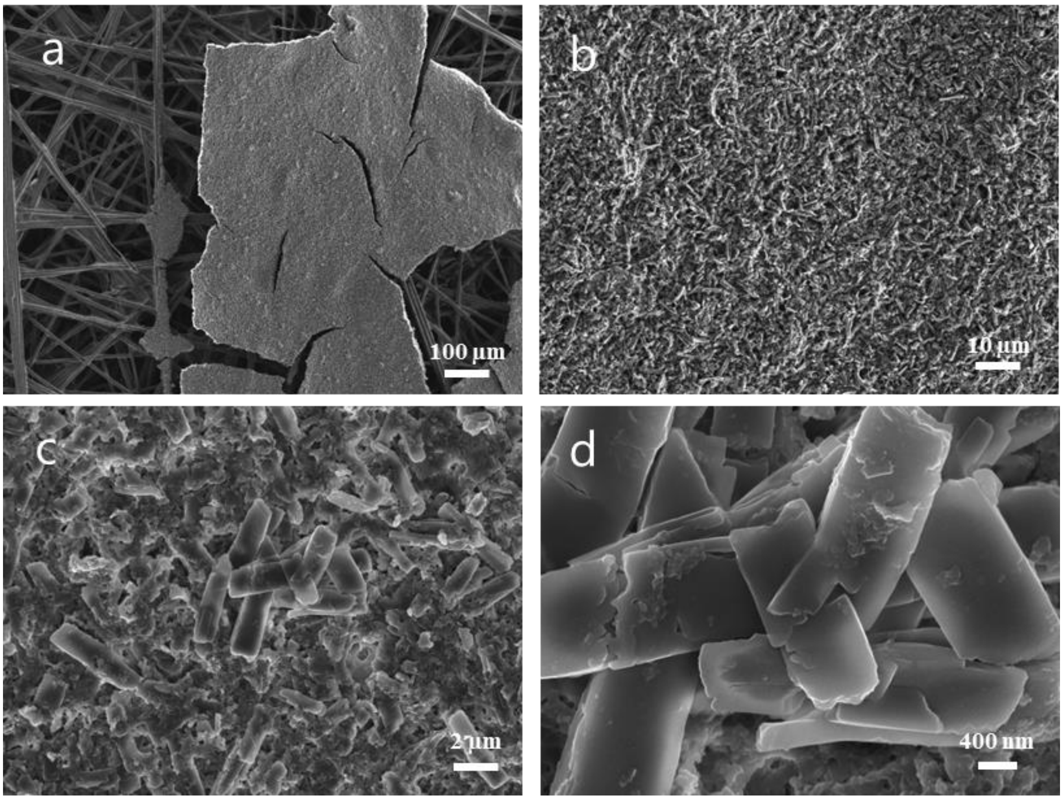

Figure S27. Post-electrolysis (H-cell, CPE at –1.2 V vs. RHE, 0.1 M $CsHCO_3$) characterization of a **Cat3**-modified electrode with SEM (for details of electrode preparation and electrolysis, see the Experimental Section).

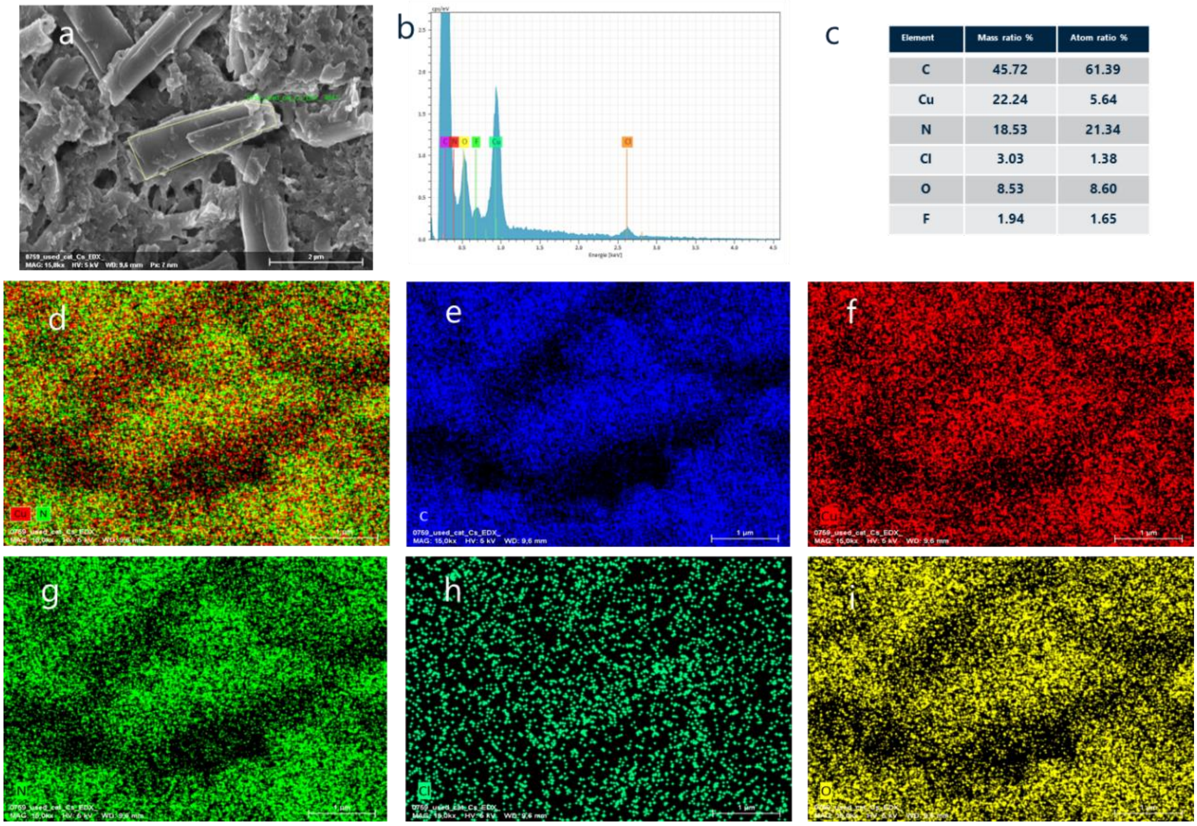

Figure S28. a) SEM image of a **Cat3**-modified carbon paper electrode after CPE at –1.2 V vs. RHE in 0.1 M $CsHCO_3$ (for details of electrode preparation and electrolysis, see the Experimental Section). b, c) Results of elemental analysis carried out in the domain indicated in a). d-i) EDX mapping for the relevant elements.

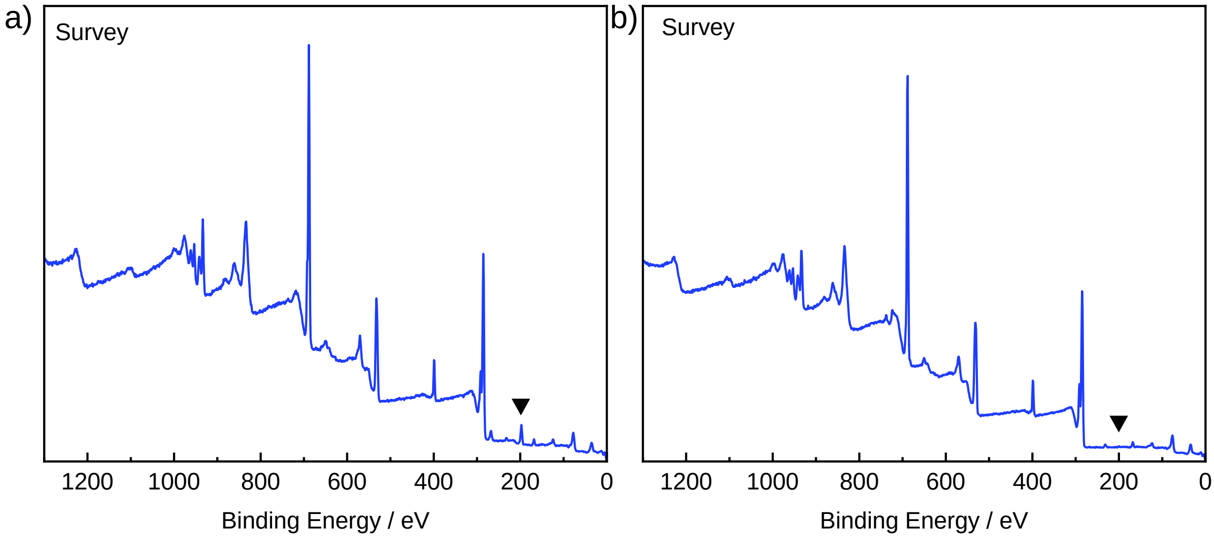

Figure S29. XPS survey spectra of a **Cat3**-modified electrode before (a) and after (b) CPE at –1.2 V (aqueous CO_2-saturated 0.1 M $CsHCO_3$ electrolyte). A clear Cl 2p peak around 200 eV is only present in the spectrum of the fresh catalyst. The intense F 1s peak in the survey spectra originated in the used Nafion-binder. For details of electrode preparation and electrolysis, see the Experimental Section).

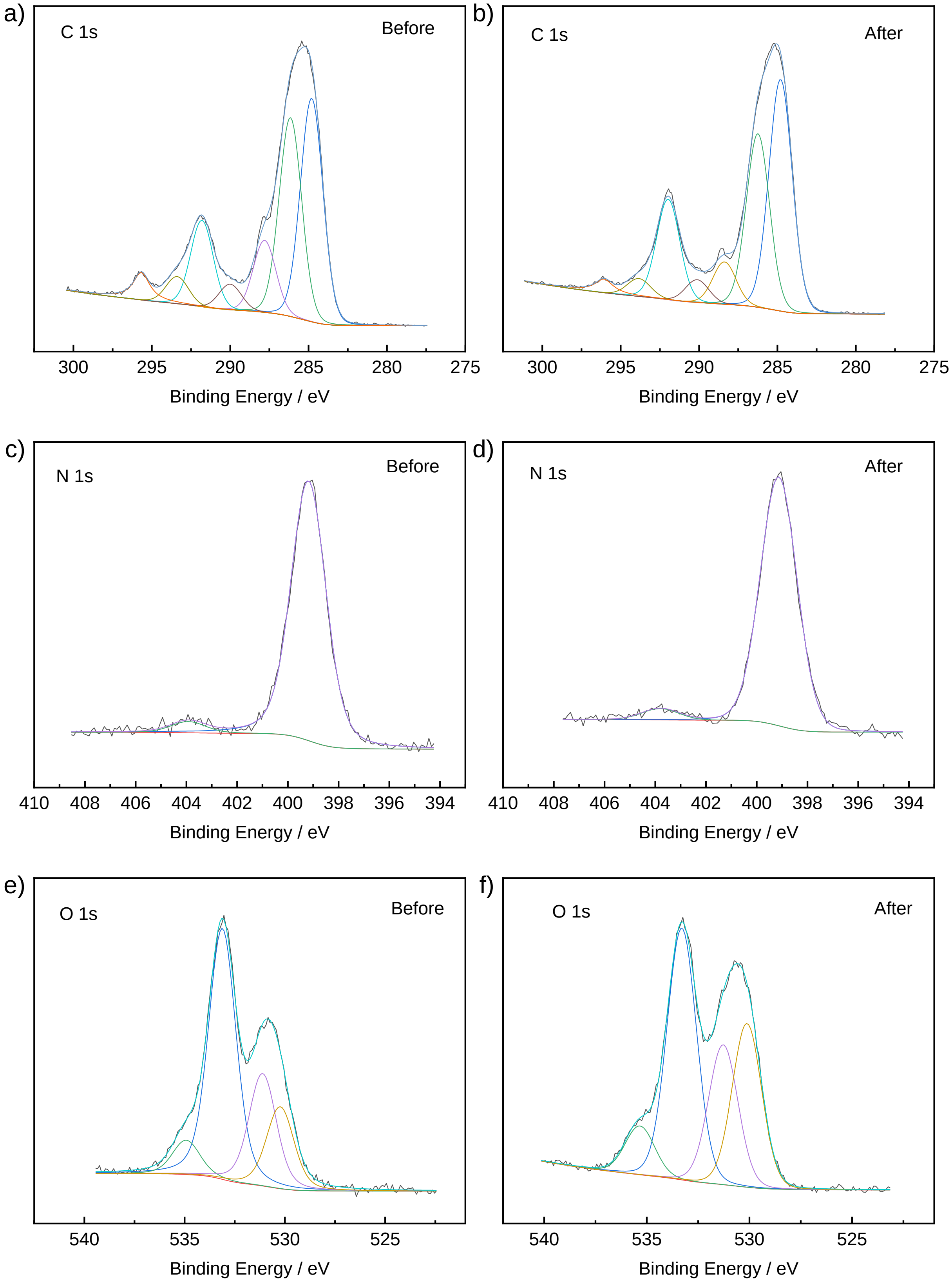

Figure S30. XPS analysis of a **Cat3** modified electrode before (left) and after electrolysis (right). Electrolysis conditions: **Cat3** modified electrode was measure in H-cell under -1.2 V vs. RHE using 0.1 M $CsHCO_3$ as electrolyte. (for details of electrode preparation and electrolysis, see the Experimental Section).

Table S4. XPS-based elemental analysis of **Cat3** (as-prepared **Cat3**-powder, **Cat3**-modified electrode before and after electrolysis at -1.2V vs. RHE in an H-cell using aq. 0.1 M $KHCO_3$ and $CsHCO_3$ electrolytes, respectively).

**Quant. / at.%**	**C**	**Cu**	**N**	**O**	**Cl**	**F**	**S**	**N/Cu**
As-prepared **Cat3** powder	67.3	4.3	9.0	15.5	3.9	/	/	2.09
Fresh electrode	58.3	2.8	5.1	11.4	1.9	20.5	/	1.82
Used electrode (0.1 M $KHCO_3$)	56.5	2.2	4.0	11.5	0.1	25.7	/	1.82
Used electrode (0.1 M $CsHCO_3$)	58.3	2.7	4.9	13.2	/	20.3	0.7	1.81

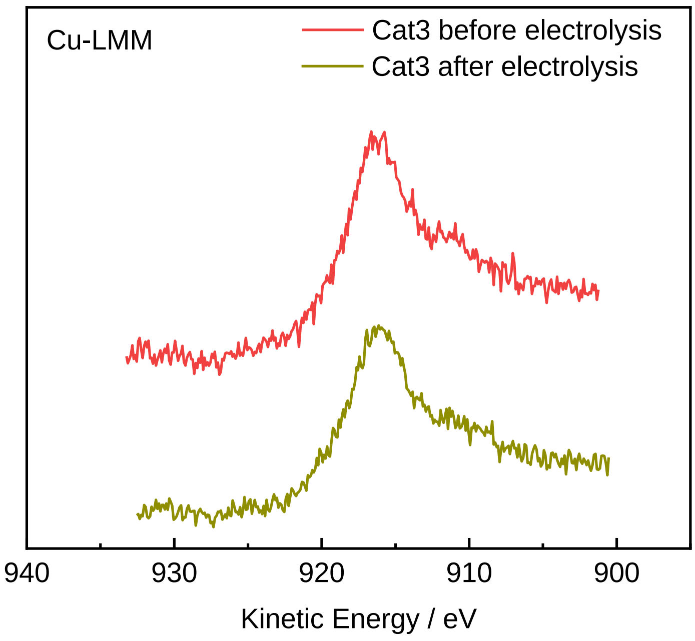

Figure S31. Cu Auger LMM spectra of a **Cat3**-modified carbon paper electrode before and after electrolysis.

3.3. *In situ* XAS analysis under potential control using a divided flow cell

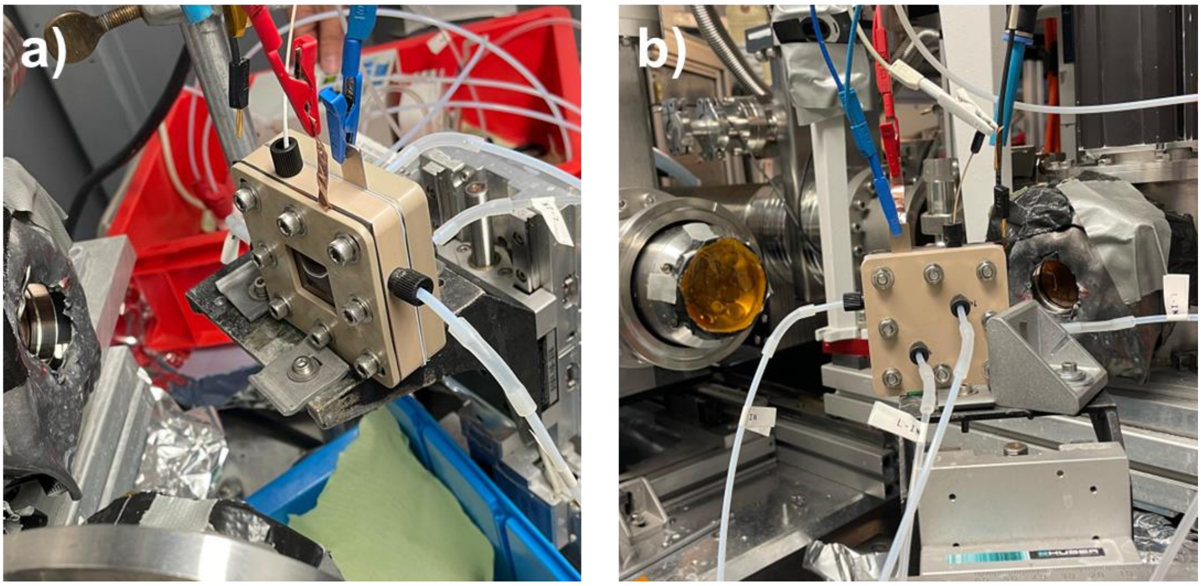

Figure S32. Photographs of the experimental set-up used for *in situ* XAS studies including the divided spectroelectrochemical (SEC) flow cell (three electrode arrangement).

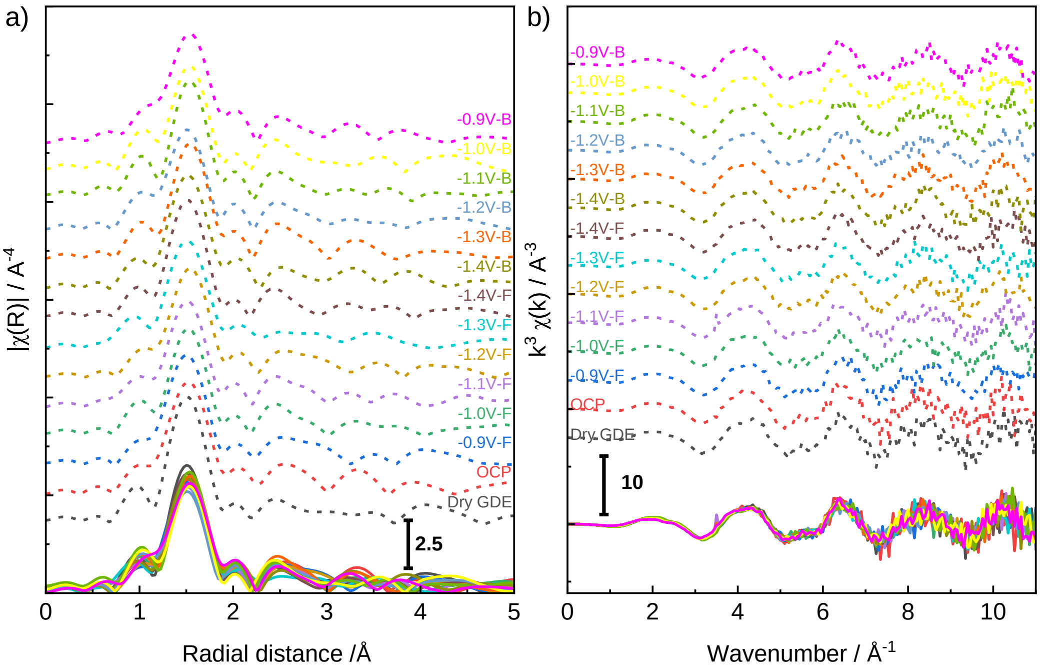

Figure S33. *In situ* XAS analysis of a **Cat3**-modified electrode. Fourier transform EXAFS spectra in R-space (a) and in K-space (b) for **Cat3** in dry form, under OCP conditions, and under CO_2RR conditions at -0.9 V, -1.0 V, -1.1 V, -1.2 V, -1.3 V and -1.4 V vs. RHE (aligning with our study in the H-cell). The corresponding XANES spectra are shown in the manuscript. Electrolyte: CO_2-saturated aq. 0.1 M $CsHCO_3$.

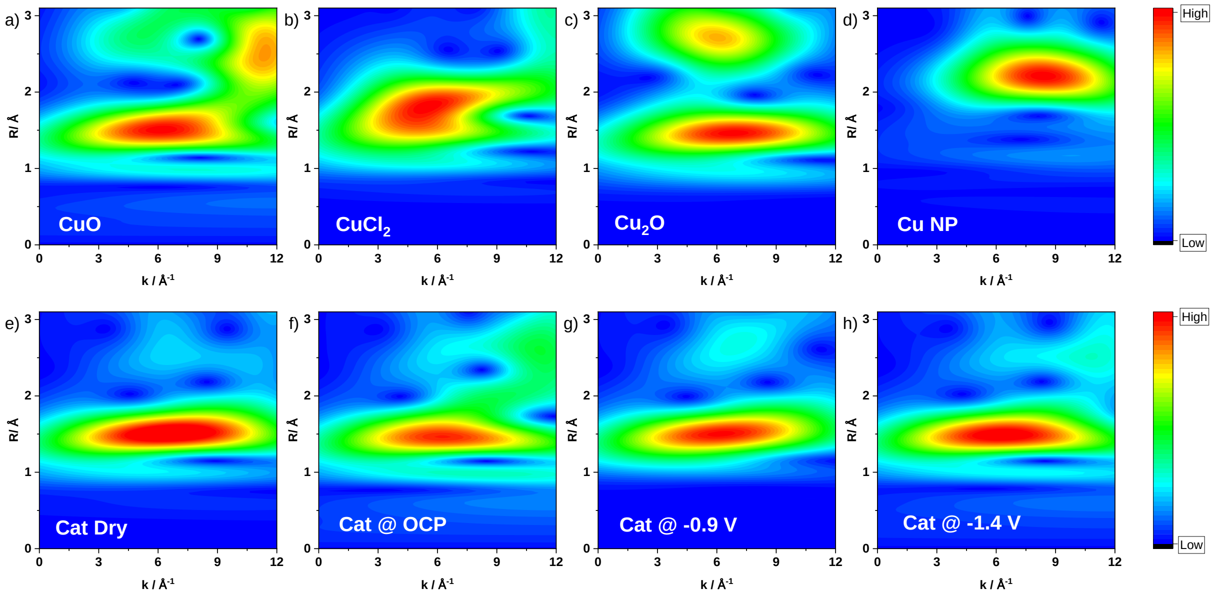

Figure S34. a-d) Wavelet-transformed EXAFS (WT-EXAFS) results for reference samples including CuO, $CuCl_2$, Cu_2O and Cu nanoparticles (NP). e-f) WT-EXAFS analysis of a **Cat3**-modified electrode in the dry state, under OCP conditions, and under CO_2RR conditions at -0.9 V and -1.4 V, respectively. Electrolyte: CO_2-saturated aq. 0.1 M $CsHCO_3$.

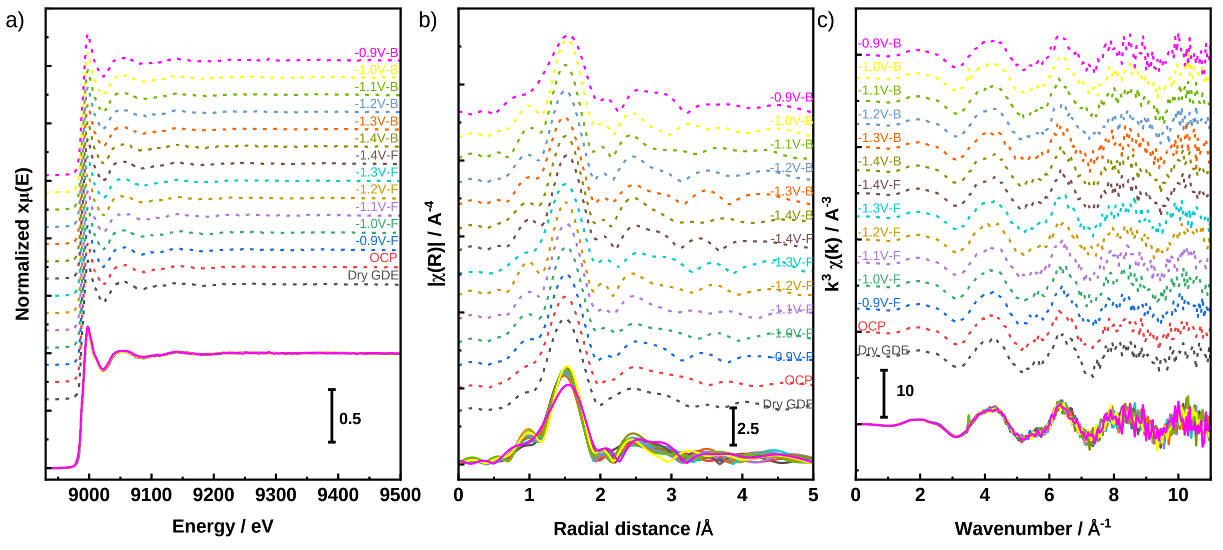

Figure S35. Control experiments: *In situ* XAS analysis of a **Cat3**-modified electrode. Normalized Cu K-edge XANES spectra (a), Fourier transform EXAFS spectra in R-space (b) and in K-space (c) for the dry state, under OCP conditions, and under CO_2RR conditions at -0.9 V, -1.0 V, -1.1 V, -1.2 V, -1.3 V and -1.4 V vs. RHE, respectively (aligning with our H-cell studies). Electrolyte: CO_2-saturated aq. 0.1 M $KHCO_3$.

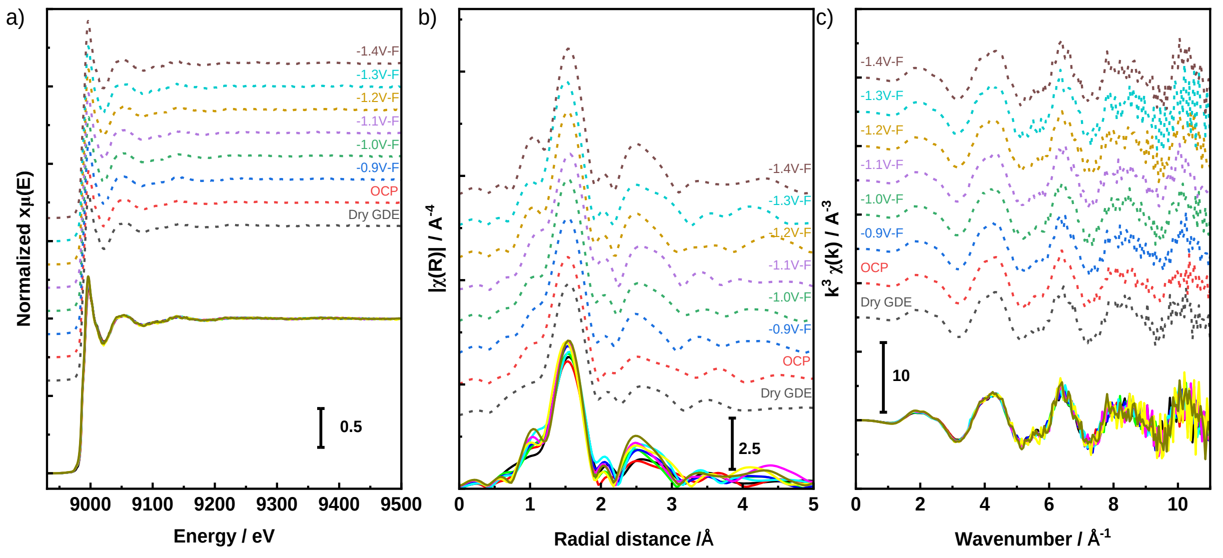

Figure S36. Control experiments: *In situ* XAS analysis of a **Cat3**-modified electrode. Normalized Cu K-edge XANES spectra (a), Fourier transform EXAFS spectra in R-space (b) and in K-space (c) for the dry state, under OCP conditions, and under CO_2RR conditions at -0.9 V, -1.0 V, -1.1 V, -1.2 V, -1.3 V and -1.4 V vs. RHE, respectively (aligning with our H-cell studies). Electrolyte: N_2-saturated aq. 0.1 M $CsHCO_3$.

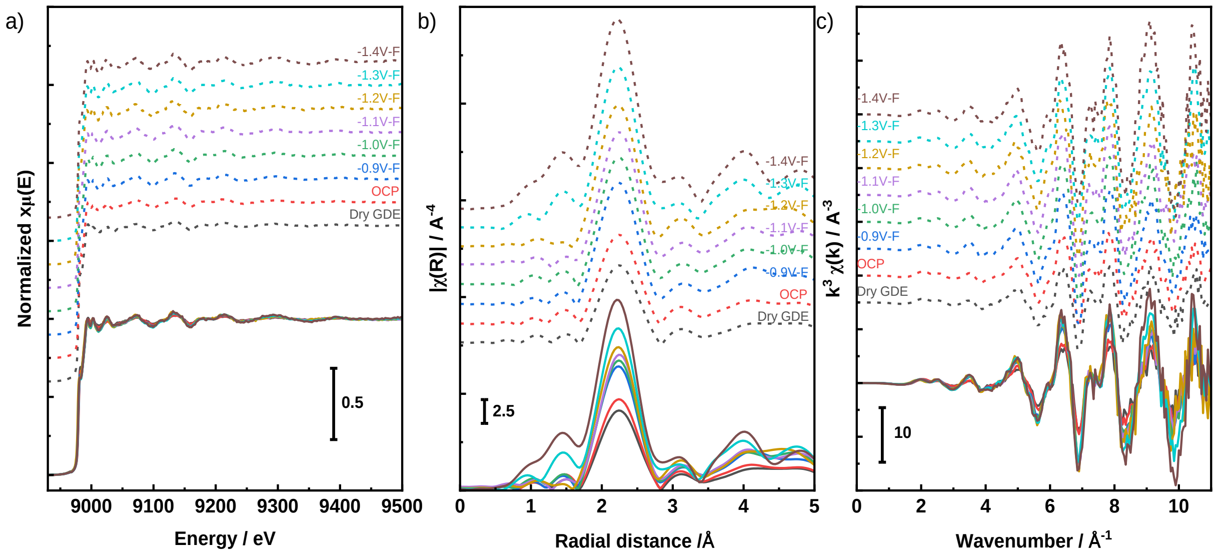

Figure S37. Control experiments: *In situ* XAS analysis of a **Cu NP**-modified electrode. Normalized Cu K-edge XANES spectra (a), Fourier transform EXAFS spectra in R-space (b) and in K-space (c) for the dry state, under OCP conditions, and under CO_2RR conditions at -0.9 V, -1.0 V, -1.1 V, -1.2 V, -1.3 V and -1.4 V vs. RHE, respectively (aligning with our H-cell studies). Electrolyte: CO_2-saturated aq. 0.1 M $CsHCO_3$.

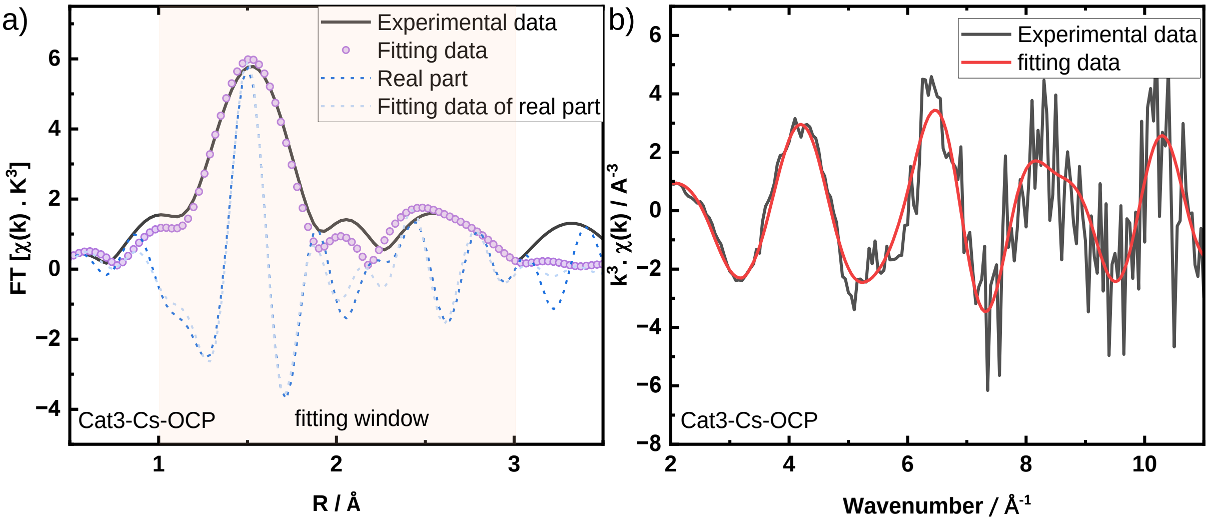

Figure S38. Fitting of experimental XAS data. Fourier transform EXAFS in R-space (a) and in K-space (b) for a **Cat3-**modified electrode under OCP conditions in CO_2-saturated aq. 0.1 M $CsHCO_3$.

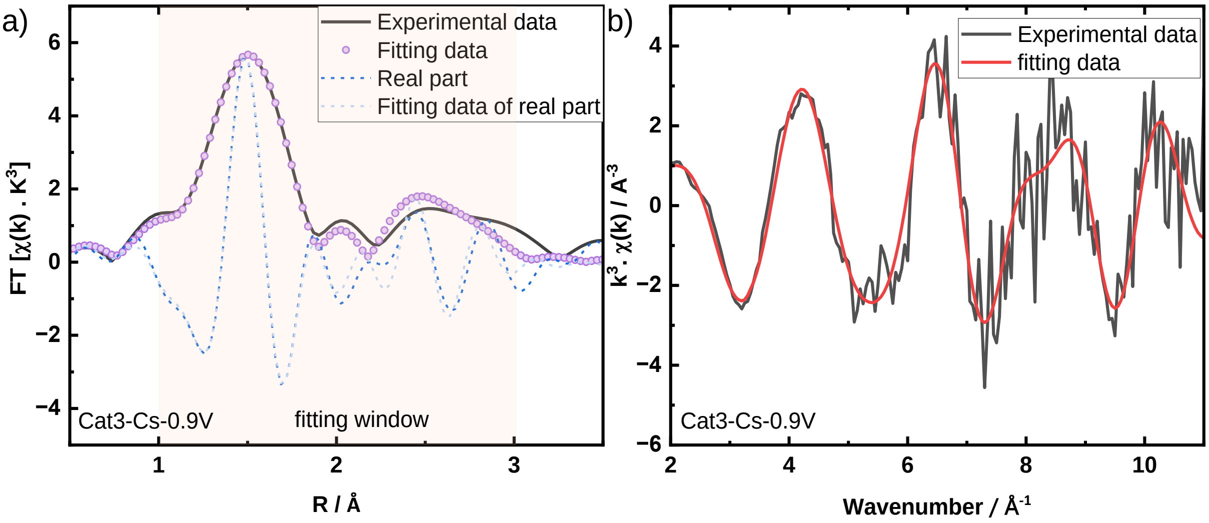

Figure S39. Fitting of experimental XAS data. Fourier transform EXAFS in R-space (a) and in K-space (b) for a **Cat3**-modified electrode at -0.9 V vs. RHE in CO_2-saturated aq. 0.1 M $CsHCO_3$.

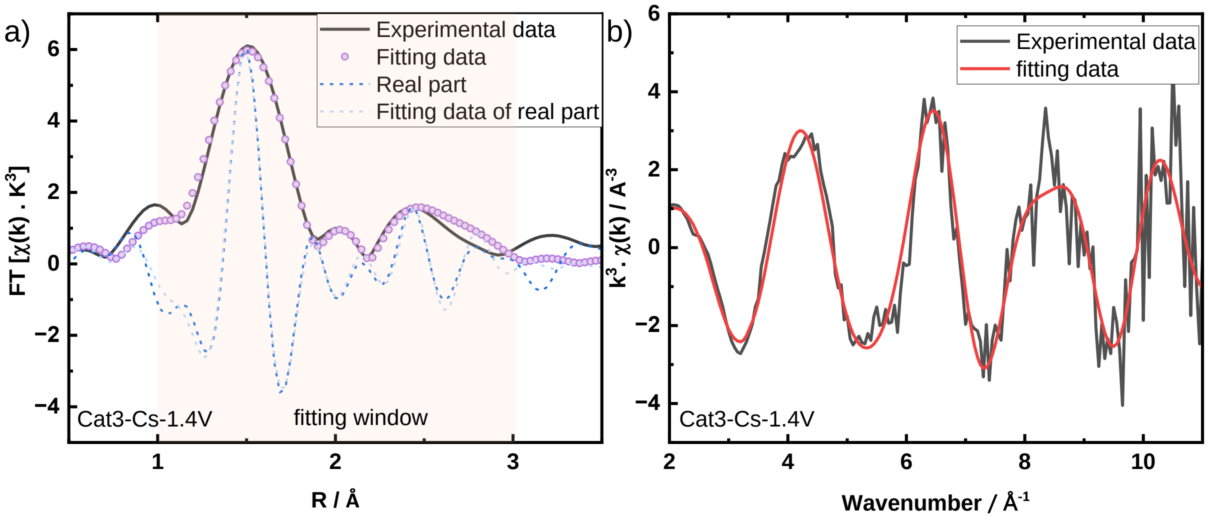

Figure S40. Fitting of experimental XAS data. Fourier transform EXAFS in R-space (a) and in K-space (b) for a **Cat3**-modified electrode at -1.4 V vs. RHE in CO_2-saturated aq. 0.1 M $CsHCO_3$.

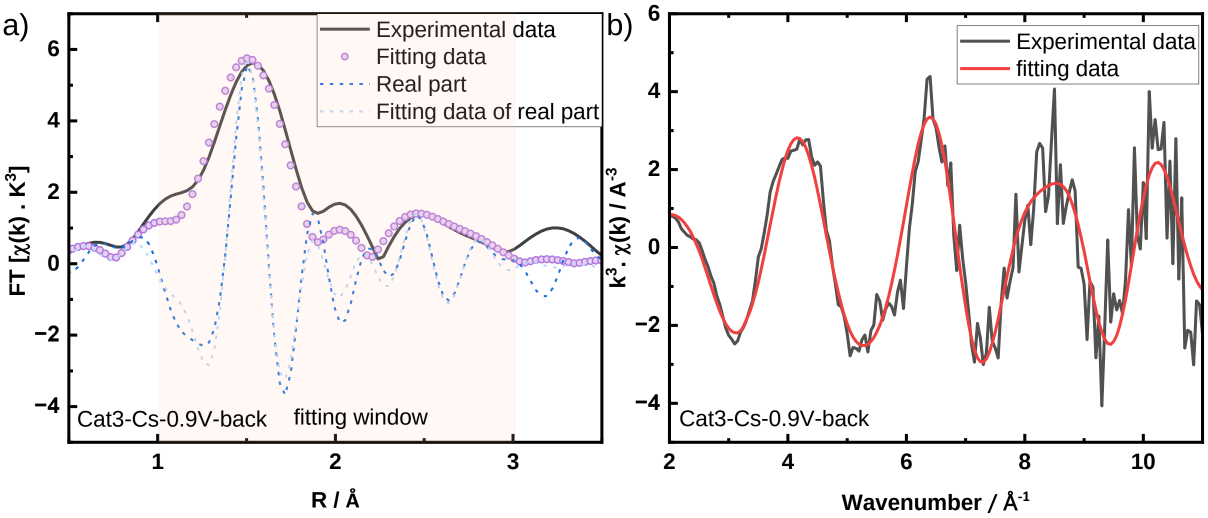

Figure S41. Fitting of experimental XAS data. Fourier transform EXAFS in R-space (a) and in K-space (b) for a **Cat3**-modified electrode at -0.9 V vs. RHE in CO_2-saturated aq. 0.1 M $CsHCO_3$ (backward potential scan).

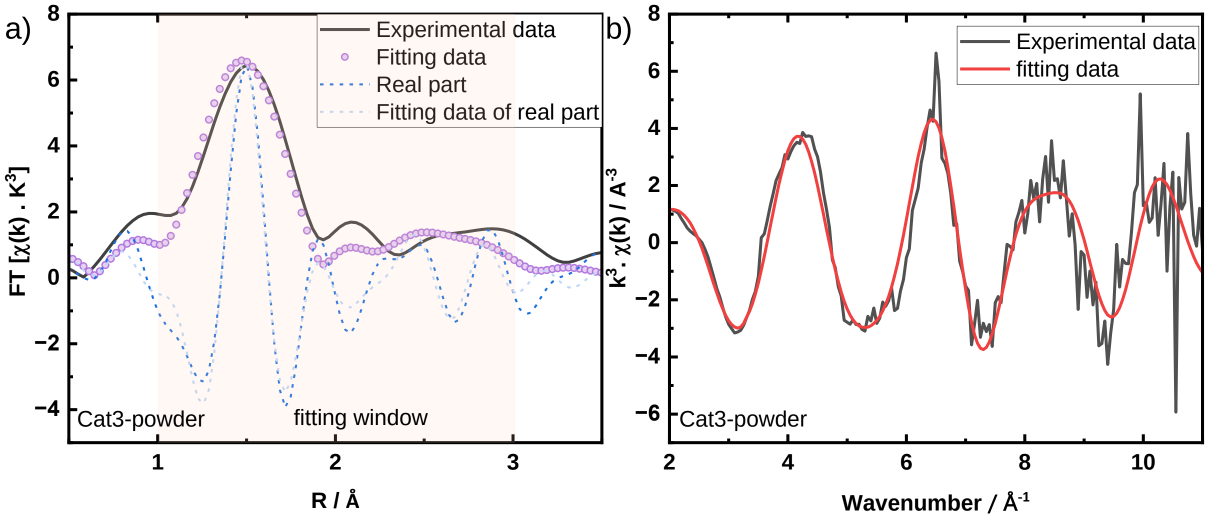

Figure S42. Fitting of experimental XAS data. Fourier transform EXAFS in R-space (a) and in K-space (b) for **Cat3** powder (from *ex situ* measurements).

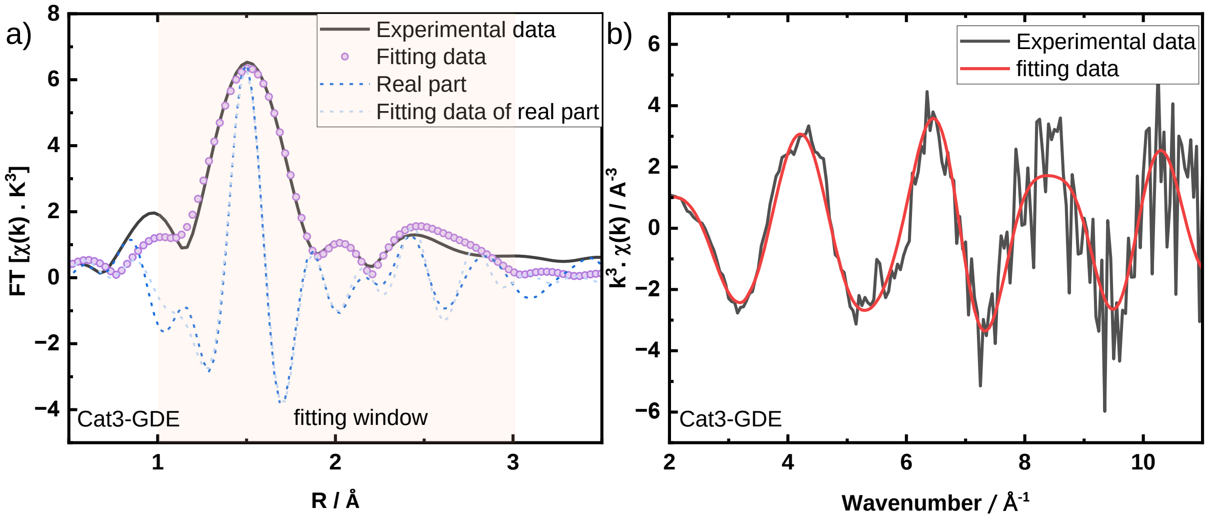

Figure S43. Fitting of experimental XAS data. Fourier transform EXAFS in R-space (a) and in K-space (b) for a freshly prepared **Cat3-**modified GDE electrode (from *ex situ* measurements).

4. Characterization of Cat3 in the MEA environment

4.1. Electrochemical studies in a MEA electrolyzer

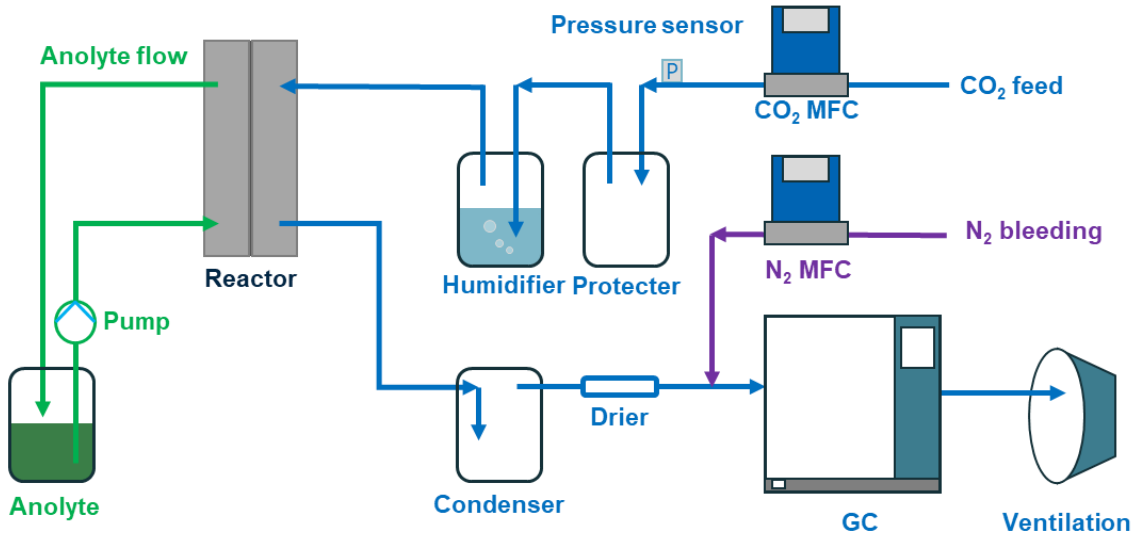

Figure S44. Schematic illustration of the experimental set-up used for CO_2RR studies under MEA conditions.

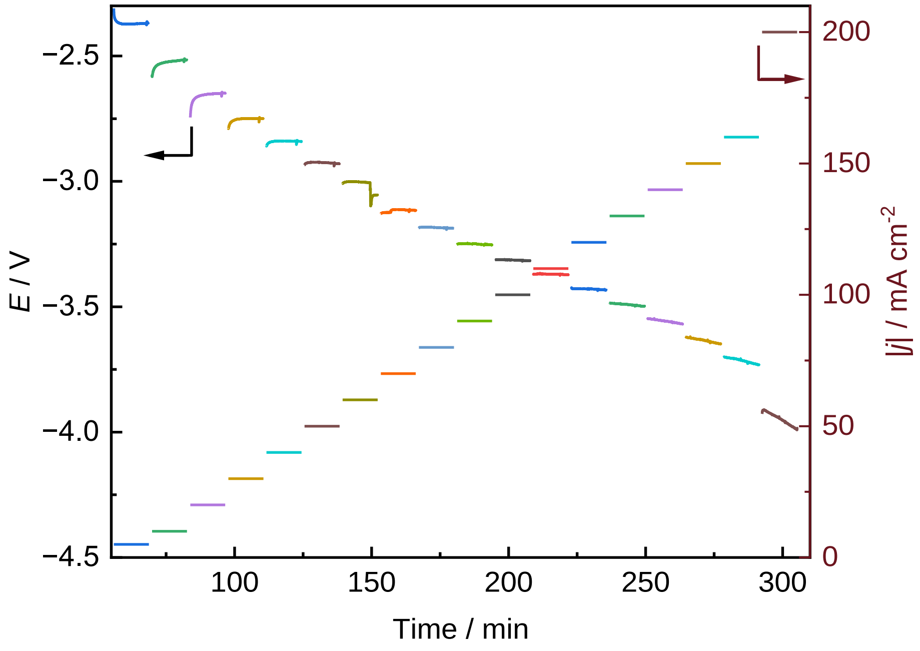

Figure S45. Electrochemical reduction of CO_2 in MEA electrolyzer using a **Cat3**-modified carbon GDE at different current densities (0.1 M $CsHCO_3$ as anolyte).

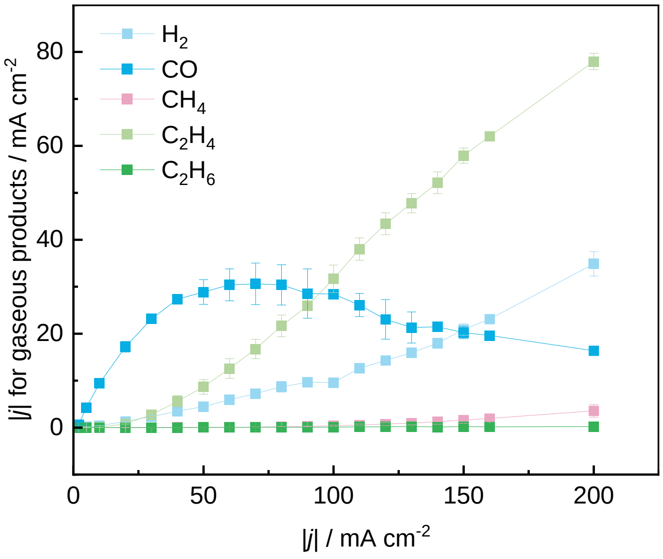

Figure S46. Partial current densities for e-CO_2RR (gaseous products) in a MEA electrolyzer using a **Cat3**-modified carbon GDE at different current densities (0.1 M $CsHCO_3$ as anolyte).

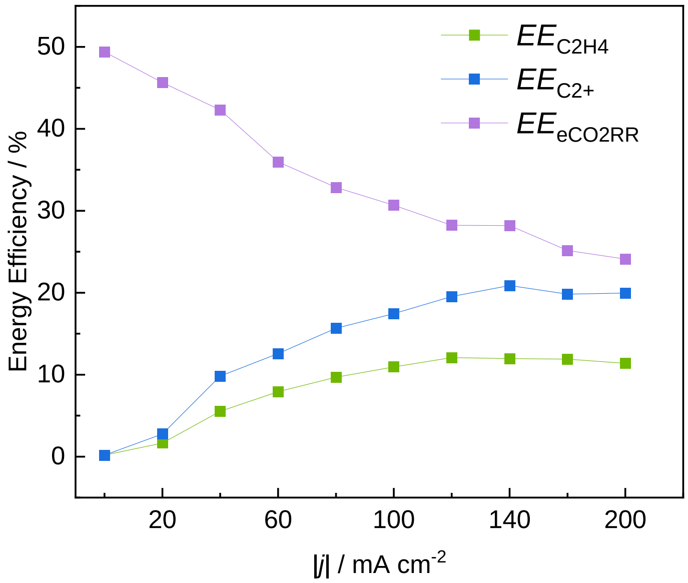

Figure S47. Energy efficiency (*EE*) with respect to formation of C_2H_4 , C_{2+} products, and CO_2 reduction taking into account all detected products formed, analyzed at different current densities using a **Cat3-** modified GDE in the MEA electrolyzer (for conditions, see the standard protocol in the Experimental Section).

The energy efficiency (*EE*) with respect to formation of different products (*i*) was calculated using the following equation,

$$EE = \frac{\left(1.23\,V - E_i^0\right) * FE_i}{U_{Cell}} * 100\%$$

wherein the themodynamic potential of the anodic reaction (oxygen evolution) is 1.23 V, E_i^0 is the thermodynamic potential for the formation of a specific product, FE_i the product-specific Faradaic

efficiency, and U_{cell} the applied cell voltage including Ohmic losses. For EE_i calculations, the standard potentials (E_i^0) for the different products were adopted from the literature,[15] with the specific values listed below in V vs. RHE:

**Product**	CO	CH_4	HCOOH	C_2H_4	C_2H_6	C_2H_5OH	CH_3COOH	C_3H_7OH
$\boldsymbol{E_i^0}$	-0.1	0.17	-0.12	0.08	0.14	0.09	0.11	0.1

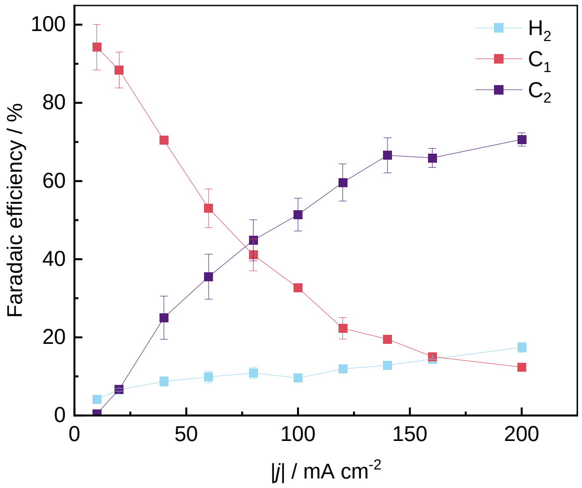

Figure S48. Comparison of the FEs for H_2, C_1 and C_2 products (MEA electrolyzer, 0.1 M $CsHCO_3$ as the anolyte).

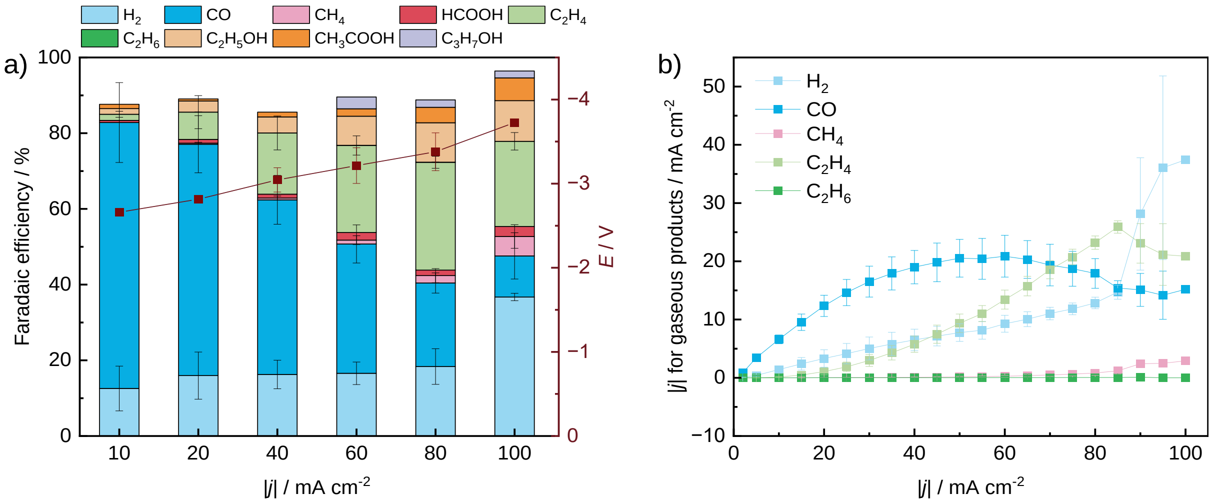

Figure S49. Reduction of CO_2 in a MEA electrolyzer using a **Cat3**-modified carbon GDE at different current densities. a) Product distributions and average cell voltage at different current densities. b) Partial current densities for different gaseous products at varying current density (0.1 M $KHCO_3$ as anolyte).

[15] I. Chorkendorff et al, *Chem. Rev.* **2019**, *119*, 7610–7672.

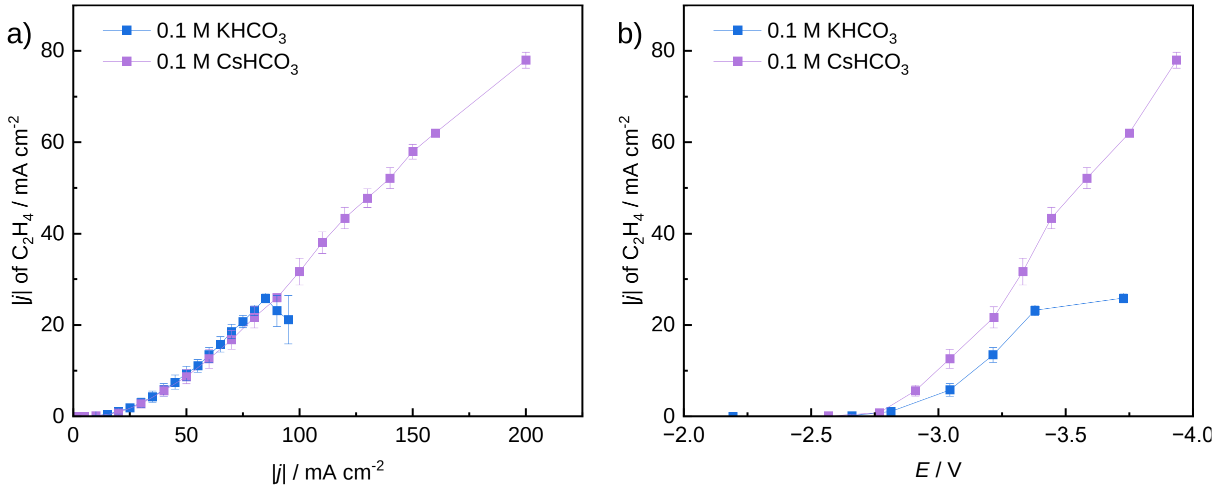

Figure S50. Comparison between the results obtained with a MEA electrolyzer using 0.1 M $CsHCO_3$ and 0.1 M $KHCO_3$ as the anolyte.

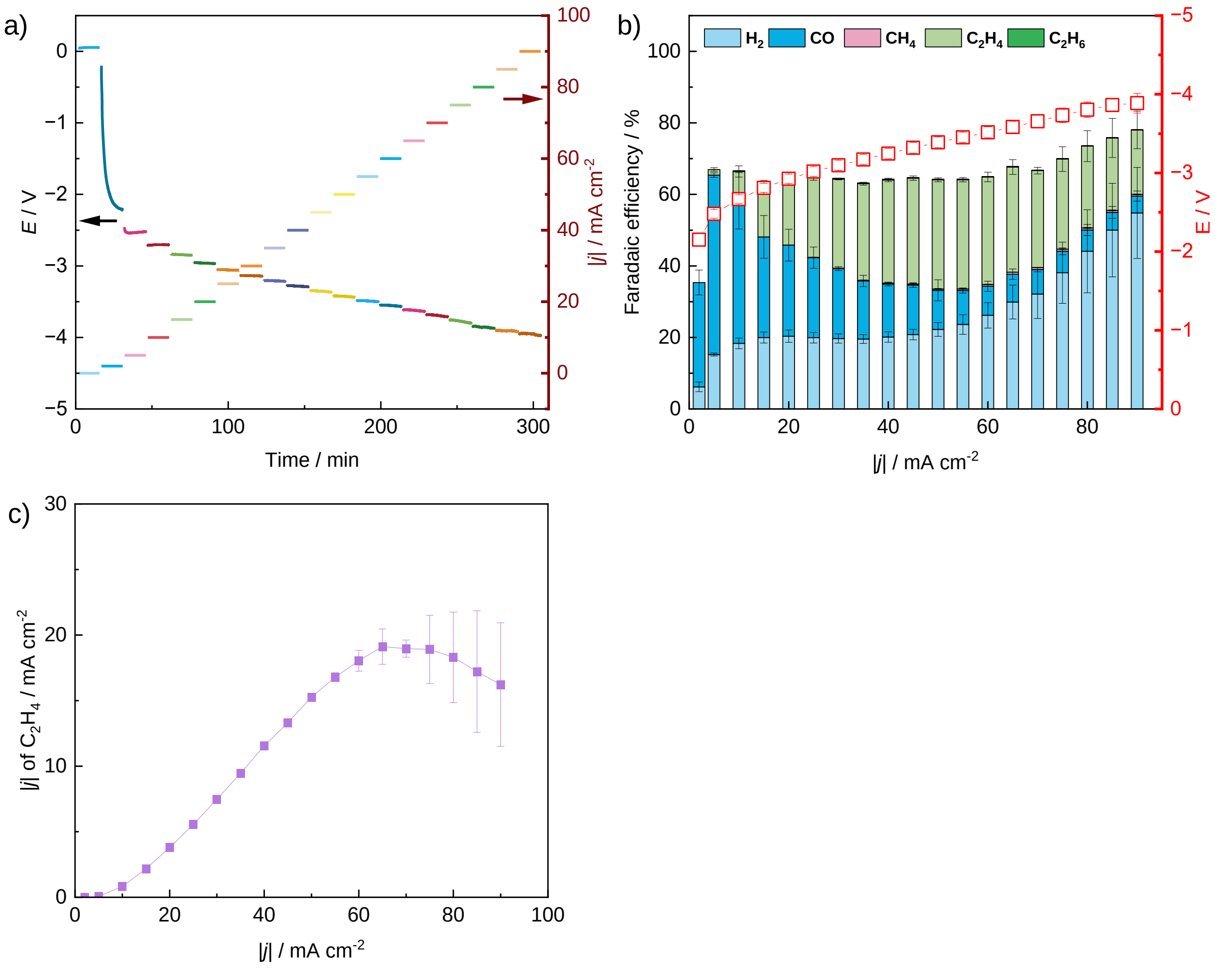

Figure S51. a) Electrochemical reduction of CO_2 in a MEA electrolyzer using a **Cat2**-modified carbon GDE (1 mg cm^{-2}, same mass loading as described for **Cat3**-modified electrodes) at different current densities. b) Gaseous product distributions and average cell voltage at different current densities. c) Partial current densities for C_2H_4 at varying current density (0.1 M $CsHCO_3$ as analyte).

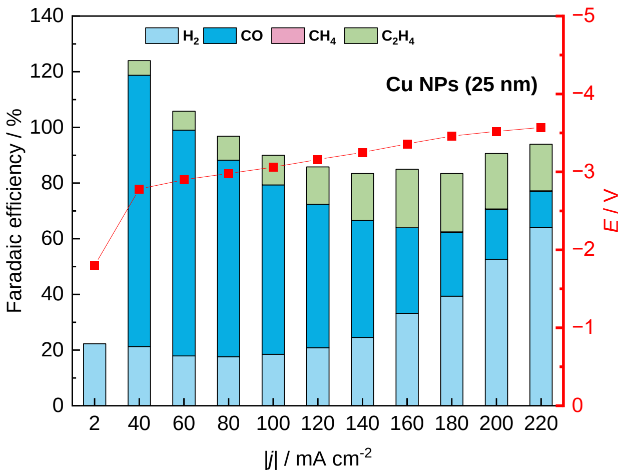

Figure S52. Results of control experiments using a carbon paper GDE loaded with Cu NPs (25 nm). For preparation of the electrode, the standard procedure was applied (mass loading: 1 mg cm^{-2} Cu NPs).

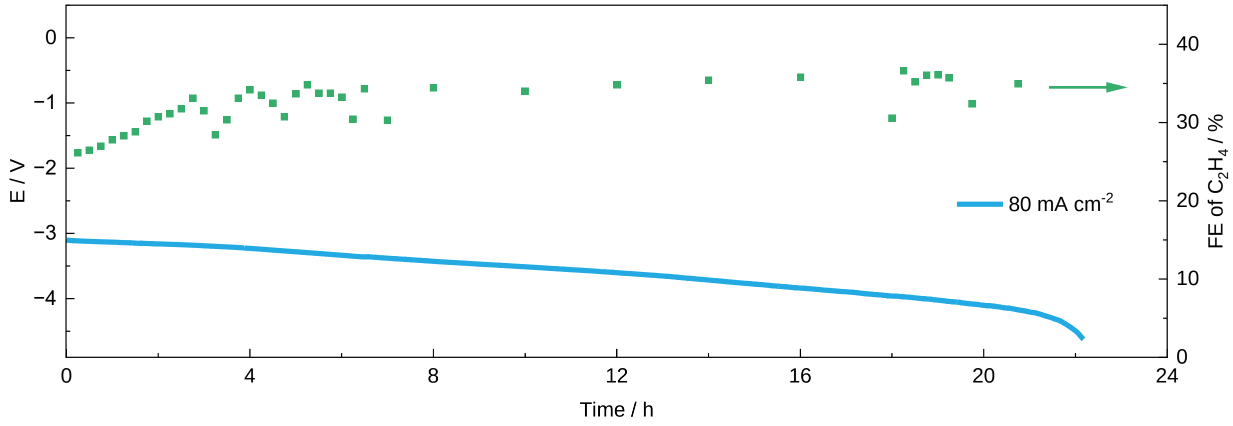

Figure S53. Long-term study of a **Cat3**-modified GDE at –80 mA cm^{-2} (0.1 M $CsHCO_3$ as the anolyte).

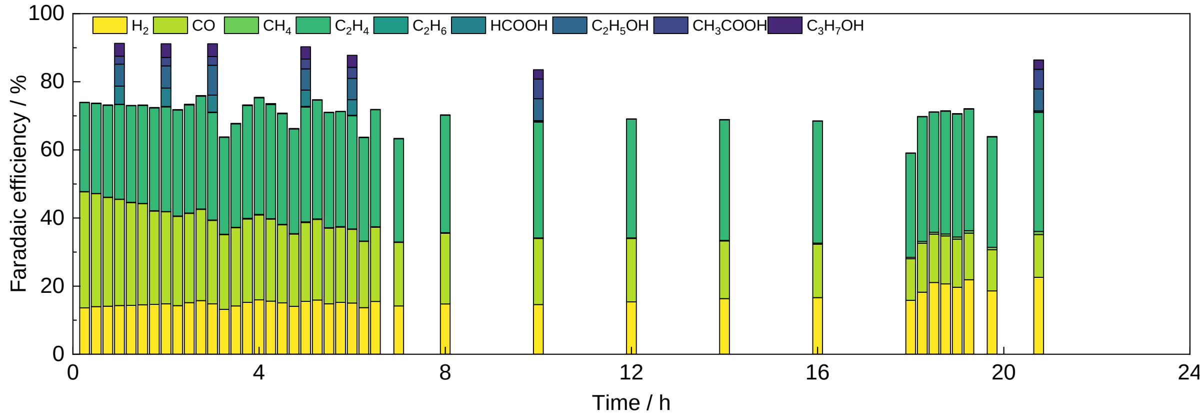

Figure S54. Progression of the product distribution during long-term electrolysis using a **Cat3**-modified carbon GDE (for details of conditions, see Figure S53).

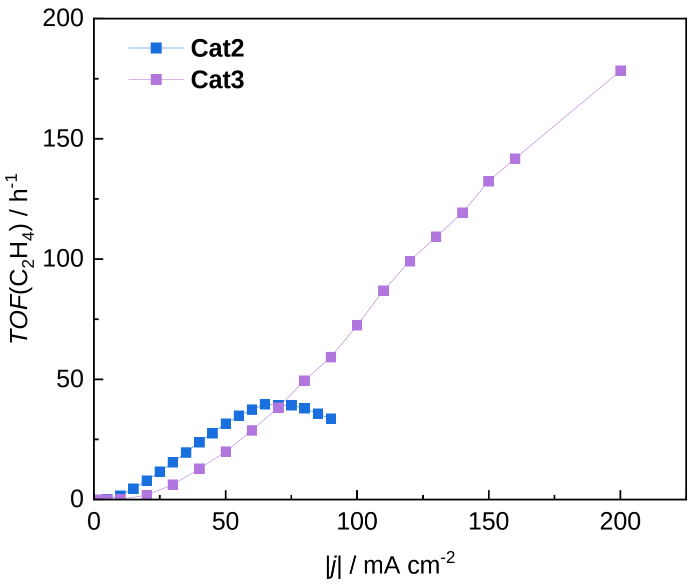

Figure S55. Plot of the *TOF* values for ethylene generation vs. the current density assuming two hydroxo-bridged Cu atoms as the active site (for details and assumptions with respect to *TOF* estimation, see below).

Estimation of *TOF* values for the plots shown in Figure S55 were carried out as follows: Assuming two hydroxo-bridged Cu atoms as the active center, the turnover frequency (*TOF*) for ethylene formation was calculated using the following equation,

$$TOF(C_2H_4) = \frac{i \times FE(C_2H_4)}{z\,F\,n_{Cu-Cu}}$$

wherein i is the current during MEA measurements, $FE(C_2H_4)$ the Faradaic efficiency for C_2H_4, z to the number of electrons transferred for CO_2-to-C_2H_4 ($z = 12$), and n_{Cu-Cu} the amount of Cu-Cu units within the catalyst loading (7.5 and 6.8 µmol per electrode for **Cat2** and **Cat3**, respectively).[16]

[16] As the exact average molecular weight of **Cat3** is unknown, the *TOF* values can only be calculated using a realistic estimate. For this purpose, the molecular weight of the structure shown in Figure S1 with six chloride counterions is used, assuming four active Cu-Cu sites within the octamer.

4.2. Pre- and post-electrolysis characterization of Cat3-modified GDEs

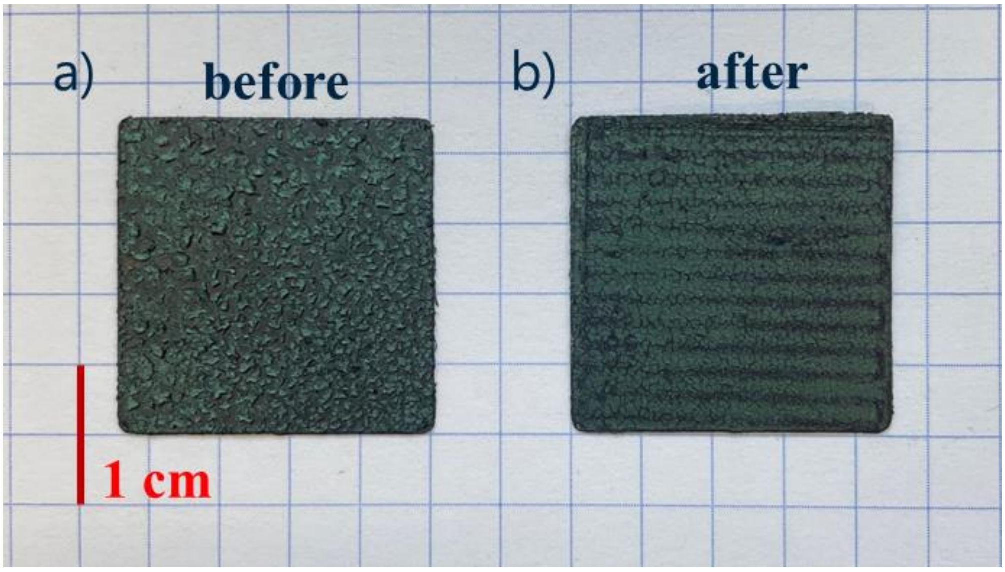

Figure S56. Photographs of a **Cat3**-coated GDE before (a) and after (b) electrolysis in a MEA set-up (for experimental details, see Figure S45).

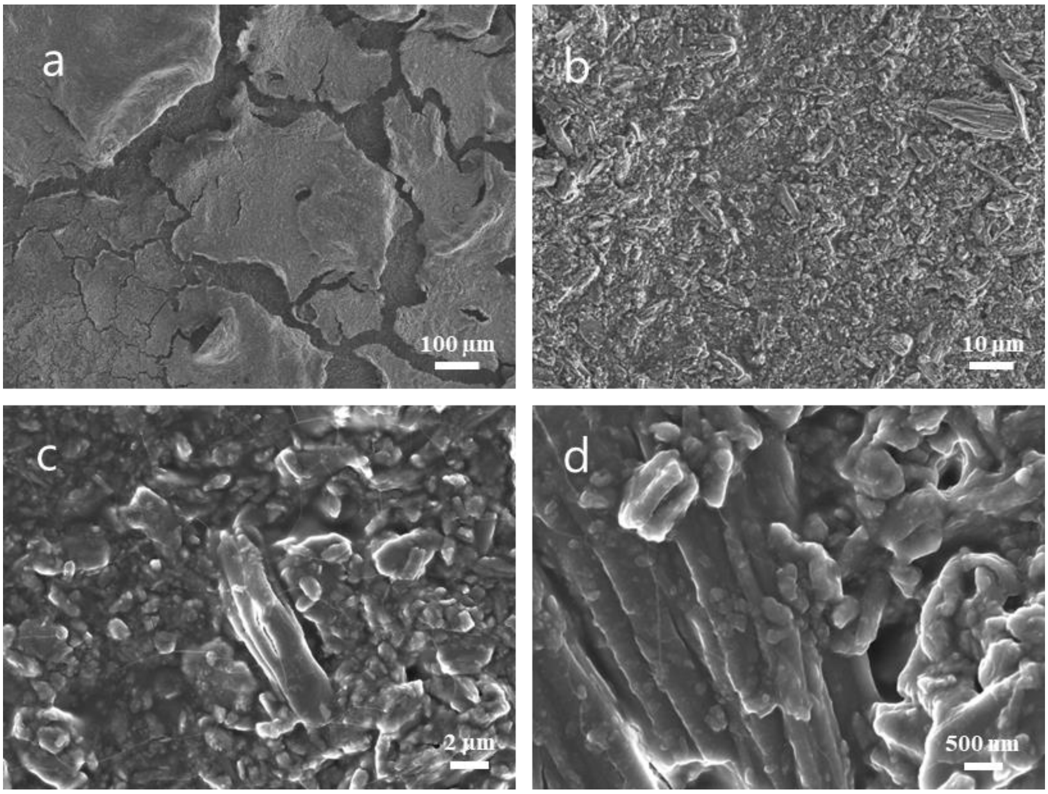

Figure S57. a-d) SEM images of a **Cat3**-modified carbon GDE before electrolysis in a MEA set-up (for details of electrode preparation, see the Experimental Section).

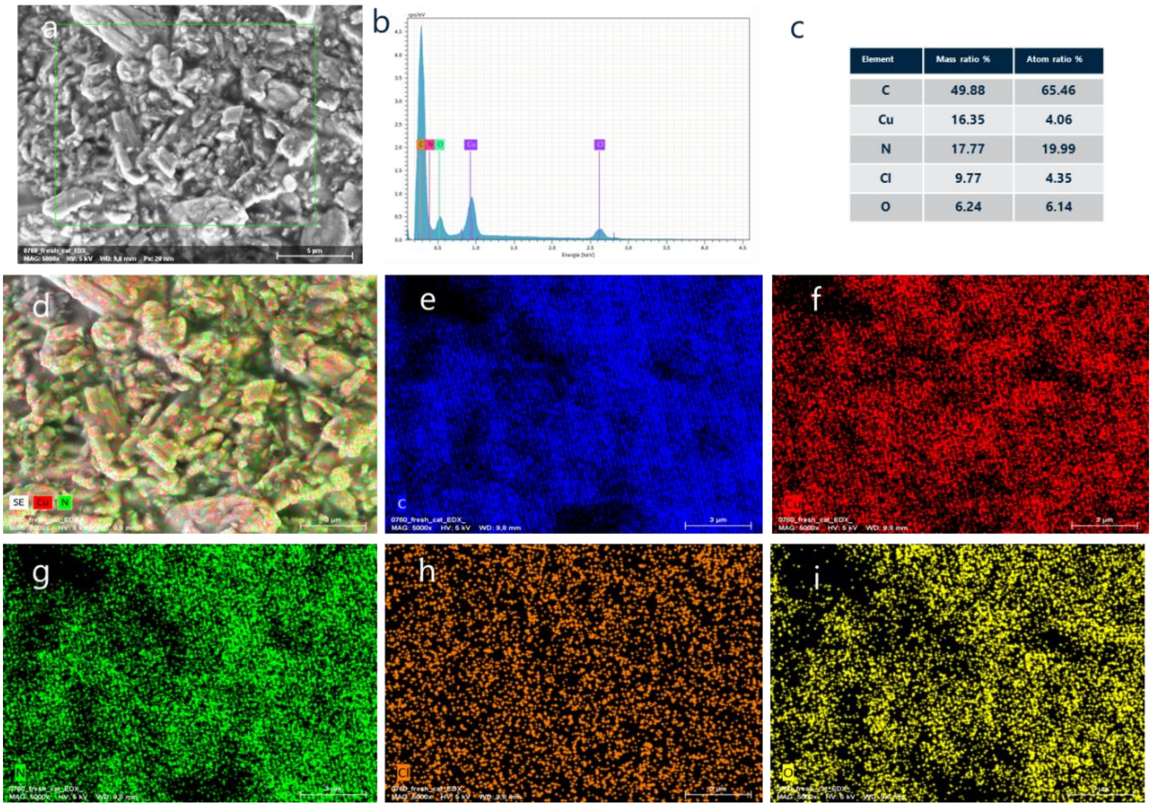

Element	Mass ratio %	Atom ratio %
C	49.88	65.46
Cu	16.35	4.06
N	17.77	19.99
Cl	9.77	4.35
O	6.24	6.14

Figure S58. a) SEM image of the same **Cat3**-modified GDE as shown in Figure S57 before electrolysis (for details of electrode preparation, see section the Experimental Section). b,c) Results of elemental analysis carried out in the domain indicated in a). d-i) EDX mapping for the relevant elements.

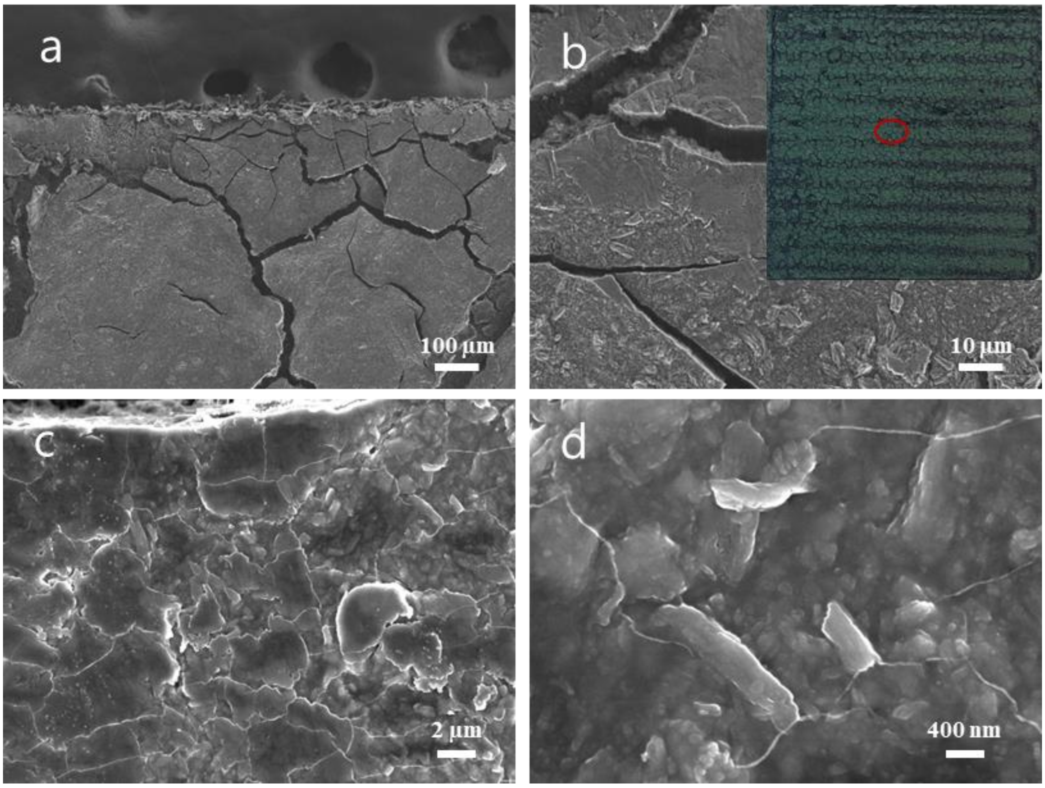

Figure S59. SEM images of a **Cat3**-modified carbon GDE after electrolysis process in Figure S45 in a MEA set-up (for details of sample preparation and electrolysis conditions, see the Experimental Section).

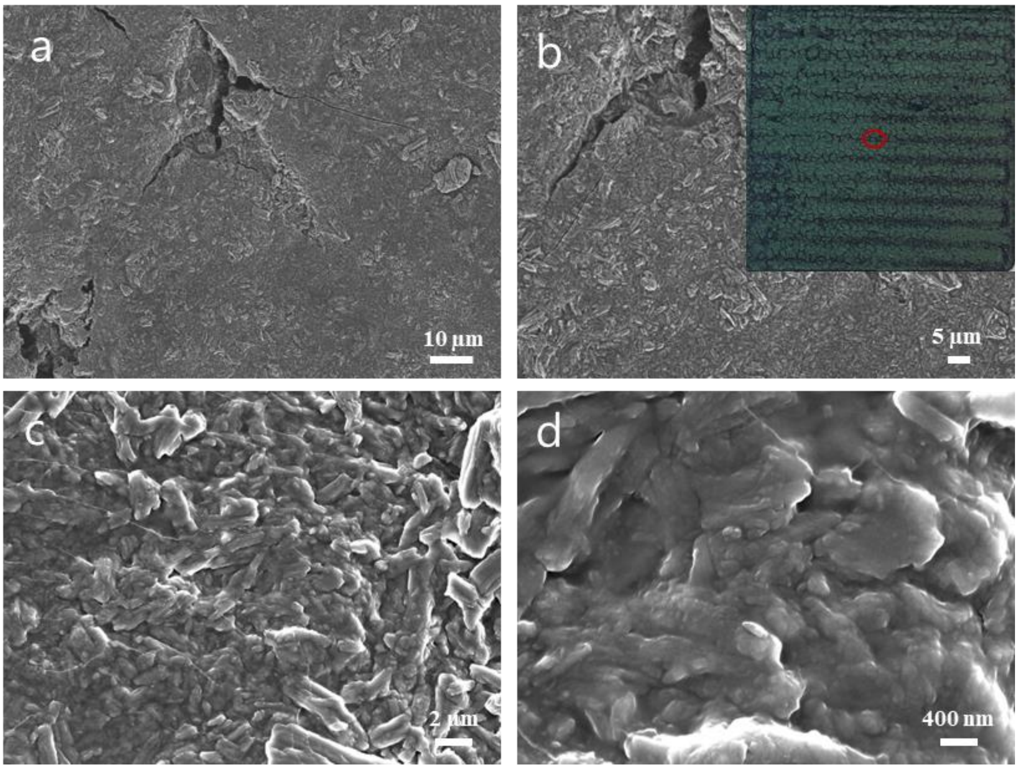

Figure S60. SEM images of a **Cat3**-modified carbon paper GDE after electrolysis process in Figure S42 in a MEA set-up (for details of sample preparation and electrolysis conditions, see the Experimental Section).

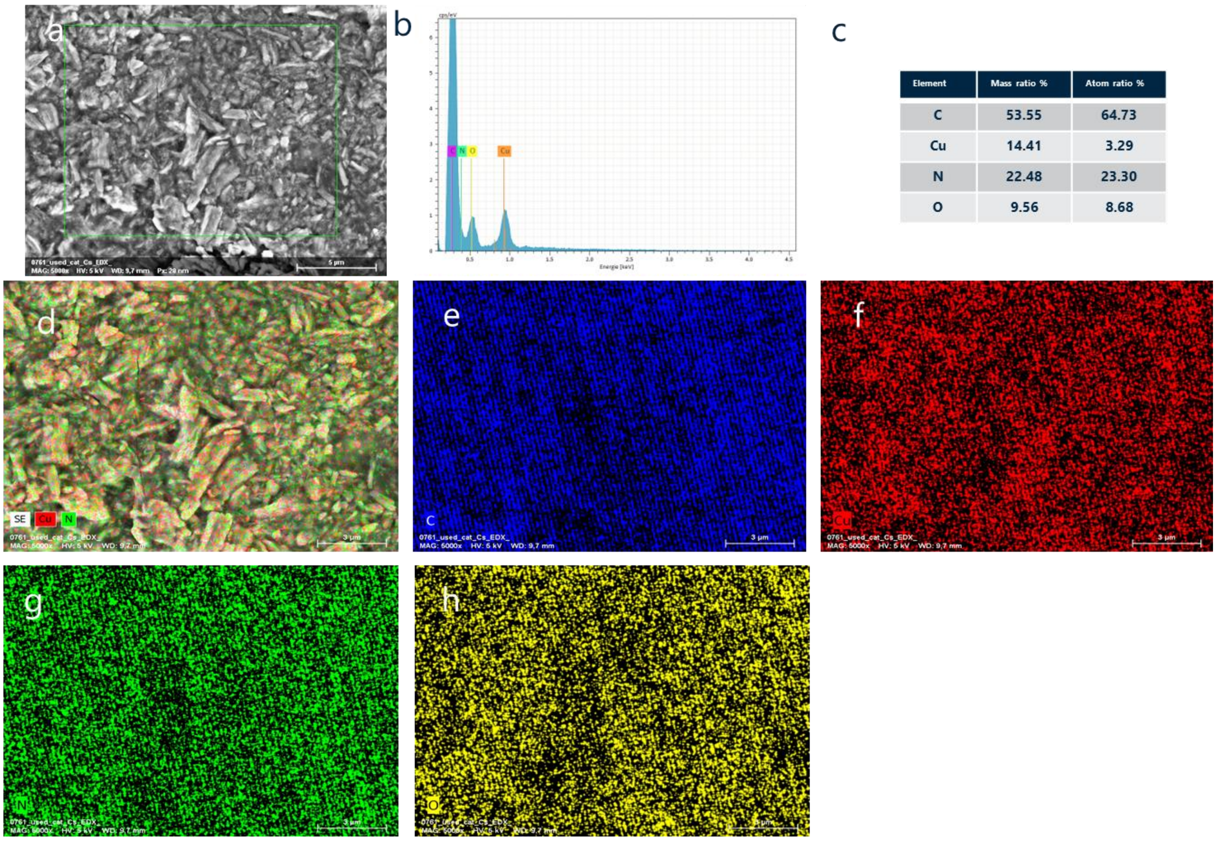

Element	Mass ratio %	Atom ratio %
C	53.55	64.73
Cu	14.41	3.29
N	22.48	23.30
O	9.56	8.68

Figure S61. a) SEM image of the same **Cat3**-modified GDE as shown in Figure S59 and Figure S60 after electrolysis. b,c) Results of elemental analysis carried out in the domain indicated in a). d-h) EDX mapping for the relevant elements.

The **Cat3**-modified GDE was investigated by STEM before and after electrolysis (Figure S62 and Figure S63). It should be noted that in principle, analysis of electrodes modified with molecular catalysts is complicated by the following issues: First, the electrode as authentic sample cannot be analyzed, but material must be removed from the surface and applied to a TEM grid. Second, the non-homogeneous structure of the electrode composed of catalyst and support is not easy to separate. Third, when scratching off the catalyst layer from the electrode (with pincer/razor blade) and subsequently immersing the powder into a solvent, the material can be changed. The scratched-off solid was therefore pestled and then dryly immobilized on the TEM grid. Only very little material could be immobilized in this manner, and Cu free parts were found that very likely belong to the carbon paper support. It should be noted that due to the inhomogeneity of the sample examined, the various particles may exhibit different affinities to the TEM grid.

In both samples, before and after electrolysis, Cu-containing domains were found, which exhibited distributed Cu (Figure S62 b and d) that appears partially enriched, comparable to a drop of coffee after drying. Besides, also a small fraction of CuO_x nanoparticles was found both in the fresh and used catalyst (Figure S62 a and c). However, based on the results of Raman spectroscopy, XAS, and XPS, it is safe to assume that the total fraction of CuO_x nanoparticles is very low (below the detection limit of the applied spectroscopic techniques).

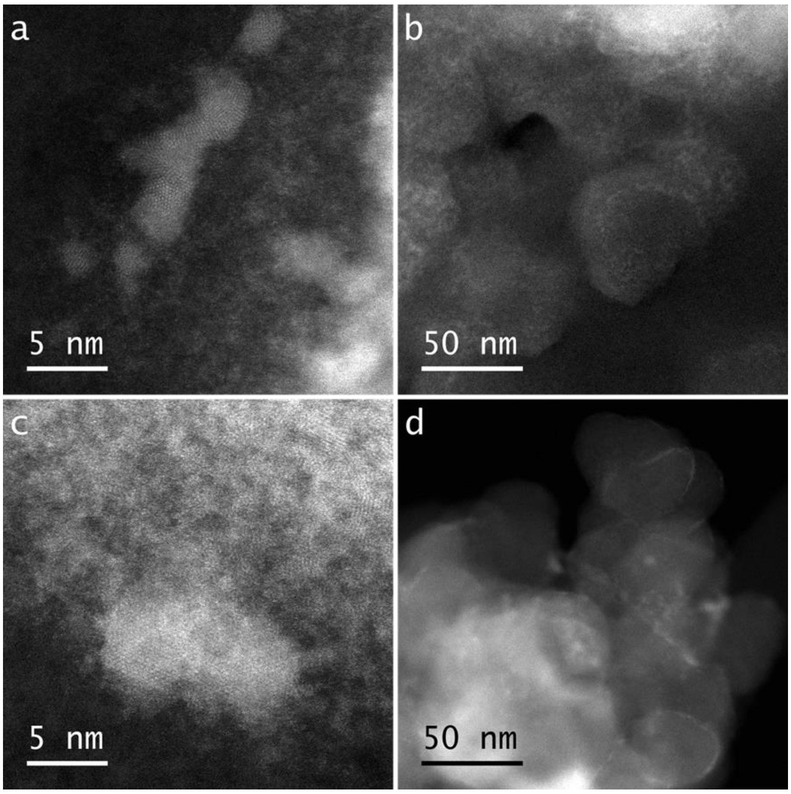

Figure S62. Selected STEM-HAADF images of **Cat3** immobilized with Sustainion XA-9 on a GDL before (a, b) and after (c, d) electrolysis. The materials were deposited dryly on the Ni grid after scratching from the electrode and pestling. For analysis of the results, see the discussion above.

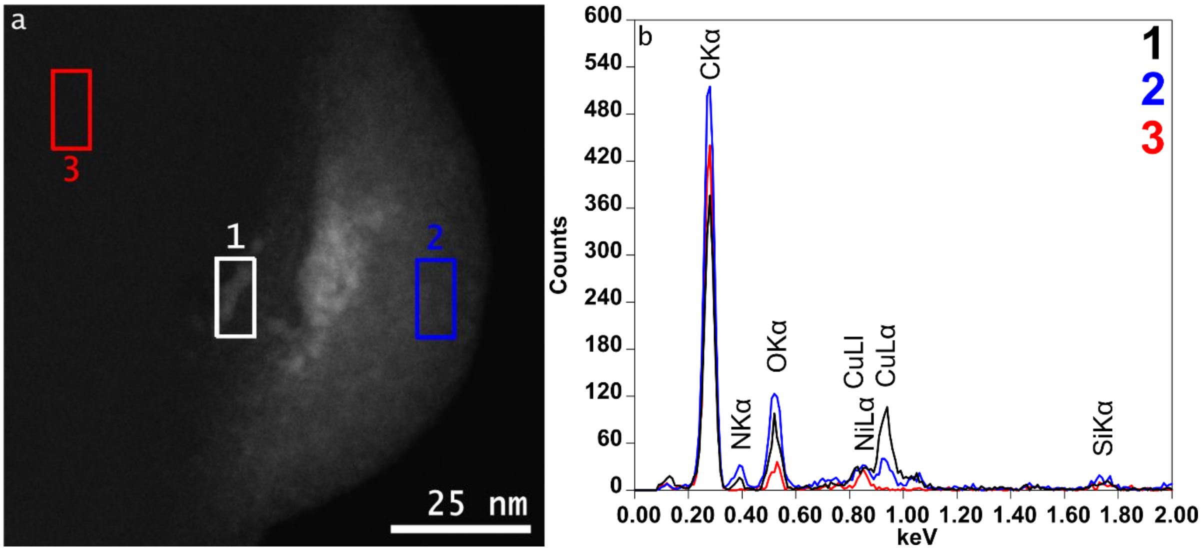

Figure S63. STEM-HAADF image (a) and selected EDX spectra (b) of a fresh **Cat3**-modified GDE, whereby the catalyst was immobilized with Sustainion XA-9 binder. Shown are the spectra of the regions highlighted in the STEM-HAADF image (a). Cu is clearly visible in spectra 1 and 2, while region 3 shows the spectrum of the carbon-supported Ni grid.

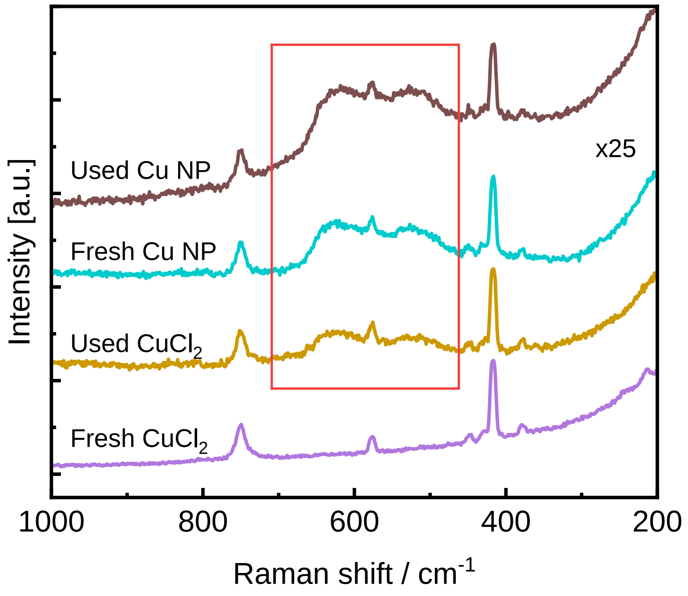

Figure S64. Raman spectra (785 nm excitation) of reference samples ($CuCl_2$ and Cu Nanoparticles (NP)) from our previous work.[6] Broad band features at ≈527 and 622 cm^{-1} in the rectangular area are assignable to Cu/CuO_x nanoparticles. Signals at 416, 576 and 750 cm^{-1} result from the used sapphire probe. Both the $CuCl_2$- and the Cu NP-modified carbon paper electrode was analyzed before and after electrolysis in the H-cell. Electrolyte: CO_2-saturated aq. 0.1 M $CsHCO_3$.

4.3. *In situ* XAS analysis using a SEC MEA cell

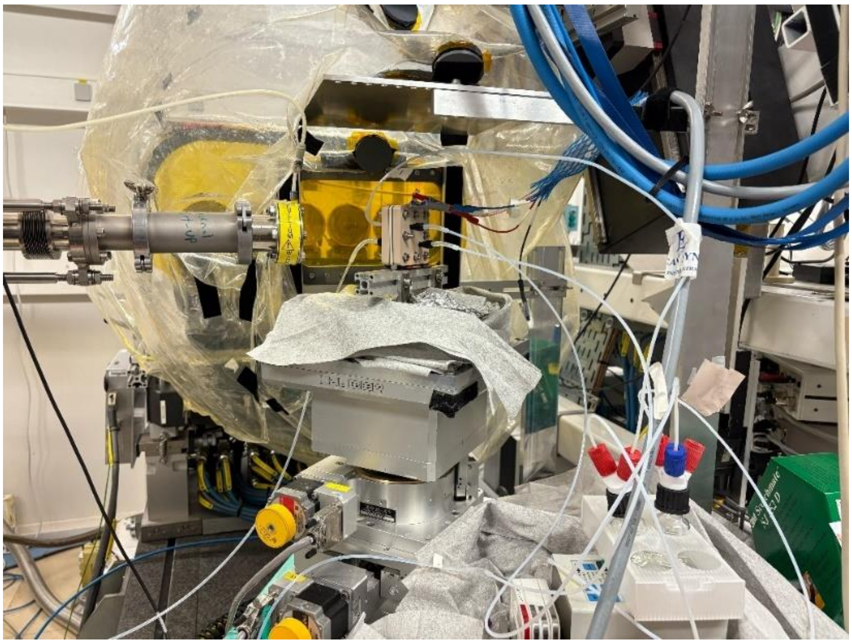

Figure S65. Experimental set-up used for *in situ* HERFD-XAS studies of **Cat3**-modified GDEs in the MEA environment.

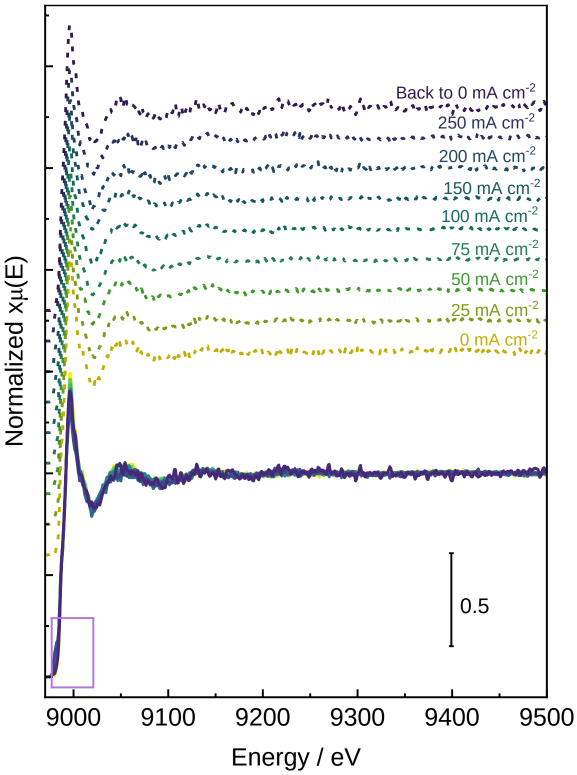

Figure S66. *In situ* HERFD-XAS analysis of a **Cat3-**modified carbon GDE under CO_2RR conditions at 0, 25, 50, 75, 100, 150, 200, 250 mA cm^{-2} and back to 0 mA cm^{-2} (aligning with our experiments in the MEA set-up, see Figure S45). Anolyte: 0.1 M $CsHCO_3$.

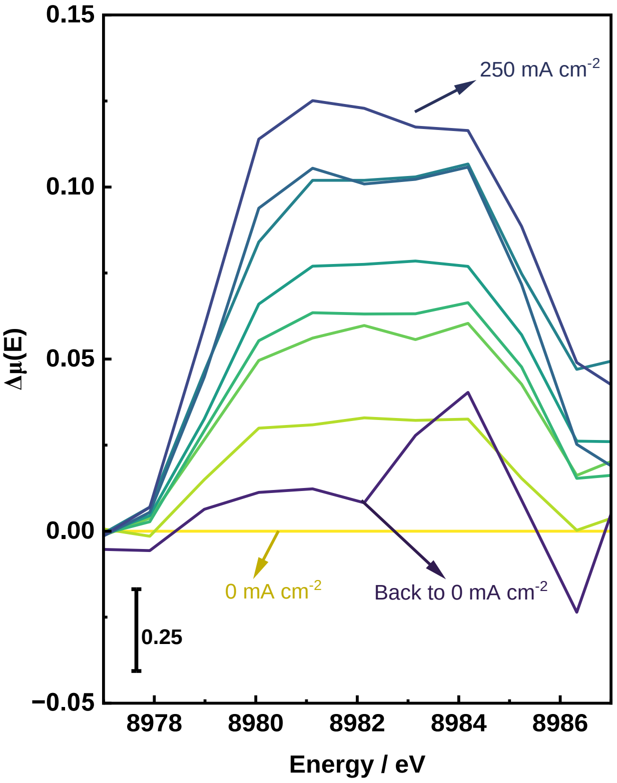

Figure S67. Magnification of the area highlighted in the *in situ* Cu K-edge Δμ(E) spectra of a **Cat3**-modified carbon GDE recorded at different current densities (see Figure 5d).

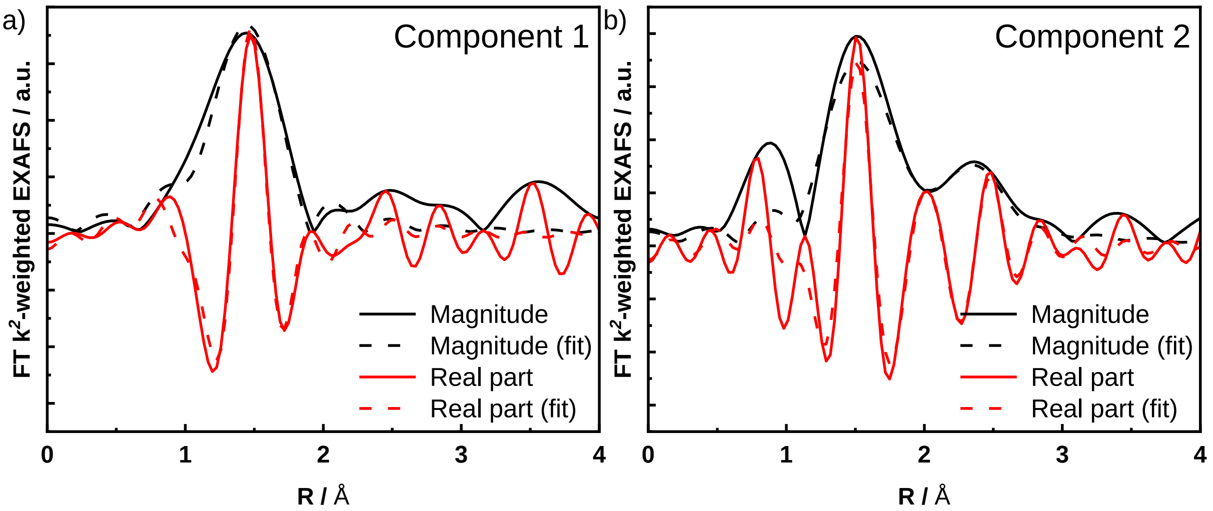

Figure S68. EXAFS fits of the most distinct components of the HERFD-XAS dataset resolved by MCR-ALS. For fitting details, see Table S6.

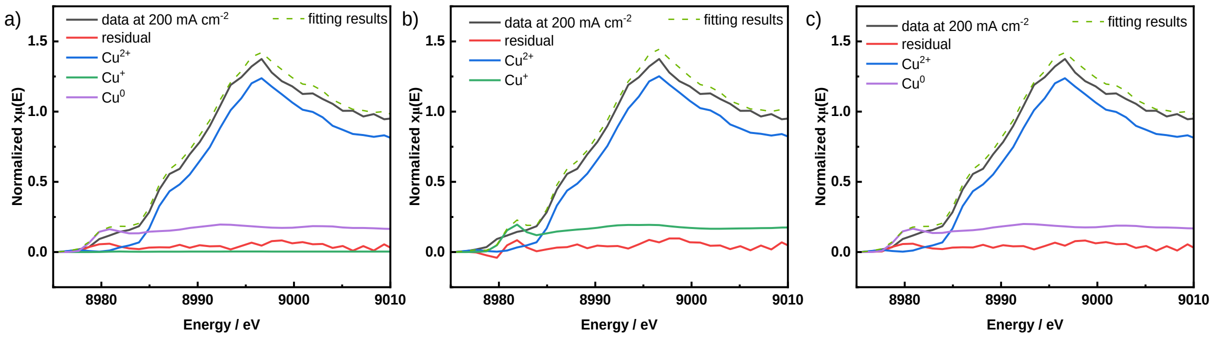

Figure S69. Linear combination fitting of results obtained during electrolysis at 200 mA cm^{-2} (anolyte: 0.1 M $CsHCO_3$) with a **Cat3**-modified carbon GDE by using different references (Cu^{2+} is related **Cat3-**modified electrode at OCV, Cu^{+} is Cu_2O and Cu^0 is Cu foil. For more information, see the Experimental Section and Table S7.

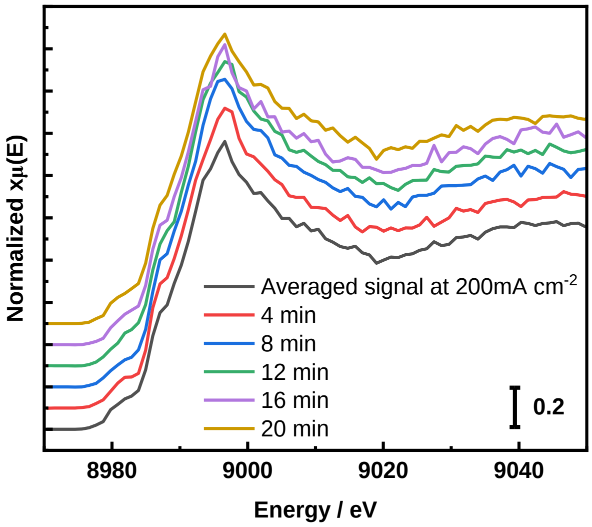

Figure S70. *In situ* HERFD-XAS analysis. XANES spectra of a **Cat3**-modified carbon GDE recorded at 200 mA cm^{-2} (anolyte: 0.1 M $CsHCO_3$).

Table S5. Structure parameters: Fitted coordination shell, coordination numbers (CN), disorder factors σ^2, interatomic distances (R), photoelectron reference energy correction $\Delta\varepsilon_0$, and R factors obtained from fitting of experimental Cu K-edge EXAFS data. Fitting details are presented below.

Experiment	Coord. shell	Coordination number (CN)	σ^2 / Å^2	R / Å	$\Delta\varepsilon_0$ / eV	R factor
**Cat3** powder	Cu-N/O<br>Cu-Cu	4.2±0.4<br>2.2±0.8	0.005±0.002<br>0.012	1.945±0.010<br>2.89±0.03	2.7±1.0	0.02
**Cat3** on GDE	Cu-N/O<br>Cu-Cu	3.1±0.2<br>0.9±0.3	0.003±0.001<br>0.007	1.945 ±0.006<br>2.89±0.02	3.8±0.6	0.01
**Cat3** at OCP (*in situ*)	Cu-N/O<br>Cu-Cu	3.0±0.2<br>1.1±0.3	0.003±0.001<br>0.007	1.949 ±0.007<br>2.88±0.02	3.8±0.8	0.01
**Cat3** at –0.9 V (*in situ*) [17]	Cu-N/O<br>Cu-Cu	3.2±0.2<br>1.2±0.3	0.004±0.001<br>0.007	1.939 ±0.007<br>2.90±0.02	3.6±0.8	0.01
**Cat3** at –1.4 V (*in situ*)[17]	Cu-N/O<br>Cu-Cu	3.2±0.2<br>1.0±0.2	0.004±0.001<br>0.007	1.942 ±0.006<br>2.89±0.02	3.7±0.6	0.01
**Cat3** at –0.9 V (*return*)[17]	Cu-N/O<br>Cu-Cu	3.1±0.3<br>0.9±0.4	0.003±0.001<br>0.007	1.946 ±0.009<br>2.90±0.03	2.3±1.0	0.02

The EXAFS spectra fitting of $k^3\chi(k)$ Fourier-transformed over a k-range of 2.0–11 Å^{-1} was conducted in R-space over a range of 1.0–3.0 Å, as described in the Experimental Section. Based on XANES, SC-XRD, XPS, and Raman spectroscopic results, contributions from Cu-Cu (metallic), Cu-O (metal oxides), and Cu-Cl paths were excluded. The structure was modeled using the binuclear Cu-phenanthroline complex from our previous work (CCDC:190413). In this model, a Cu-N/O path at ~1.95 Å and a Cu-Cu dimer path at ~2.9 Å represent the primary and secondary coordination shells, respectively. Refined parameters included coordination number (CN), bond length (R), and disorder factor (σ^2), along with photoelectron reference energy correction ($\Delta\varepsilon_0$). The amplitude reduction factor (S_0^2) was estimated at 0.78, based on the measured reference Cu foil standard with CN = 12.

Figure S38 – Figure S43 and Table S5 summarize the results, showing strong experimental-model alignment and low R factors, validating the fitting models. Distinguishing contributions from neighboring elements (N, O, and C) remains challenging, though interatomic distances provide valuable insight. Table S5 reveals slight differences in first-shell bond lengths between **Cat3** powder and the referenced Cu-phenanthroline complex.[6] It is noteworthy that after being spray-coated on GDE, the coordination numbers for both Cu-N/O and Cu-Cu decreased, likely attributed to the catalyst ink/layer preparation (sonicated in propanol with 15 wt% Nafion ionomer and dried at 50 °C to 1 mg cm^{-2} loading on the GDE support).

The bond lengths of Cu-N/O and Cu-Cu dimer interactions (~1.95 and ~2.9 Å, respectively) remained stable across powder, dried GDE (after spray-coating with **Cat3** ink), and under eCO_2RR conditions, with the Cu-nearest neighbor bond consistently shorter than typical metallic Cu (~2.6 Å). Moreover, due to the weak Cu-Cu scattering in **Cat3** spectra, its coordination number was not reliably fit, indicating the negligible presence of metallic species. This agreement suggests the assumption of coordinative Cu-N/O motifs as the primary species in all screened candidates. Table S2 compares computed Cu-Cu, Cu-N, Cu-O, and Cu-N/O bond lengths in non-protonated and protonated structures of different cluster sizes (dimer, tetramer, and octamer). The Cu-N/O bond length remains largely unchanged upon protonation,

[17] Experiment carried out in aq. 0.1 M $CsHCO_3$.

aligning with the experimental value of ~1.95 Å. In contrast, protonation increases the Cu-Cu bond distance across all clusters, exceeding the experimental value of 2.89 ± 0.02 Å. The larger Cu-Cu distance observed in the protonated octamer likely results from structural distortions in the gas-phase single-molecule model.

Table S6. A summary of structural parameters determined from the analysis of EXAFS functions from the most distinct components of the HERFD-XAS dataset resolved by MCR-ALS. ρ (%) represents the difference between the fit and the data. Fitting was performed in the range of 1 – 2.7 Å on k^1, k^2, k^3-weighted data Fourier-transformed in the k-range of 2 – 10 $Å^{-1}$.

Spectrum	Coord. shell	Coordination number (CN)	σ^2 / $Å^2$	R / Å	$\Delta\varepsilon_0$ / eV	R factor
**Component 1 (OCP)**	Cu-N/O<br>Cu-Cu	4 (S_0^2 = 0.91)[18]<br>n.a.[19]	0.006±0.003	1.92±0.02	-0.9±1.5	0.03
**Component 2 (high *j*)**	Cu-N/O<br>Cu-Cu	2.6±0.3<br>2.3±1.6	0.002 (fixed)<br>0.010±0.007	1.96±0.02<br>2.54±0.02	1.7±1.5	0.03

Table S7. Linear combination fitting analysis for Figure S69 by using different reference for signal of **Cat3** under 200 mA cm^{-2} condition.

Fitting	Cu^{2+}(%)	Cu^{+}(%)	Cu^{0}(%)	R factor	chi square
**Figure S69a**	82.2	0.3	17.4	0.007	0.06
**Figure S69b**	83.1	16.9	/	0.009	0.08
**Figure S69c**	82.2	/	17.8	0.007	0.06

[18] In lieu of a suitable reference measurement, the amplitude reduction factor was determined based on the assumption of 4 nearest neighbors in the sample at OCP.
[19] The attempt to include a Cu-Cu (metallic) shell in the fit does not improve the fit statistics while resulting in a CN of 0.3±0.4, hence it was not included in the final fit.
